# FGD3 mediates lytic cell death, enhancing efficacy and immunogenicity of chemotherapy agents in breast cancer

**DOI:** 10.1186/s13046-025-03559-5

**Published:** 2025-11-13

**Authors:** Junyao Zhu, Xinyi Dai, Santanu Ghosh, Elaine Wei, Chengjian Mao, Qianjin Jiang, Abigail J. Spaulding, Michael P. Mulligan, Roger Romero, Yoo Jane Han, Matthew W. Boudreau, Olufunmilayo Olopade, Paul J. Hergenrother, David J. Shapiro

**Affiliations:** 1 Departments of Biochemistry and Chemistry, University of Illinois at Urbana- Champaign, Urbana, IL 61801 USA; 2 Carl R. Woese Institute for Genomic Biology, University of Illinois at Urbana- Champaign, Urbana, IL 61801 USA; 3 Cancer Center at Illinois, University of Illinois at Urbana-Champaign, Urbana, IL 61801 USA; 4 Department of Medicine, The University of Chicago, Chicago, IL 60637 USA

**Keywords:** Breast cancer therapy, Immunogenic cell death, Chemotherapy response, Plasma membrane rupture, Doxorubicin, Cytoskeleton regulation

## Abstract

**Background:**

Although anticancer therapies inducing necrosis, necroptosis and pyroptosis trigger cell swelling, plasma membrane rupture (PMR) and release of damage-associated molecular patterns (DAMPs), potentially facilitating antitumor immunity, little was known of proteins and mechanisms controlling the life-death decision of whether swollen and stressed cancer cells enter PMR and undergo lytic cell death.

**Methods:**

We conducted a genome-wide CRISPR screen with selection against a lytic cell death inducer, complemented by studies using breast cancer cells in 2D culture, patient-derived organoids and orthotopic mouse xenografts. The effect of FGD3 on immunogenicity was explored by immunoblotting, immunofluorescence staining and NK-cell mediated cytotoxicity assays. The correlation between the level of FGD3 expression and patient prognosis and response to chemotherapy was assessed by analysis of patient databases.

**Results:**

We identified FGD3 as a key mediator, coupling cell swelling to PMR and lytic cell death induced by emerging and current breast cancer therapies, including ErSO, aprepitant, doxorubicin and epirubicin. FGD3 coupled cell swelling to PMR across the spectrum of immunogenic lytic cell death pathways, including necrosis, necroptosis and pyroptosis. Mechanistically, FGD3 facilitated PMR by controlling actin reorganization via the Cdc42-ARP2/3 axis. Notably, elevated FGD3 increased release of DAMPs, strongly enhanced exposure of immunogenic cell surface calreticulin and increased sensitivity of cancer cells to NK cell-mediated lysis. Supporting clinical relevance, high FGD3 expression strongly correlated with improved relapse-free survival in breast cancer patients after chemotherapy and this correlation was stronger than was seen for NINJ1 and other proteins associated with lytic cell death.

**Conclusion:**

FGD3 is a key mediator of chemotherapy-induced plasma membrane rupture and lytic cell death. It is also a useful biomarker for identifying breast cancer patients most likely to benefit from lytic cell death-inducing immunogenic anticancer therapies.

**Supplementary Information:**

The online version contains supplementary material available at 10.1186/s13046-025-03559-5.

## Introduction

Although immunotherapy has revolutionized cancer therapy, it has had very limited success against many solid tumors, which downregulate neoantigen presentation and largely lack infiltrating immune cells [1, 2]. One way to potentially enhance the ability of immunotherapy agents to target solid tumors is to combine them with immunogenic cell death (ICD)-inducing therapies that trigger plasma membrane rupture (PMR) and lytic cell death, exposing and releasing immunomodulatory damage-associated molecular patterns (DAMPs) [3, 4]. Lytic cell death pathways include regulated pyroptosis, necroptosis, or a passive and uncontrolled mode - necrosis [5, 6]. For regulated lytic cell death pathways, proteins responsible for pore formation and contributing to PMR, including MLKL for necroptosis and gasdermins for pyroptosis, have been extensively studied [7, 8]. Recently, NINJ1 was shown to play a pivotal role in PMR of macrophages during pyroptosis [9–11]. However, in uncontrolled lytic cell death elicited by anticancer therapies, the end stage protein(s) that couple cell swelling to induction of PMR and subsequent exposure and release of DAMPs remain poorly described.

Many anticancer agents exhibit a mixed phenotype in which apoptotic and necrotic pathways both contribute to cell death. To probe necrotic cell death pathways using a small molecule that exclusively induces necrosis-related cell death, we used our promising preclinical anticancer agent, ErSO [12]. ErSO initially induces phosphorylation of Src and PLC-γ and lethal hyperactivation of the anticipatory unfolded protein response (a-UPR), resulting in robust inhibition of protein synthesis, increased intracellular calcium and ATP depletion, and then triggers opening of the plasma membrane TRPM4 sodium channel, leading to cell swelling and PMR [13–17]. Since pan-caspase inhibitors and necroptosis inhibitors do not block ErSO-induced cell death and there are no known pore-forming proteins required for ErSO-induced PMR, ErSO induces necrosis [15, 16]. To explore how cells respond to stress-induced cell swelling and make the life-death decision of plasma membrane rupture and cell death, or survive the stress, we carried out a genome-wide CRISPR-Cas9 knockout screen in human breast cancer cells with positive selection against ErSO [18, 19].

The top target from the CRISPR screen was FYVE, RhoGEF and PH domain-containing protein 3 (FGD3). FGD3 is highly expressed in breast cancer as a favorable prognostic marker, but its role in breast cancer is largely unstudied [20–22]. FGD3 is reported to be a guanine nucleotide exchange factor (GEF) of the Rho GTPase Cdc42 [23, 24]. Cdc42 connects diverse signals to actin reorganization through its downstream effectors WASP and the ARP2/3 complex, which promote actin branching [25–27]. As a GEF of Cdc42, FGD3 was suggested to activate Cdc42 and regulate actin reorganization to induce lamellipodia formation [24].

Here, we explore the role of FGD3 in lytic cell death in breast cancer. Interestingly, FGD3 does not regulate any of the steps related to ErSO-induced a-UPR activation and cell swelling, but instead couples cell swelling to PMR and cell death. Under ErSO-induced cell swelling, FGD3 activates Cdc42 and its downstream effector, the ARP2/3 complex, to reorganize the actin network in a way that facilitates exposure of immunomodulatory cell surface calreticulin and PMR, resulting in release of cellular contents including lactate dehydrogenase (LDH, a PMR marker), HMGB1 and ATP (widely studied DAMPs) [28]. Consequently, FGD3 level is strongly correlated with cancer cell sensitivity to ErSO-induced lytic cell death in 2-dimensional (2D) cell culture, 3-dimensional (3D) patient-derived breast cancer organoids (PDOs) and orthotopic mouse xenografts.

Notably, our work further explores FGD3 as a broad mediator of PMR in both regulated and unregulated lytic cell death pathways, including necroptosis and pyroptosis. FGD3 knockout cells show attenuated responses to inducers of necrosis, necroptosis, and pyroptosis, leading to impaired PMR, decreased DAMP release, and ultimately less cell death. These data suggested current anticancer therapies that work in part through necrosis, necroptosis and pyroptosis might have a previously unexplored dependence on FGD3. Remarkably, pyroptosis induced by the widely used chemotherapy drugs doxorubicin and epirubicin strongly correlates with FGD3 levels [29–32]. Supporting clinical relevance, database analysis shows a strong positive correlation between FGD3 level and a favorable response to chemotherapy in breast cancer patients. These data suggest that a high level of FGD3 is a favorable prognostic marker in breast cancer because it enhances the effectiveness of lytic cell death-inducing anticancer therapies. Our study suggests FGD3 levels can be used to identify cancer patients most likely to benefit from doxorubicin, and other chemotherapy agents that induce cell swelling, PMR and ICD.

## Materials and methods

### Cell culture and reagents

MCF-7 cells were from the Michigan Cancer Foundation (MCF). T47D cells were provided by Professor K. Horwitz (University of Colorado, Denver, CO). Before use in these studies, cell lines were verified by genotyping at the University of Arizona facility and were negative for Mycoplasma using a PCR assay. Luciferase-expressing cells used for xenografts were produced in our laboratory, as described previously [16]. All cell lines were cultured at 37^°^C with 5% CO_2_ in phenol red-free medium [medium: MCF-7 (MEM, 5% FBS), T47D (MEM, 10% FBS), THP-1 (RPMI-1640, 10% FBS)]. All growth media were supplemented with 1% penicillin-streptomycin. NK-92 cells (provided by Dr. C. Froehlich, Evanston Hospital, Evanston IL) were cultured as previously described [33]. The following reagents were used: doxorubicin (#2252 Tocris Bioscience), epirubicin (#S1223 Selleckchem), shikonin (# S8279 Selleckchem), 35 S-methionine (#NEG709A500UC Revvity); all other reagents were from Sigma-Aldrich: staurosporine (#S4400), Raptinal (#SML1745), 2-deoxyglucose (#D8375), aprepitant (#SML2215), ML141 (#SML0407), CK-666 (#SML0006), blebbistatin (#B0560), Y-27632 (#Y0503).

### CRISPR-Cas9 screen

150 million MCF-7 cells were plated onto 25 15-cm tissue culture dishes and were infected with the viral library at a multiplicity of infection of 0.3. The cells were selected with 2 mg/mL puromycin for 3 days. Surviving cells were harvested and 1.5 × 10^7^ cells were frozen as the day 0 control sample. The remaining cells were plated evenly into four T175 flasks (four biological replicates) and treated with 100 nM ErSO for 28 days. The surviving cells were harvested, and four samples of 1.5 × 10^7^ cells from each biological replicate were obtained. Genomic DNA from the four samples and the control sample was extracted, and the sgRNA region was amplified using PCR. Subsequently, sgRNA libraries were amplified using i5 and i7 primers and then sequenced on Illumina HiSeq4000. The sequence data were analyzed using the MAGeCK-VISPR algorithm [34].

### Cell proliferation assay

2,000 cells in 90 µl of medium were plated into each well of a 96-well plate. The next day, 10 µl of medium containing the indicated treatment was added to each well. The medium was changed again and replaced with treatment-containing medium after 2 days. Proliferation was assayed after 4 days. 10 µl of alarmaBlue (#DAL1100 Thermo Fisher Scientific) was added per 100 µl of medium, and the cells were incubated at 37^°^C with 5% CO_2_ for 1 h. Fluorescence intensity was measured using a PHERAstar Microplate Reader (BMG Labtech Inc.). MTS assays were performed as described previously [13].

### Lentiviral production and clonal selection

The following plasmids: pMD2.G (#12259) and psPAX2 (#12260), Toronto KnockOut CRISPR Library, version 3 (#90294), LentiCRISPRv2 puro (#98290), pCDH-puro-cMyc (#46970), pLKO.1 – TRC (#10878) were from Addgene. gRNA sequences for knockout were cloned into LentiCRISPRv2 puro (sequences for FGD3: GAGCGCCTGAAGCTTCCCTA, CCAGGAGCTCCTGCACACCG, GCTGAAGACGCGGATCACGG, CACGAAGGGACACAAACCCA). shRNA sequences for knockdown were cloned into pLKO.1 – TRC (sequences for Cdc42 sh1: GTGAGGGAAATATACAATTGT, Cdc42 sh2: TTCAGCAATGCAGACAATTAA; sequences for ARP2 sh1: CAGGGTGACATAATGCATTTA, ARP2 sh2: TTGGACCAGAGAAACTTAATA). FGD3 overexpression plasmid was made by cloning FGD3 cDNA (TransOmic technologies) into pCDH-puro-cMyc. Lentiviral packaging vectors were co-transfected with the required plasmid into HEK293T cells using XtremeGene 360 (#XTG360-RO Roche). The next day, the medium was changed to virus harvest medium: DMEM + 1.1 g/mL BSA. The virus was harvested 24 h later and used to transduce cells for 48 h before selection with cell line–specific concentrations of puromycin. For MCF-7 FGD3 knockout cells, individual colonies were picked and grown out. All the other lentiviral-transduced cells were stable pools of cells.

### Trypan blue dye exclusion death assay and swelling assay

Cell viability and cell diameter after treatment with vehicle or compounds were measured using a Countess II cell counter (Thermo Fisher Scientific) as previously described [15, 16]. 300,000 cells/well were plated in 6-well plates. The next day, the vehicle or treatment was added for the indicated time. Cells were then harvested and concentrated to 1–5 × 10^6^ cells/ml. 10 µl of trypan blue was mixed with 10 µl of cells immediately before counting for cell viability, or measurement of cell diameter.

### ATP measurement

Assays of cellular ATP levels were performed as previously described [15]. The level of ATP released into the supernatant was assayed by collecting the supernatant followed by centrifugation at 1,500 rpm for 3 min. Then, the ATP levels were measured using the ATPlite Luminescence Assay Kit protocol (#6016941 Revvity).

### Lactate dehydrogenase release measurement

10,000 cells in 90 µl medium were plated into each well of a 96-well plate. The next day, 10 µl medium containing the indicated treatment was added to each well. After 24 h, a specific amount of supernatant was removed and mixed with the LDH storage buffer (200mM Tris-HCl pH 7.3, 10% Glycerol, 1% BSA). The LDH levels were then measured using the LDH-Glo™ Cytotoxicity Assay protocol (#J2381 Promega).

### Western blotting

Western blotting was performed as previously described [13]. To examine the level of HMGB1 released into the medium, 300,000 cells were plated in each well in a 6-well plate. Next day, the medium was removed and 1 ml of medium containing the indicated treatment was added into the well. After 24 h, the 1 ml of medium from each well was collected, centrifuged at 1,500 rpm for 5 min to remove any floating or dead cells. The supernatant was then transferred into a new Eppendorf tube for subsequent western blotting. Each lane contained the same volume of the collected supernatant. The following antibodies were used: FGD3 (Proteintech, 20347-1-AP, RRID: AB_10693616), phospho-Src (Cell Signaling Technology, 6943, RRID: AB_10013641), Src (Cell Signaling Technology, 2109, RRID: AB_2106059), phospho-eIF2α (Cell Signaling Technology, 3398, RRID: AB_2096481), eIF2α (Cell Signaling Technology, 5324, RRID: AB_10692650), β-actin (Cell Signaling Technology, 3700, RRID: AB_2242334), α-tubulin (Cell Signaling Technology, 2144, RRID: AB_2210548), HMGB1 (Abnova, H00003146-M08, RRID: AB_518856), Cdc42 (Cell Signaling Technology, 2462, RRID: AB_2078085), ARP2(Cell Signaling Technology, 3128, RRID: AB_2181763), TRPM4 (OriGene, TA500381, RRID: AB_2208624), ERα (Santa Cruz Biotechnology, sc-8002, RRID: AB_627558), vinculin (Cell Signaling Technology, 4650, RRID: AB_10559207). Antibodies were probed with horseradish peroxidase (HRP)-conjugated secondary antibodies (Thermo Fisher Scientific) and imaged with the Super ECL Ultra Kit (#FP302 ABP Biosciences) using the iBright CL1000.

### Immunofluorescence staining and confocal microscopy

40,000 cells in 1 ml of medium were plated onto a coverslip in each well of a 24-well plate. The next day, the medium was replaced with the medium containing the indicated treatment. After one PBS wash, the cells on the coverslip were fixed using 4% paraformaldehyde and permeabilized using 0.1% Triton X-100 in PBS. Then the cells were stained with Acti-Stain 488 Phalloidin (#PHDG1 Cytoskeleton, lnc.) for F-actin visualization or stained with ARPC2 primary antibody (Abcam, ab133315) followed with Alexa Fluor 594-conjugated secondary antibody (Jackson Immuno) for ARP2/3 complex visualization. The stained samples were mounted onto the slides using ProLong™ Glass Antifade Mountant with NucBlue™ Stain (#P36983 Invitrogen). The slides were then imaged using a ZEISS LSM 700 confocal microscope.

For surface calreticulin visualization, the cells were fixed with cold methanol and not permeabilized throughout the whole process. The cells were stained with calreticulin primary antibody (Abcam, ab92516, RRID: AB_10562796) followed with Alexa Fluor 594-conjugated secondary antibody (Jackson Immuno). All the other steps were the same as described above.

### THP-1 migration assay

Cells were plated in a 6-well plate at 360,000 cells/well in complete medium. The next day, cells were treated with vehicle or 100 nM ErSO for 4 h. The cells were washed once with ErSO-free medium and supplemented with fresh ErSO-free medium. The cells were then incubated for another 20 h. After incubation, the medium was collected from all the treatment groups. 200,000 THP-1 human monocytes were seeded in Millipore polycarbonate cell culture inserts (5-µm pores). Vehicle medium (500 µl), 5% FBS medium, or previously collected conditioned medium was pipetted into the bottom chamber. Migration was allowed to proceed for 4 h. The bottom chamber medium was transferred to a 96-well plate, and an alamarBlue assay was performed to measure the number of live cells that passed through the membrane using the PHERAStar plate reader (BMG Labtech).

### NK cell-mediated cytotoxicity assay

10,000 Luciferase-expressing cancer cells/well were plated in a 96-well plate. The next day, cancer cells were treated with vehicle or 100 nM ErSO for 2 h. The cells were washed once with ErSO-free medium. The NK-92 cells were then loaded into each well at different E: T (effector: target) ratios and cocultured with the cancer cells for 3 h. Wells were then washed once with 1×PBS. 30 µl Bright-Glo luciferase assay reagent (#E2620 Promega) was added into each well and the luminescence was read using the PHERAStar plate reader. The cytotoxicity % was calculated as the following formula:

100 – [(reading from wells with NK-92 cells/reading from wells without NK-92 cells] × 100.

### Orthotopic mouse xenograft study

The animal study was approved by the University of Illinois Institutional Animal Care and Use Committee (protocol #22096). NSG ovariectomized mice were obtained from The Jackson Laboratory (005557). A 0.36 mg 17β-estradiol pellet (Innovative Research of America) was implanted subcutaneously into each mouse using a trochar. After 2 days, luciferase-expressing MCF-7, MCF-7-FGD3 overexpression, and MCF-7-FGD3 knockout cells (1,250,000 cells in 1:1 Hanks’ Balanced Salt Solution: Matrigel) were inoculated into the mammary fat pad. When the tumors grew to approximately 100 mm^3^ (∼28 days to establish tumors), mice in each group with the same cell type were then randomized and treated with vehicle (*n* = 3) or 10 mg/kg ErSO i.p. (*n* = 3) on day 0. Bioluminescence Imaging (BLI) of the tumors was conducted on days 0, 3, and 7. Mice were also weighed on days 0, 3, and 7.

### 3D breast cancer patient-derived organoid (PDO) culture

The procurement of biospecimens for the generation of breast cancer patient-derived organoids (PDOs) has been performed according to the approved institution review board protocol (16352 A) at the University of Chicago with a signed patient consent [35]. The breast cancer PDO protocol is adapted from a previous study [36]. The complete medium recipe is included in the supplement (Table S2). For regular maintenance of PDOs, ~ 10,000 cells were plated in 60 µl Matrigel per well in 24-well suspension plates (Greiner). After 5 min at RT and 25 min at 37 °C to solidify the Matrigel, 1 ml of medium was added to each well. A medium change was carried out every 3–4 days. For total RNA and total protein preparation, about 0.1 million cells in 250 µl Matrigel per well were seeded to the center of a 6-well suspension culture plate (Greiner). After the Matrigel was solidified at 37 °C, 4 ml of medium was added to each well. Cells were grown for 8 days with one medium change to form organoids. Treatments were added directly to the well. To harvest the organoids, 3 ml ice-cold 1×PBS was added to each well after removing the medium. After the Matrigel was disturbed by pipetting, the mixture of cells was transferred to an ice-cold tube. The cells were washed with ice-cold PBS again and the wash was combined with the cell mixture. After vigorously shaking, the tube was put on ice for about 5 min. The pellet of organoids was collected by centrifuging at 125 g for 6 min. and then used for total RNA or protein preparation. For cell growth assays, about 1,000–2,000 single cells in 10 µl of Matrigel per well were plated in the upper-corner of the 96-well suspension plate. The cells were grown for 14 days to form organoids with a medium change every 3–4 days. Treatments were added in fresh medium and incubated for 7 days with one medium change. MTS assays were used to measure cell viability.

### qRT-PCR

qRT-PCR was conducted as previously described [13]. The reaction was conducted using a QuantStudio 3 Real-Time PCR System (Applied Biosystems). Each gene’s fold change in expression was calculated using the ∆∆C_t_ method with the ribosomal protein 36B4 as the internal control (Table S3).

### Breast cancer cell migration and invasion assay

Millipore polycarbonate cell culture inserts (8-µm pores) were either uncoated (for migration assays) or coated with Matrigel (for invasion assays) and placed in a 24-well plate. Cells were estrogen-depleted by maintenance in 5% CD-FBS for 4 days in advance. The cells were then harvested and resuspended at 200,000 cells/ml in medium without FBS containing 0.1% BSA. 0.5 ml of the harvested cells were placed in the upper chamber, and 0.55 ml medium containing 5% FBS was placed at the bottom of the well. After 24 h, the supernatant in the upper chamber was removed, and the membrane was cleaned using cotton tips. alamarBlue assay was performed to measure the number of live cells that passed through the membrane with fluorescence monitored using a PHERAStar plate reader (BMG Labtech).

### Live cell imaging

Cells were pre-stained by 250 nM SytoxGreen (#S7020 ThermoFisher Scientific) for 30 min. The cells were then treated with 1 µM ErSO and immediately put in the micro cell incubator. The time-lapse images were taken using a Leica DMI8 microscope.

### Statistics

Information on statistical analysis is in the legends of individual figures. The Unpaired Student t test and Two-way ANOVA were used to compare the variables between groups using GraphPad Prism. All graphs are the mean plus or minus the SEM unless stated otherwise in the figure legend [statistical significance: ns, not significant; *, *P* < 0.05; **, *P* < 0.01; ***, *P* < 0.001; ****, *P* < 0.0001].

## Results

### A CRISPR screen identifies FGD3 as important in necrotic cell death of breast cancer cells

**Fig. 1:**
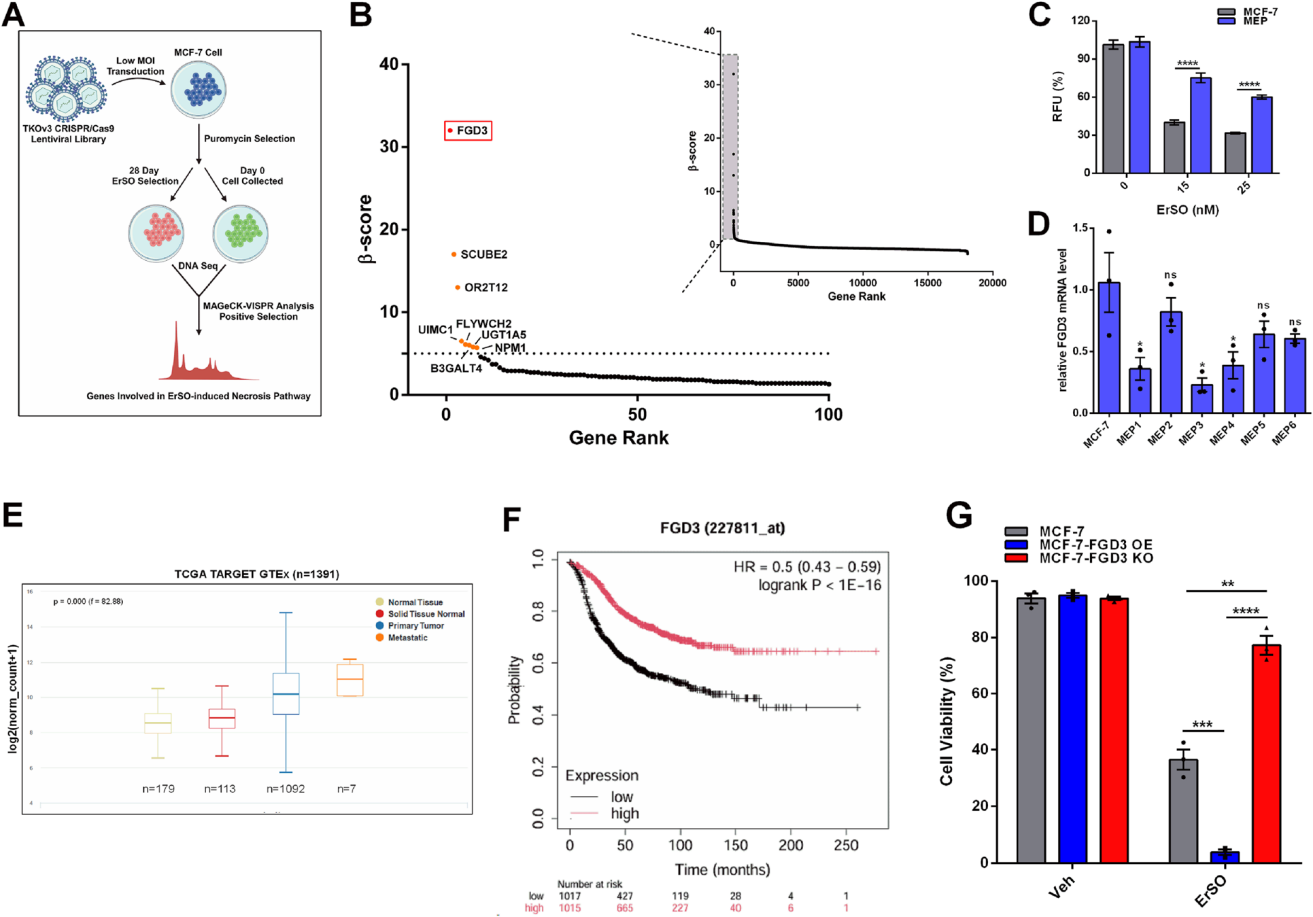
FGD3 plays a crucial role in ErSO-induced necrosis. **A**, Schematic flow chart of the genome-wide CRISPR-Cas9 screen with positive selection against ErSO in MCF-7 cells. **B**, A genome-wide CRISPR-Cas9 screen with positive selection against ErSO identified FGD3 as the top target. **C**, 4-day alamarBlue cell proliferation assay of wild-type and ErSO-resistant MCF-7 cells treated with the indicated concentrations of ErSO (*n* = 6). **D**, qRT-PCR analysis of FGD3 expression level in wild-type and ErSO-resistant MCF-7 cells (*n* = 3). **E**, Analysis of data from the TCGA database showing relative FGD3 expression levels in normal breast tissue, solid breast tissue normal, primary and metastatic breast tumors. **F**, KM Plotter online platform analysis showing the effect of FGD3 on recurrence free survival of BRCA patients. **G**, Automated trypan blue exclusion assay comparing viability of wild-type MCF-7, MCF-7-FGD3 OE (overexpression) and MCF-7-FGD3 KO (knockout) cells treated with vehicle or 100 nM ErSO for 24 h (*n* = 3). All data are mean ± s.e.m. *p < 0.05, **p < 0.01, ***p < 0.001, ****p < 0.0001, ns = not significant by Student’s t-test

To explore unregulated PMR and lytic cell death in cancer cells, we used the preclinical anticancer agent, ErSO, which induces hyperactivation of the a-UPR pathway, ATP depletion, cell swelling and PMR (Fig. S1A; [12, 13, 16]). Compared to other inducers of diverse classes of lytic cell death, a relatively low concentration of ErSO triggered comparable levels of LDH and HMGB1 release in MCF-7 human breast cancer cells and elicited exclusively necrotic cell death morphology (Fig. S1B-D), making ErSO an appropriate agent to use for exploring potential mediators of PMR and lytic cell death in breast cancer cells. We therefore did a genome-wide, CRISPR-Cas9 knockout screen in MCF-7 cells with positive selection against ErSO (Fig. 1A). The quality of the CRISPR screen was high (Fig. S1E, F). Interestingly, a GEF of Cdc42, FGD3, was the top target (Fig. 1B and Table S1). Moreover, in clones of ErSO-resistant MCF-7 cells which exhibit a persister cell phenotype with robust Myc downregulation and limited proliferation (Fig. 1C and Fig. S1G; [37, 38]), qRT-PCR shows FGD3 mRNA was downregulated (Fig. 1D). Although the analysis of TCGA and online KM Plotter showed FGD3 is highly expressed in breast cancer and several other cancers and is a favorable prognostic marker (Fig. 1E, F and Fig. S1H; [39–41]), it has been little studied. Compared to normal breast tissue, FGD3 is highly expressed in both non-metastatic and metastatic tumors, but there is not a strong correlation between FGD3 level and tumor metastasis status (Fig. S2A). To explore the role of FGD3 in ErSO-induced lytic cell death, we began by producing MCF-7 and T47D human breast cancer cells in which FGD3 was knocked out or overexpressed (Fig. S2B). FGD3 overexpression slightly promoted cell proliferation (Fig. S2C) and FGD3 knockout significantly decreased cell migration and invasion in MCF-7 cells (Fig. S2D, E; [42]). Notably, in an automated Trypan Blue exclusion assay for cell viability, FGD3 overexpression strongly increased the breast cancer cells sensitivity to ErSO, while FGD3 knockout rendered the cells resistant to ErSO-induced rapid cell death (Fig. 1G and Fig. S2F). Our laboratory recently reported ErSO is also a promising necrosis-inducing therapy in ovarian cancer [17]. Therefore, it was of interest to explore the effect of different FGD3 levels on sensitivity of ovarian cancer cells to ErSO. We compared FGD3 levels to sensitivity of 4 human ovarian cancer cell lines (IGROV-1, SK-OV-3, ES-2 and PEO4) to ErSO-induced cell death. IGROV-1, SK-OV-3 cells, which had trace levels of FGD3 (by Western blot) were nearly insensitive to ErSO, while ErSO-treated ES-2 and PEO4 cells, which had higher FGD3 levels, exhibited a 30–40% decrease in cell viability (Fig. S2G, H). Of course, given the heterogeneity of these ovarian cancer cell lines, FGD3, while important, will only be one factor modulating sensitivity to ErSO-induced cell death. Since FGD3 expression is highly elevated in breast cancer (Fig. S1H), here we focus on its role in breast cancer. Taken together, these data strongly support a crucial role for FGD3 in the ErSO-induced necrosis.

### FGD3 level is correlated with the survival of stressed cancer cells treated with ErSO

We next explored whether FGD3 is mediating ErSO-induced cellular stresses contributing to cell death. FGD3 does not regulate the initiation of the pathway as shown by similar levels of p-Src in ErSO-treated wild-type, FGD3 overexpressing and FGD3 knockout cells (Fig. S3A, B). An additional important early step in the ErSO pathway is an increase in intracellular calcium. Live cell calcium imaging demonstrated that altering the FGD3 level did not affect the initial increase in cytosol calcium elicited by ErSO (Movies S1-S3). We then examined the effect of different levels of FGD3 on ErSO-induced a-UPR hyperactivation (phosphorylation of eIF2α and induction of spliced XBP1), protein synthesis inhibition, ATP depletion, and cell swelling [15, 16]. In MCF-7 and T47D cells, neither the a-UPR pathway nor the cellular stresses were regulated by modulation of FGD3 levels (Fig. S3A-F). These data indicated FGD3 does not impact cellular stress in the early or intermediate stages of ErSO-induced cell death.

**Fig. 2:**
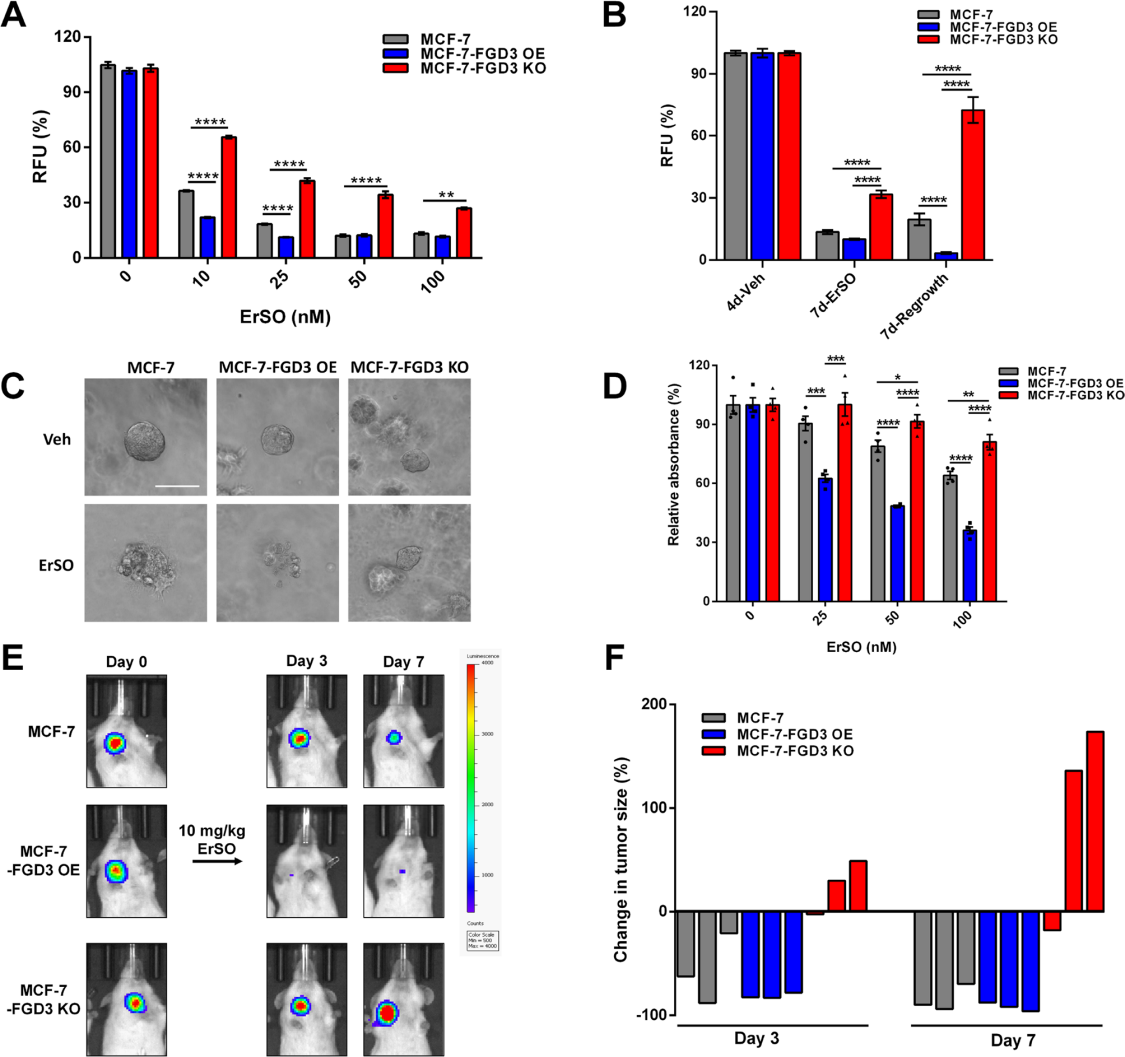
FGD3 level makes the decision for stressed cancer cells treated with ErSO to survive or undergo cell death. **A**, 4-day alamarBlue cell proliferation assay comparing survival of MCF-7, MCF-7-FGD3 OE and MCF-7-FGD3 KO cells treated with the indicated concentrations of ErSO (*n* = 6). **B**, 14-day cell regrowth assay comparing survival of MCF-7, MCF-7-FGD3 OE and MCF-7-FGD3 KO cells treated with 100 nM ErSO for 7 days and then allowed to recover in fresh medium without ErSO for another 7 days (*n* = 6) **C**, Bright field image of 3-dimensional (3D) MCF-7, MCF-7-FGD3 OE and MCF-7-FGD3 KO organoids treated with vehicle or 100 nM ErSO for 4 days (scale bar 50 μm). **D**, 4-day MTS cell proliferation assay comparing survival of MCF-7, MCF-7-FGD3 OE and MCF-7-FGD3 KO organoids treated with the indicated concentrations of ErSO (*n* = 4). **E**, Orthotopic luciferase-expressing MCF-7, MCF-7-FGD3 OE and MCF-7-FGD3 KO tumors were established in breasts of ovariectomized NSG mice, grown for 28 days, and then treated with a single dose of 10 mg/kg ErSO i.p. on day 0 (*n* = 3). Shown are representative bioluminescence images of tumors on day 0, 3 and 7. **F**, Waterfall plot showing the change in tumor size as percentage of the initial flux for each mouse in all three ErSO treated groups. All data are mean ± s.e.m. *p < 0.05, **p < 0.01, ***p < 0.001, ****p < 0.0001, by Student’s t-test

We then explored FGD3’s effect on cancer cell survival during longer-term ErSO-induced stress. As expected, MCF-7 and T47D cells in which FGD3 was knocked out exhibited increased survival compared to wild-type and FGD3 overexpression cells (Fig. 2A and Fig. S3G) and showed limited proliferation at higher ErSO concentrations. In a regrowth experiment, cells were treated with ErSO for 7 days, followed by 7 days in ErSO-free fresh medium. Notably, the MCF-7-FGD3 KO cells demonstrated robust regrowth, the wild-type MCF-7 cells showed minor regrowth, and MCF-7-FGD3 OE cells were eradicated by ErSO and showed no regrowth (Fig. 2B). These data show that the FGD3 knockout cells can survive and quickly recover from ErSO-induced stress.

The loss of FGD3 similarly prevented cell death but not decreased proliferation in more physiologically relevant models that better mimic the 3D tumor environment – 3D breast cancer cell organoids and orthotopic mouse xenografts. MCF-7, MCF-7-FGD3 OE and MCF-7-FGD3 KO cells were grown in serum-free medium in Matrigel to establish 3D organoids [36]. After 4 days of ErSO treatment, the MCF-7 and MCF-7-FGD3 OE organoids exhibited disrupted integrity and morphology typical of necrotic cell death. In contrast, MCF-7-FGD3 KO organoids maintained their integrity (Fig. 2C) and demonstrated better survival (Fig. 2D). In an orthotopic mouse xenograft model using luciferase-based bioluminescence imaging to visualize tumors, ErSO induced rapid, near-complete tumor regression in all 3 MCF-7-FGD3 OE tumors by day 3, while ErSO-induced tumor regression in the MCF-7 tumors was more varied at day 3 and largely caught up by day 7. However, 2 of 3 FGD3 KO tumors displayed reduced but continued growth on day 7 (Fig. 2E, F and Fig. S4A-F).

Taken together, these data demonstrate that FGD3 level correlates with the survival of stressed cancer cells without regulating the stresses contributing to cell death. These data also indicated that FGD3 may act at a late stage in the cell necrosis.

### FGD3 mediates the last stage of cell death - plasma membrane rupture and the subsequent release of immunomodulatory DAMPs

**Fig. 3:**
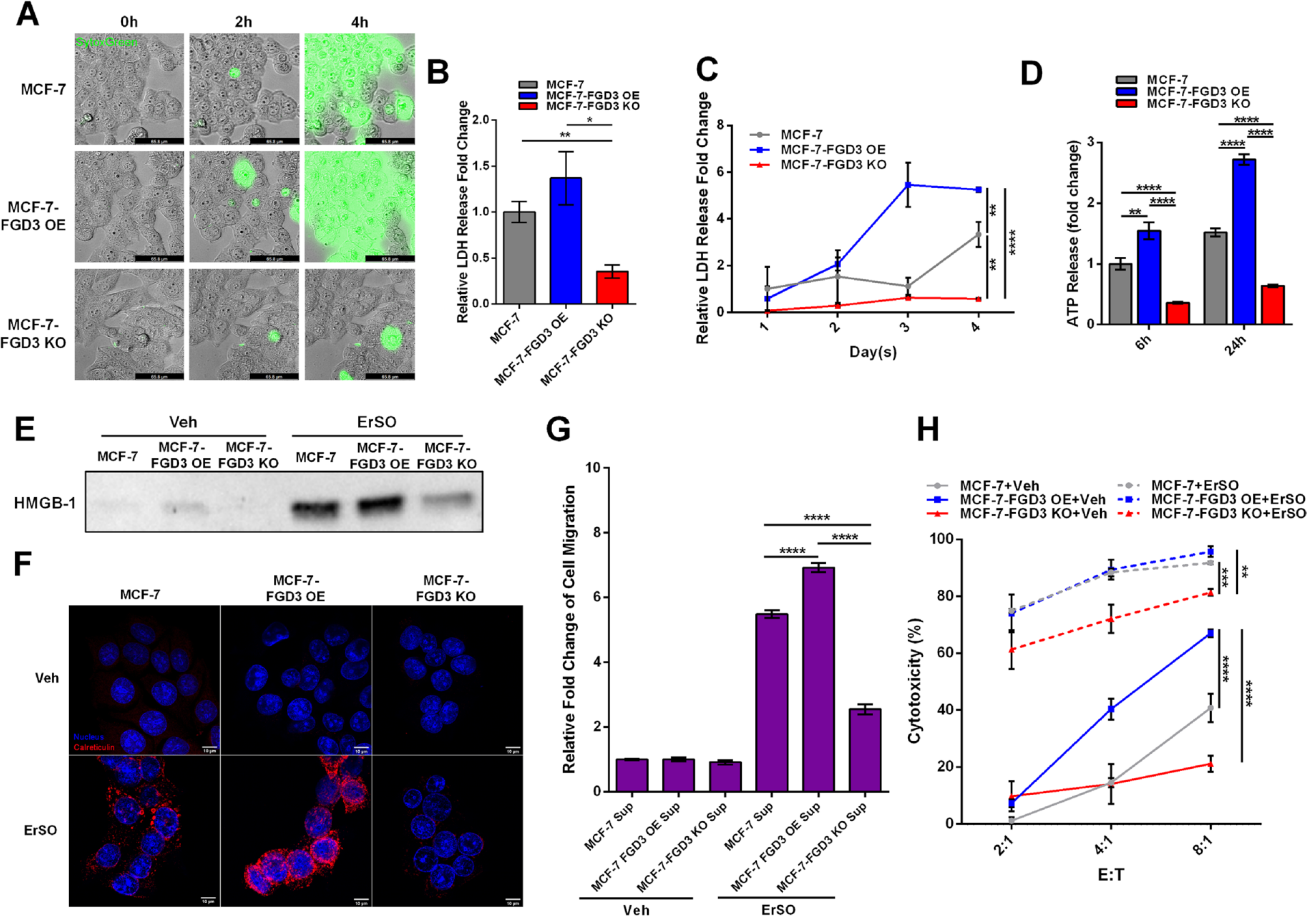
FGD3 mediates plasma membrane rupture and subsequent release and exposure of DAMPs. **A**, Time-lapse images of MCF-7, MCF-7-FGD3 OE and MCF-7-FGD3 KO cells pre-stained with SytoxGreen and then treated with 1 µM ErSO (scale bar 65.8 μm). **B**, Relative fold change of lactase dehydrogenase (LDH) released into the medium from MCF-7, MCF-7-FGD3 OE and MCF-7-FGD3 KO cells treated with 100 nM ErSO for 24 h (*n* = 3). **C**, 4-day relative fold change of LDH released from 3D MCF-7, MCF-7-FGD3 OE and MCF-7-FGD3 KO organoids treated with 100 nM ErSO (*n* = 3). **D**,** E**, Release into the medium from MCF-7, MCF-7-FGD3 OE and MCF-7-FGD3 KO cells treated with 100 nM ErSO of **D**, ATP: (6- and 24-hour treatment [*n* = 6]); **E**, HMGB1: Western blot analysis of HMGB1 in medium (24-hour treatment). **F**, Immunofluorescence images of calreticulin exposure on MCF-7, MCF-7-FGD3 OE and MCF-7-FGD3 KO cells treated with vehicle or 250 nM ErSO for 2 h. Red, calreticulin; Blue, nucleus (scale bar 10 μm). **G**, 4-hour transwell migration assay of undifferentiated human THP1 monocytes attracted by the indicated conditioned medium. The viable migrated THP1 cells were stained with alarmaBlue and quantified (MCF-7 sup. set to 1, *n* = 6). **H**, Luciferase-expressing MCF-7, MCF-7-FGD3 OE and MCF-7-FGD3 KO cells (T-target) were treated by vehicle or 100 nM ErSO for 2 h and then cocultured with NK-92 cells (E-effector) at indicated E: T ratios for another 3 h. The cytotoxicity was determined by the luciferase activity in the surviving target cells. All data are mean ± s.e.m. *p < 0.05, **p < 0.01, ***p < 0.001, ****p < 0.0001 by Two-way ANOVA (**C**,** H**) or Student’s t-test (**B**,** D**,**G**)

Although the cells with different levels of FGD3 experienced similar levels of a-UPR hyperactivation, intracellular ATP depletion and cell swelling caused by ErSO (Fig. S3A-F), live cell imaging with SytoxGreen revealed that FGD3 knockout blocked PMR, preventing SytoxGreen from entering the cells and FGD3 overexpression facilitated PMR, resulting in more stained cells (Fig. 3A). A hallmark of PMR and necrosis is release of cellular contents, such as the PMR marker LDH, and classic immune-cell-activating DAMPs, HMGB1 and ATP. In both 2D cell culture and organoids of FGD3 knockout cells, there was little release of LDH into the medium (Fig. 3B, C and Fig. S5A). In contrast, especially in the 3D organoids, the MCF-7-FGD3 OE organoids exhibited a more rapid and dramatically increased release of LDH into the medium (Fig. 3C). Consistent with their resistance to ErSO induced plasma membrane disruption, FGD3 knockout cells exhibited little release of HMGB1 and ATP (Fig. 3D, E and Fig. S5B). Cell surface exposure of calreticulin, which is recognized by immune cells, is another feature of stress-induced necrotic cell death [43–45]. Importantly, ErSO elicited strong calreticulin exposure in MCF-7 and MCF-7-FGD3 OE cells probably due to membrane disruption, while FGD3 knockout almost abolished calreticulin exposure (Fig. 3F). Taken together, these data suggest that FGD3 is responsible for the terminal life-death decision in stressed cells treated with ErSO.

Since FGD3 level strongly impacts the exposure and release of immunomodulatory DAMPs, calreticulin, HMGB1 and ATP, we next explored the effect of FGD3 level on the immunogenicity of ErSO-induced cell swelling and necrosis. Since we and others have found that compounds based on the ErSO chemical scaffold do not induce TRPM4 dependent cell swelling and lytic cell death in mouse cancer cells [46], we could not assess the effect of FGD3 on immunogenicity in syngeneic mouse models. We therefore explored the effect of FGD3 levels on immunogenicity using human immune cells. In a THP-1 human monocyte migration assay using medium collected from ErSO-treated cells, there was far less migration of THP-1 cells exposed to the medium from MCF-7-FGD3 KO cells than those from MCF-7 and MCF-7-FGD3 OE cells (Fig. 3G). Calreticulin exposure on ER-stressed cancer cells is recognized by natural killer (NK) cells, stimulating NK-mediated cell lysis [45]. We therefore cocultured MCF-7, MCF-7-FGD3 OE and MCF-7-FGD3 KO cells with human NK-92 natural killer cells, which are approved for use in clinical immunotherapy trials, and explored NK cell-mediated cytotoxicity. Interestingly, in untreated healthy cancer cells, NK-92 cells showed strong cytotoxicity in MCF-7-FGD3 OE cells (~ 60%) and significantly less cytotoxicity in MCF-7-FGD3 KO cells (~ 20%). In ErSO-treated stressed breast cancer cells, NK-92 cells killed almost all the MCF-7 and MCF-7-FGD3 OE cells, while they showed less cytotoxicity against MCF-7-FGD3 KO cells (Fig. 3H). The elevated level of DAMPs released, and the more significant attraction and stimulation of immune cells further establish a high FGD3 level as a potential target to enhance immunotherapy by ICD-inducing therapies.

### FGD3 mediates cell swelling-induced F-actin reorganization through the Cdc42-ARP2/3 axis

**Fig. 4:**
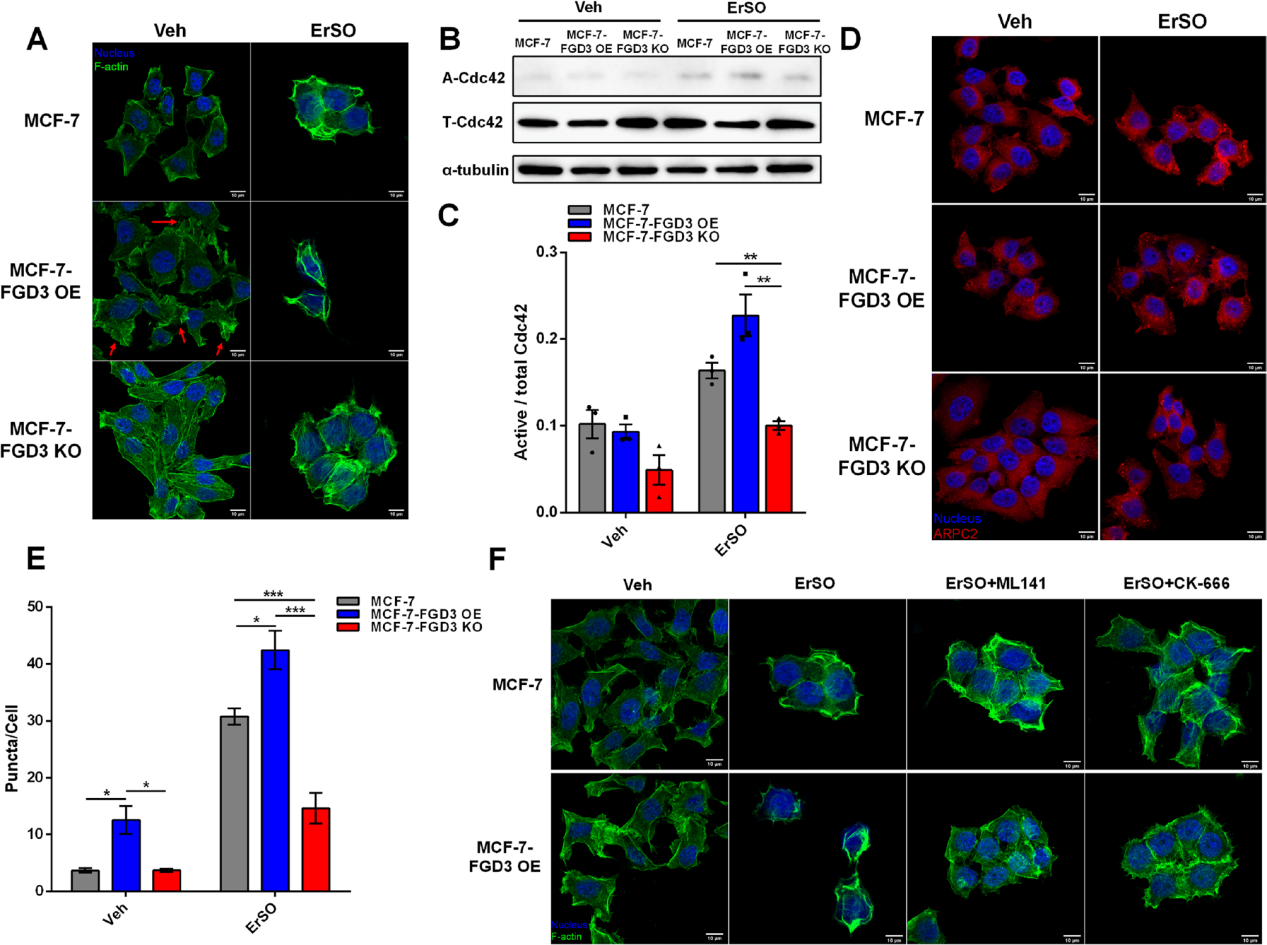
FGD3 mediates cell swelling-induced F-actin reorganization through the Cdc42-ARP2/3 axis. **A**, Immunofluorescence image of MCF-7, MCF-7-FGD3 OE and MCF-7-FGD3 KO cells treated with vehicle or 1 µM ErSO for 1 h. Green, F-actin; Blue, nucleus; Red arrows, lamellipodium (scale bar 10 μm). **B**, Western blot analysis of a GTP-Cdc42 pull down comparing active Cdc42 level in MCF-7, MCF-7-FGD3 OE and MCF-7-FGD3 KO cells treated with vehicle or 1 µM ErSO for 30 min. **C**, Summary of active Cdc42 levels (*n* = 3). **D**, Immunofluorescence images of ARP2/3 complex puncta formed in MCF-7, MCF-7-FGD3 OE and MCF-7-FGD3 KO cells treated with vehicle or 1 µM ErSO for 30 min. Red, ARPC2 (ARP2/3 complex subunit); Blue, nucleus (scale bar 10 μm). **E**, Summary of the ARP2/3 complex puncta counted in the vehicle and treated cells (*n* ≥ 30). **F**, Immunofluorescence images of MCF-7 and MCF-7-FGD3 OE cells treated for 1 h with ErSO, or ErSO plus the indicated Cdc42 inhibitor, ML141 or the ARP2/3 inhibitor, CK-666. (ErSO, 1 µM; ML141, 10 µM; CK-666, 100 µM; scale bar 10 μm). All data are mean ± s.e.m. *p < 0.05, **p < 0.01, ***p < 0.001, by Student’s t-test

How FGD3 influences PMR was unresolved. In ErSO-resistant MCF-7 clones exhibiting downregulation of FGD3 expression, RNA-seq showed differential expressions of genes related to the membrane and cytoskeleton (Fig. S5C, D). This suggested FGD3 may play a role in the plasma membrane and cytoskeleton that controls a cancer cell’s ability to survive ErSO-induced rapid cell swelling. Moreover, limited previous studies suggested FGD3 might activate Cdc42 and induce lamellipodium formation [24]. Cdc42 is activated by osmotic stress to regulate F-actin reorganization through its effector, the ARP2/3 complex [47, 48]. Therefore, we next investigated FGD3’s role as a potential Cdc42 GEF regulating F-actin reorganization under ErSO-induced cell swelling and osmotic stress. Using confocal microscopy, we showed that FGD3 overexpression induced lamellipodium formation at the cell border, while its knockout almost abolished lamellipodium formation (Fig. 4A left). Interestingly, in ErSO-treated MCF-7 and MCF-7-FGD3 OE cells, a significant patch-like F-actin aggregation and disruption of the internal stress fiber network were observed. In contrast, this reorganization was abolished in the FGD3 knockout cells, which helped ErSO-treated MCF-7-FGD3 KO cells maintain cell integrity (Fig. 4A Right). Since the ARP2/3 complex directly promotes lamellipodium formation, we next explored whether FGD3 regulates Cdc42 and its downstream effector. In immunoprecipitation assays, although ErSO activated Cdc42 in all three cell lines, FGD3 knockout significantly decreased Cdc42 activity (Fig. 4B, C). We next assessed the activity of the ARP2/3 complex by counting bright puncta, which represent intracellular ARP2/3 complex aggregation [49]. Consistent with the elevated Cdc42 activity, ARP2/3 activity was also significantly increased by ErSO. MCF-7-FGD3 OE cells had higher basal ARP2/3 activity level, which was further enhanced by ErSO. However, the FGD3 knockout again attenuated ErSO’s ability to activate ARP2/3 (Fig. 4D, E). Interestingly, when we used individual inhibitors, ML141 to block Cdc42 or CK-666 to block ARP2/3 activity [50, 51], lamellipodium formation induced by FGD3 overexpression was reversed and more stress fibers were observed (Fig. S5E). Importantly, the inhibitors also completely reversed the F-actin reorganization induced by ErSO in MCF-7 and MCF-7-FGD3 OE cells; inhibitor treated cells appeared similar to FGD3 knockout cells (Fig. 4F). These data indicate that FGD3 regulates F-actin reorganization through the Cdc42-ARP2/3 axis during ErSO-induced cell swelling and osmotic stress. However, the relationship between this F-actin-regulating axis, ErSO-induced PMR, and cell death was unclear.

### The FGD3-Cdc42-ARP2/3 axis mediates PMR and necrotic cell death

**Fig. 5:**
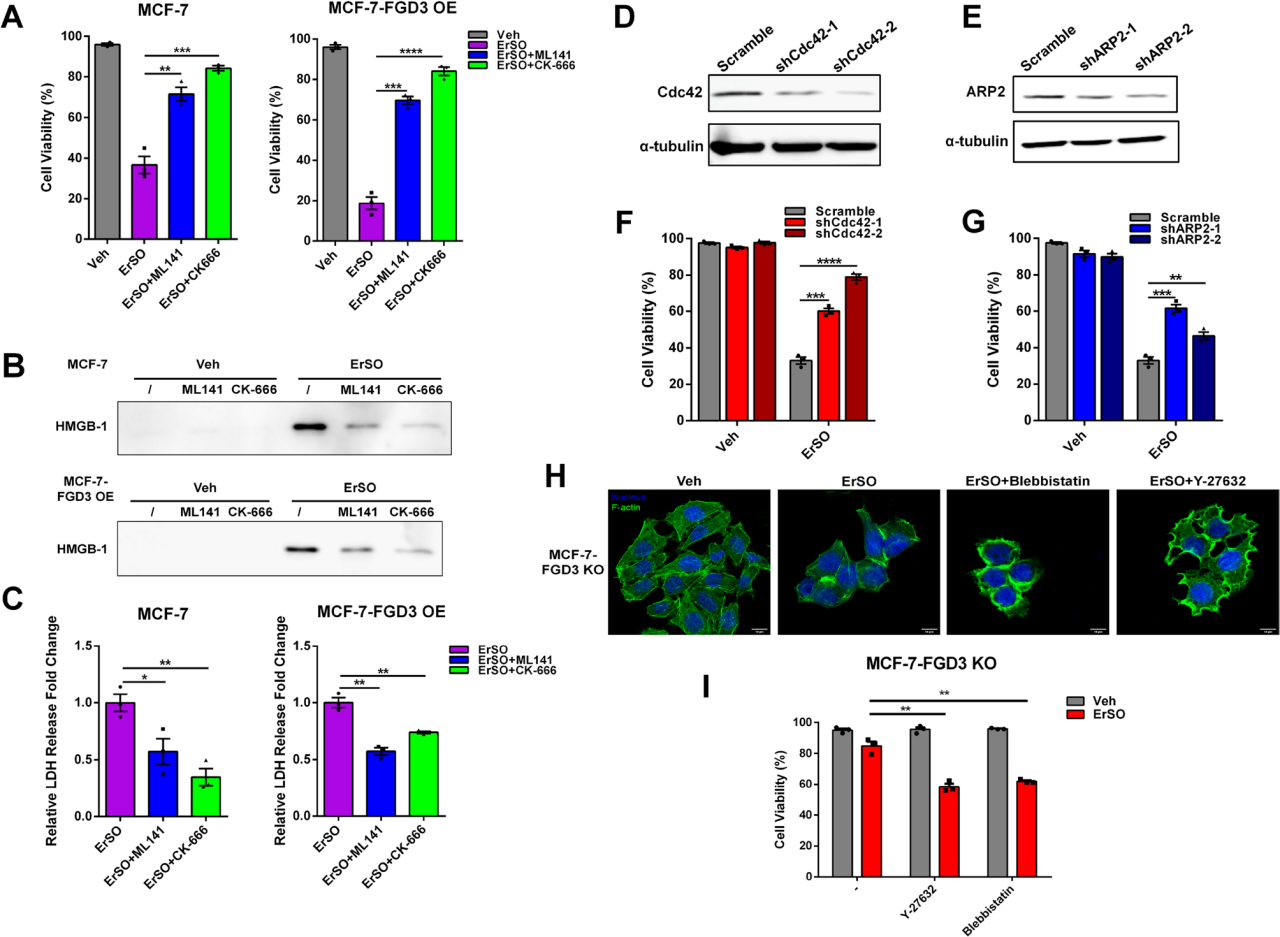
The FGD3-Cdc42-ARP2/3 axis mediates ErSO-induced PMR and necrosis. **A-C**, MCF-7 and MCF-7-FGD3 OE cells treated with vehicle, ErSO, ErSO + ML141 or ErSO + CK-666 for 24 h (ErSO, 100 nM; ML141, 10 µM; CK-666, 100 µM). **A**, Automated trypan blue exclusion assay comparing viability (*n* = 3). **B**, Western blot analysis of HMGB1 released into the medium. **C**, Relative fold change of LDH released into the medium (ErSO set to 1, *n* = 3). **D**,** E** Western blot analysis of **D**, Cdc42 and **E**, ARP2 levels after shRNA knockdown. **F**,** G**, Automated trypan blue exclusion assays comparing viability of scramble control and **F**, Cdc42 and **G**, ARP2 knockdown MCF-7 cells treated with 100 nM ErSO for 24 h (**F**,** G**, *n* = 3). **H**, Immunofluorescence images of MCF-7-FGD3 KO cells treated with Veh, ErSO, ErSO + blebbistatin, or ErSO + Y-27632 (ErSO, 1 µM; blebbistatin, 10 µM; Y-27632, 10 µM) for 1 h (scale bar 10 μm). **I**, Trypan blue exclusion assay comparing viability of MCF-7-FGD3 KO cells treated with Veh, ErSO, ErSO + blebbistatin, or ErSO + Y-27632 (ErSO, 100 nM; blebbistatin, 10 µM; Y-27632, 10 µM) for 24 h (*n* = 3). All data are mean ± s.e.m. *p < 0.05, **p < 0.01, ***p < 0.001, ****p < 0.0001, by Student’s t-test

To further investigate how this F-actin regulating axis relates to ErSO-induced PMR and necrosis, we cotreated the wild-type or FGD3 overexpressing MCF-7 and T47D cells with ErSO and either Cdc42 or ARP2/3 inhibitor. Notably, both inhibitors were about as effective as FGD3 knockout in blocking ErSO-induced cell death (Fig. 5A and Fig. S6A). Consistent with the inhibitors blocking ErSO-induced cell death, they also reduced HMGB1 and LDH release (Fig. 5B, C and Fig. S6B, C). This data indicates that Cdc42 and ARP2/3 act as downstream effectors of FGD3 to regulate PMR and associated necrotic cell death. To extend these studies, we performed shRNA knockdown of Cdc42 or ARP2 in the MCF-7 and T47D cells (Fig. 5D, E and Fig. S6D, E). Consistent with the effect of the inhibitors, Cdc42 or ARP2 knockdown significantly reduced ErSO-induced cell death (Fig. 5F, G and Fig. S6F).

These findings suggest lamellipodium formation regulated by the FGD3-Cdc42-ARP2/3 axis facilitates PMR, while the stress fiber network prevents rupture. To test this hypothesis, we disrupted stress fiber formation in the MCF-7-FGD3 KO cells using either a myosin II inhibitor, blebbistatin, or a ROCK inhibitor, Y-27632 [52–55]. Interestingly, in both control cells and in ErSO-treated MCF-7-FGD3 KO cells, the inhibitors abolished stress fiber formation (Fig. 5H and Fig. S7A). The loss of stress fibers was accompanied by significant formation of patch-like lamellipodium at the cell border. Moreover, cell viability assays showed that both inhibitors made the MCF-7-FGD3 KO cells more sensitive to ErSO killing (Fig. 5I). Taken together, we conclude that when breast cancer cells experience cell swelling and osmotic stress, FGD3 activates Cdc42 and then ARP2/3 to induce lamellipodium formation at the cell border and loss of the internal stress fiber network. This F-actin reorganization facilitates rupture of the plasma membrane. When FGD3 is absent, or the Cdc42-ARP2/3 axis is inhibited, the cells can maintain their internal stress fiber network and are much more likely to maintain cell integrity during cell swelling and become resistant to chemotherapy-induced PMR.

### FGD3 plays a broad role in mediating lytic cell death

**Fig. 6:**
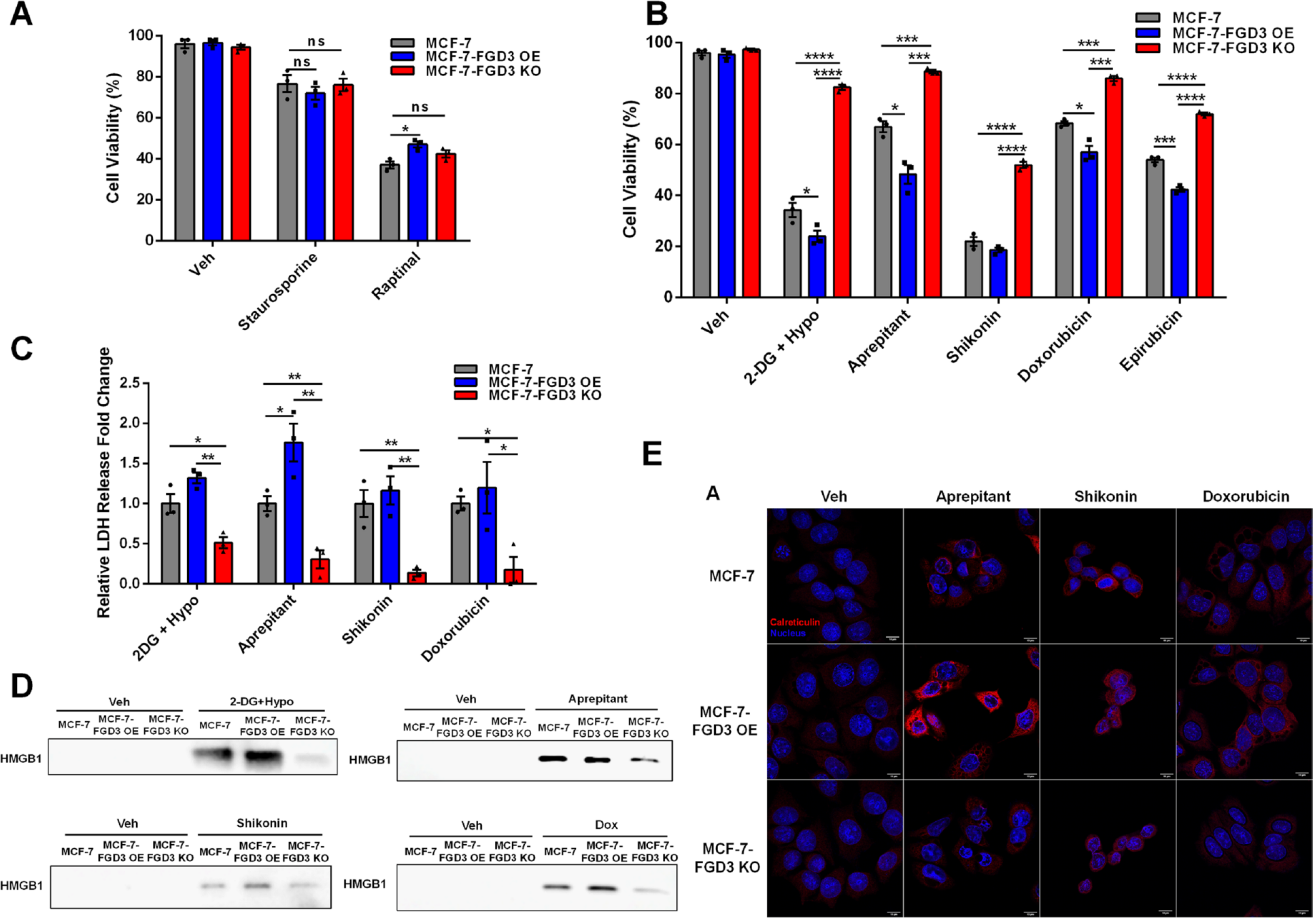
FGD3 plays a broad role in mediating PMR and lytic cell death. **A**, Automated trypan blue exclusion assay comparing the viability of MCF-7, MCF-7-FGD3 OE and MCF-7-FGD3 KO cells treated with vehicle, 100 nM staurosporine, or 5 µM Raptinal for 24 h (*n* = 3). **B-D**, MCF-7, MCF-7-FGD3 OE and MCF-7-FGD3 KO cells treated with inducers of necrosis (10 mM 2-deoxyglucose + hypotonic medium or 35 µM Aprepitant), necroptosis (1.5 µM Shikonin) or pyroptosis (50 µM Doxorubicin and its epimer, 40 µM Epirubicin) for 24 h. **B**, Automated trypan blue exclusion assay comparing cell viability (*n* = 3). **C**, Relative fold change of LDH released from the cells (MCF-7 set to 1, *n =* 3). **D**, Western blot analysis of HMGB1 released into the medium. **E**, Immunofluorescence images of calreticulin exposure on the surface of MCF-7, MCF-7-FGD3 OE and MCF-7-FGD3 KO cells treated with vehicle, 35 µM Aprepitant, 1.5 µM Shikonin or 50 µM Doxorubicin for 4 h. Red: calreticulin; Blue: nucleus (scale bar 10 μm). All data are mean ± s.e.m. *p < 0.05, **p < 0.01, ***p < 0.001, ****p < 0.0001, ns = not significant by Student’s t-test

Since plasma membrane rupture plays a pivotal role in three types of lytic cell death pathways, including necrosis, necroptosis and pyroptosis [3], we explored FGD3’s potential broad role in lytic cell death caused by diverse inducers. As expected, FGD3 level did not alter sensitivity to the apoptosis inducers staurosporine and Raptinal (Fig. 6A; [56, 57]). For necrosis, to mimic the effect of ErSO, we combined ATP depletion by 2-deoxy glucose and cell swelling induced by hypotonic medium [58]. The neurokinin receptor 1 (NK1R) antagonist aprepitant/Emend™, is an FDA-approved anti-nausea drug, now being explored as an anticancer agent [59–61]. Our laboratory showed that it kills breast cancer cells in a mixed necrotic cell death mode [16]. For regulated lytic cell death, the napthoquinone, Shikonin, induces necroptosis [62, 63]. Doxorubicin and its epimer, epirubicin, are widely used cancer chemotherapy drugs. Recently, doxorubicin was shown to induce pyroptosis through gasdermin E in breast cancer cells [29, 31]. For each lytic cell death inducer, we first confirmed it induced FGD3-independent ATP depletion and cell swelling (Fig. S8A-H). Notably, FGD3 knockout significantly reduced cell death induced by doxorubicin, epirubicin and the other treatments by preventing PMR, as shown by a decrease in release of HMGB1 and LDH from the FGD3 knockout cells (Fig. 6B-D). Conversely, FGD3 overexpression increased cell membrane disruption and the cancer cell’s sensitivity to lytic cell death induced by doxorubicin, epirubicin and the other death inducers. Moreover, calreticulin exposure elicited by these diverse death inducers was almost abolished by FGD3 knockout and strongly increased by FGD3 overexpression (Fig. 6E). Taken together, these data indicate that FGD3 mediates sensitivity to PMR across a broad range of lytic cell death pathways elicited by cell swelling-inducing anticancer therapies.

### FGD3 level is highly correlated with sensitivity to doxorubicin and ErSO in patient-derived organoid models

**Fig. 7:**
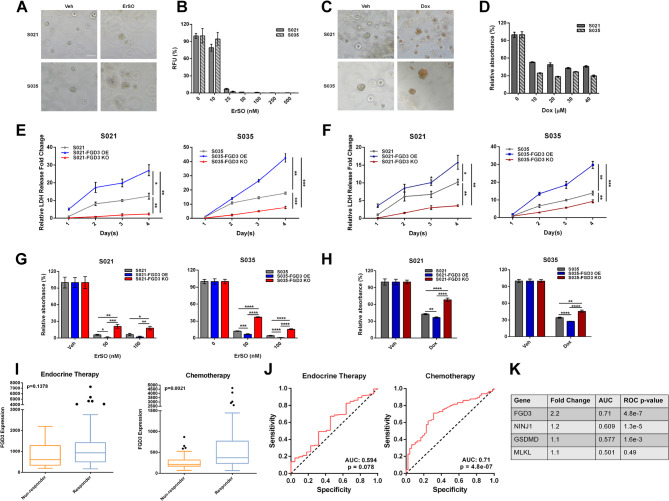
The level of FGD3 is highly correlated with sensitivity to anticancer therapies in 3D patient-derived organoids. **A**, Bright field image of the PDOs treated with vehicle or 100 nM ErSO for 24 h (scale bar 100 μm). **B**, 7-day alamarBlue cell viability assay of the PDOs treated with indicated concentrations of ErSO (*n* = 8). **C**, Bright field image of PDOs treated with vehicle or 40 µM Doxorubicin for 24 h (scale bar 100 μm). **D**, 7-day MTS cell viability assay of PDOs treated with the indicated concentrations of doxorubicin (*n* = 6). **E**,** F**, Relative fold change of LDH released into the medium from FGD3 OE or FGD3 KO organoids compared to LDH released into the medium from parental organoids during 1–4 days of treatment with **E**, 100 nM ErSO or **F**, 40 µM Doxorubicin (**E**,** F**: Day 1 parental organoids set to 1, *n* = 3). **G**,** H**, 7-day MTS assay comparing the relative viability of parental organoids to FGD3 OE or FGD3 KO organoids treated with **G**, 50 nM or 100 nM ErSO or **H**, 40 µM Doxorubicin (**G**,** H**: for each organoid model, vehicle was set to 100%; *n* = 6). **I**, Box plots showing higher FGD3 expression level in the relapse-free survival breast cancer patients receiving chemotherapies but not endocrine therapies. The dataset is from ROC Plotter. **J**, ROC analysis indicating FGD3 level is a top-quality biomarker (AUC > 0.7) in breast cancer patients receiving chemotherapies but not endocrine therapies. The dataset is from ROC Plotter. **K**, ROC analysis of FGD3, MLKL, GSDMD and NINJ1 in RFS breast cancer patients receiving chemotherapies. All data are mean ± s.e.m. *p < 0.05, **p < 0.01, ***p < 0.001, ****p < 0.0001, by Two-way ANOVA test (**E**,** F**) or Student’s t-test (**B**,** D**,**G**,** H**,**I**)

To explore the relevance of our findings in models that were more clinically relevant, we next investigated the role of FGD3 in the response to doxorubicin and ErSO in two breast cancer patient-derived organoids (PDOs), S021 and S035. These PDOs histologically exhibit trace ERα and contain FGD3 and TRPM4 (Fig. S9A). Both organoids responded to ErSO and doxorubicin, and these drugs elicited a necrotic cell death morphology (Fig. 7A-D). Although technical challenges have largely blocked analysis of rapid signaling pathways in PDOs, we were able to demonstrate rapid a-UPR activation (p-eIF2α and spliced-XBP1), protein synthesis inhibition and ATP depletion, in the PDOs (Fig. S9B-E). We then overexpressed or knocked out FGD3 in the PDOs (Fig. S9F). Importantly, FGD3 knockout significantly decreased LDH release from the organoids treated with either ErSO or doxorubicin, while FGD3 overexpression significantly increased LDH release from the PDOs (Fig. 7E, F). MTS assays were consistent with the LDH release data, with increased survival in the FGD3 knockout organoids and slightly less survival in FGD3 overexpression organoids (Fig. 7G, H). The impact of FGD3 levels on efficacy of widely used doxorubicin led us to explore the potential of FGD3 as a biomarker for identification of breast cancer patients most likely to benefit from chemotherapy. We performed a ROC Plotter database analysis of pre-treatment gene expression profiles and relapse-free survival (RFS) across all breast cancer patients who received any type of chemotherapy, including doxorubicin and other anthracyclines [64]. Importantly, FGD3 expression level is significantly higher in the responders than the non-responders (Fig. 7I). Notably, the ROC analysis using the same datasets indicated FGD3 is a strong predictive biomarker tightly correlated with increased relapse-free survival in breast cancer patients (FGD3 area under curve: AUC >0.7; Fig. 7J, K; AUC >0.7, strong biomarker; AUC >0.6, moderate biomarker; AUC < 0.6, weak, or not significant, biomarker; [64]). Despite their important roles in specific types of lytic cell death, at least in these unstratified breast cancer patients, NINJ1, GSDMD and MLKL showed much lower correlation with overall response to chemotherapy (Fig. 7K). As a control, in the database of relapse-free survival in breast cancer patients receiving endocrine therapies, which do not typically induce lytic cell death, there was not a statistically strong correlation between FGD3 level and patient response (Fig. 7I, J). Taken together, these data suggest that FGD3 facilitates the effectiveness of ICD-inducing chemotherapies that induce PMR. By increasing sensitivity to current anticancer therapies, high FGD3 levels may improve patient outcomes, explaining why it is a favorable prognostic marker.

## Discussion

**Fig. 8:**
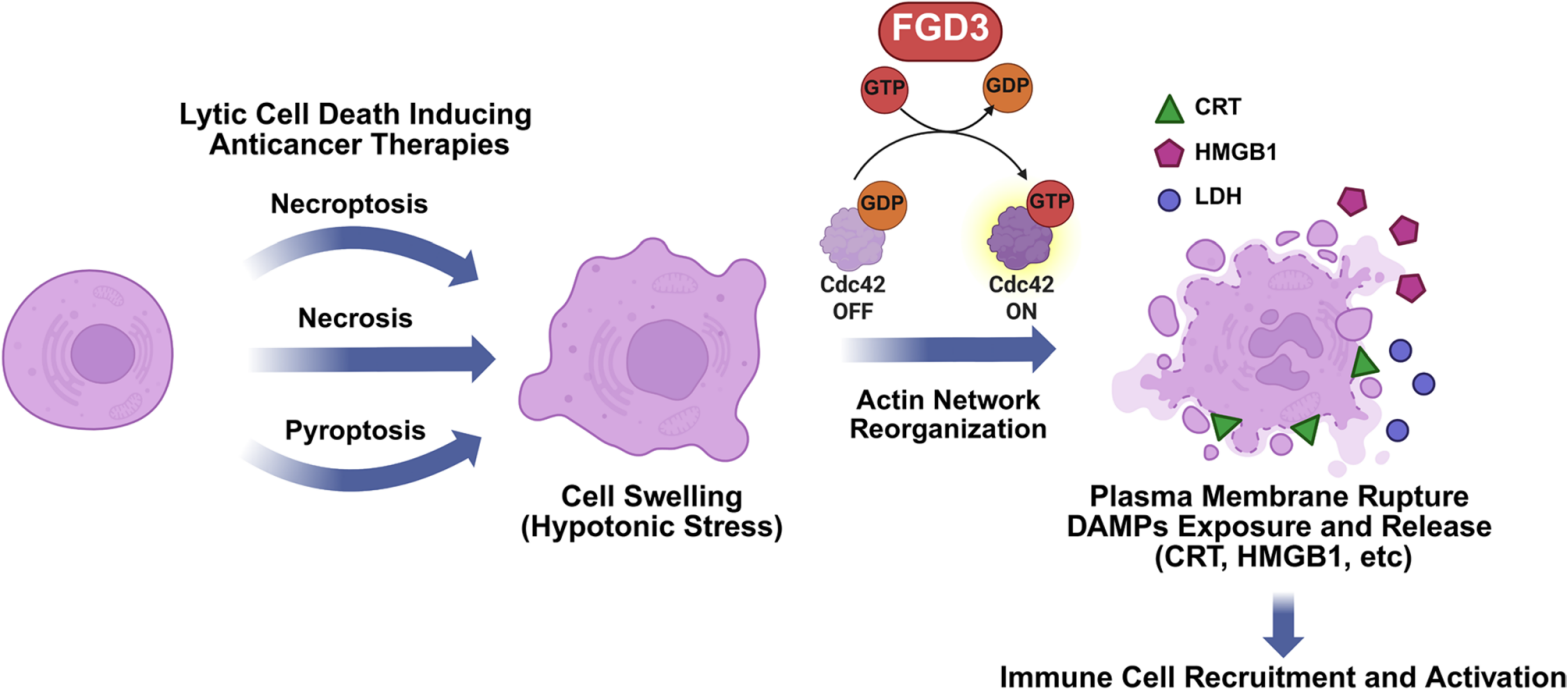
FGD3 regulates actin network reorganization, coupling cell swelling to lytic cell death induced by diverse anticancer therapies – this is followed by the exposure and release of DAMPs. Created in BioRender. Zhu, J. (2025) https://BioRender.com/m95r996

Using lytic cell death inducers as ICD-inducing therapies is attractive because they directly kill cancer cells and induce PMR, releasing DAMPs that activate immune cells - potentially enhancing immunotherapy [3, 4]. Recently, there has been intense focus on regulated pore-forming oligomers that directly disrupt membrane integrity – gasdermins and MLKL in the initial phase of pyroptosis and necroptosis, respectively [8, 65, 66]. Using studies primarily in mouse immune cells undergoing pyroptosis, NINJ1 emerged as playing a pivotal role in end-stage PMR, enabling the release of LDH and other large proteins [9]. Important structural studies have provided insight into how NINJ1 oligomerizes, with two proposed models for how it induces PMR [10, 11]. PMR in necrosis that is induced by cell swelling, independent of pore-forming oligomers is less well studied. For necrosis that involves cell swelling and ATP depletion, our anticancer drug ErSO and ErSO-related compounds induce cell swelling by opening the calcium-activated, ATP-inhibited plasma membrane channel, TRPM4 [16, 46, 67]. However, proteins responsible for the next stage, commitment of the swollen and stressed cancer cells to PMR, remained obscure. From a genome-wide CRISPR screen with selection against ErSO, database analysis, ErSO-resistant mutants and studies in cells in culture, PDOs and mice, we identify FGD3 as a critical regulator for the decision of cancer cells undergoing cell swelling to proceed to PMR and lytic cell death (Fig. 8).

Unexpectedly, TRPM4, which was previously demonstrated by our laboratory to be important in the ErSO-induced necrosis, was not identified in this CRISPR screen. After exploring the raw reads of the sequencing, we found that the sgRNA sequences against TRPM4 were amplified, but showed a high variability between replicates, potentially explaining why it was not picked up by the algorithm. Interestingly, NINJ1 did not emerge in our CRISPR screen. This is likely because NINJ1 knockout macrophages treated with pyroptosis inducers exhibit a ballooned morphology and eventually die [9]. The 21-day CRISPR screen allows ample time for the swollen ErSO-treated breast cancer cells to undergo NINJ1-independent necrosis. Although FGD3 was identified in the genome-wide CRISPR-Cas9 screen against ErSO and was down-regulated in some ErSO-resistant clones, where FGD3 impacted ErSO-induced necrosis was unclear (Fig. S1A). FGD3 knockout or overexpression did not impact any of the early steps in the a-UPR or the subsequent cellular stresses induced by ErSO. Since FGD3 knockout strongly prevented the stressed cancer cells from ErSO-induced cell death in multiple contexts and FGD3 overexpression enhanced cell death, FGD3 level must impact the end-stage decision of whether stressed and swollen cancer cells proceed to PMR and lytic cell death. Supporting this, FGD3 level strongly impacts cell death in a simple model in which cell swelling is induced by hypotonic medium and ATP is depleted by 2-deoxyglucose.

The action of FGD3 was largely unstudied. FGD3’s effects on PMR are distinct from those of members of the gasdermin family and MLKL, which form pores on the plasma membrane to initiate PMR [65, 66]. RNA-seq of ErSO-resistant cells in which FGD3 level is downregulated, showed strong differential expression of genes related to the cytoskeleton, connecting FGD3’s effect to the cytoskeleton. There was limited information suggesting FGD3 was a GEF of Cdc42. We show FGD3 sensitizes cells to PMR by regulating actin reorganization through the Cdc42-ARP2/3 axis. Reportedly, Cdc42 can be activated by hypotonic stress to regulate the formation of membrane protrusions and submembrane patch-like F-actin structures [47]. However, how Cdc42 is activated and how such actin reorganization is related to cell death was not known. Our study demonstrates that FGD3 activates Cdc42 and its downstream effector, the ARP2/3 complex under ErSO-induced osmotic stress, inducing membrane ruffling and the formation of submembrane F-actin patches. This actin reorganization is accompanied by the disappearance of stress fibers inside the cells, which eventually facilitates PMR and cell death. FGD3 knockout or downregulation of the activity of Cdc42 or ARP2/3 significantly attenuated this actin reorganization, PMR, and cell death. Moreover, disrupting the RhoA-dependent formation of stress fibers facilitated ErSO-induced cell death. How such actin reorganization controls plasma membrane integrity is not fully understood. Here, we speculate that the lack of FGD3 suppresses the activity of Cdc42 and this potentially favors RhoA activity during cell swelling and osmotic stress [68]. RhoA and ROCK activity, which mediates stress fiber formation, is reported to be important for the rapid reassembly of actin cortex and retraction of membrane blebs to restore support of the plasma membrane by the actin network and prevents the membrane from rupturing [69–71]. Our findings couple the Rho GTPase-dependent actin reorganization to PMR and subsequent cell death. While we primarily focused on Cdc42’s role in our study, the effect of Rac and RhoA on PMR and necrotic cell death remain topics for future exploration.

Although FGD3 is highly expressed in breast cancer as a favorable prognostic marker, its function in the pathophysiology of breast cancer remains unclear [20–22]. The TCGA database analysis does not suggest FGD3 level is correlated with metastasis status and one previous study showed low FGD3 level might increase lymph node metastasis in a cohort of 60 young breast cancer patients [21]. Unexpectedly, we show that FGD3 knockout decreases migration and invasion in MCF-7 cells, which is consistent with its role in promoting lamellipodia formation. Therefore, FGD3’s role in the metastasis of different types of breast cancer remains controversial and requires future exploration. Our data provides a different perspective to potentially explain why FGD3 is a favorable prognostic marker in breast cancer. Notably, we demonstrate elevated FGD3 in breast cancer cells increases NK cell-mediated lysis. Moreover, FGD3 is a broad mediator of lytic cell death pathways, impacting PMR triggered by chemotherapy agents that induce necrosis, necroptosis and pyroptosis. The level of FGD3 strongly impacted whether cancer cells that undergo cell swelling and ATP depletion induced by several agents, FDA-approved aprepitant/Emend™ (necrosis inducer), shikonin (necroptosis inducer) and doxorubicin (pyroptosis inducer) proceed to PMR and cell death. While FDA-approved aprepitant has only recently entered clinical trials as a cancer therapeutic [72, 73], doxorubicin and epirubicin in various formulations are mainstays of breast cancer therapy. In standard cell culture and in more physiologically relevant 3D PDO models, we show FGD3 level correlates with PMR, and lytic cell death of organoids treated with ErSO or doxorubicin. Importantly, a broad ROC Plotter database analysis of RFS across breast cancer patients, unstratified by sub-type, and across all types of chemotherapy, including, but not limited to, doxorubicin and other anthracyclines, demonstrates FGD3’s role as a strong predictive biomarker for chemotherapy response. We therefore hypothesize that elevated FGD3 expression in breast cancers may be correlated with better patient outcomes because FGD3 both enhances NK cell-mediated cell lysis and increases the effectiveness of doxorubicin and other lytic cell death-inducing anticancer therapies. Although the correlation between NINJ1 level in breast cancer and chemotherapy response was much weaker than that of FGD3, it was nonetheless significant. Given that a role for NINJ1 in response to membrane stretch was identified very recently [74], this suggested that NINJ1 might impact the response to cell swelling induced by chemotherapy agents, extending the potential role of NINJ1 in PMR from inflammation in macrophage to breast cancer. The correlation between chemotherapy response and GSDMD level was very weak and marginal for MLKL. This may in part reflect their role in one type of lytic cell death, or the complex interplay of several factors that contribute to necroptosis and pyroptosis.

Since development of resistance to chemotherapy is nearly universal in the metastatic setting, in breast cancer patients with high FGD3 levels, chemotherapy is better able to induce lytic cell death. This leads to a smaller pool of surviving breast cancer cells in which acquired resistance can occur, potentially increasing the time to development of resistance. Tumor resistance to NK cell-mediated cytotoxicity due to down-regulation of ICAM-1 and other strategies is hindering the development of NK cell-based immunotherapy [75]. NK cell-mediated killing involves lytic cell death, which can be enhanced by the lamellipodia formation on the cancer cells [76, 77]. By increasing lamellipodia, a high FGD3 level may therefore enhance NK cell-mediated lytic cell death and release of DAMPs, serving as a potential way to overcome resistance. A limitation of this work is our use of NK-92 cells. While these cells have found wide application in immunotherapy clinical trials, they will not completely replicate the properties of NK cells from breast cancer patients; therefore, development of a panel of primary NK cells, preferably isolated from peripheral blood of diverse breast cancer patients, would enable full future exploration of this important topic.

In necrosis induced by ErSO, or by the combination of osmotic stress induced by hypotonic medium and ATP depletion induced by 2-DG, FGD3 knockout leads to near complete inhibition of PMR and cell death. In contrast, in cells treated with inducers of other lytic cell death pathways, aprepitant, shikonin or doxorubicin, FGD3 knockout only partially rescues the cells. It is common for anticancer agents to elicit a mixed cell death phenotype, with contributions from apoptosis-related pathways, which are not impacted by FGD3 levels, and necrosis-related pathways. For example, doxorubicin induces caspase cleavage, which can induce apoptosis and pyroptosis, leading to a mixed cell death type [29, 30]. Moreover, PMR in necroptosis and pyroptosis is initiated by pore-forming gasdermin and MLKL oligomers, and likely facilitated by NINJ1, which differs from what happens in necrosis. Therefore, in necroptosis or pyroptosis, FGD3 might only partially prevent the further rupture of the already disrupted and leaky plasma membrane.

FGD3-dependent lytic cell death induces release of prototypical DAMPs HMGB1 and ATP, which strongly attract human THP-1 monocytes. Extracellular calreticulin exposure on the membranes of stressed and dying cells increases engagement of NK cells, enhancing NK-mediated cytotoxicity [42–44]. FGD3 knockout nearly blocked HMGB1 release and calreticulin exposure induced by ErSO, aprepitant, shikonin and doxorubicin and FGD3 overexpression dramatically increased calreticulin exposure and HMGB1 release. This suggests FGD3’s regulation of PMR may impact the immunogenicity of anticancer therapies and highlights a potential role of FGD3 in enhancing antitumor immunity.

## Conclusion

FGD3, identified in a CRISPR screen, plays a pivotal role in actin reorganization controlling plasma membrane rupture and DAMPs release triggered by ErSO, doxorubicin and other chemotherapeutics inducing necrosis, necroptosis and pyroptosis. This highlights FGD3 as a new predictive biomarker potentially enhancing the efficacy and immunogenicity of lytic cell death-inducing chemotherapy and improving the efficacy of immunotherapy. With the strong correlation between FGD3 level and a favorable response to chemotherapy, FGD3 level can be used to identify breast cancer patients most likely to benefit from doxorubicin and other chemotherapy agents that induce lytic cell death.

## Supplementary Information

Supplementary Material 1.

Supplementary Material 2.

Supplementary Material 3.

Supplementary Material 4.

Supplementary Material 5.

Supplementary Material 6.

## Data Availability

No datasets were generated or analysed during the current study.

## Abbreviations

PMR: Plasma membrane rupture
DAMP: Damage-associated molecule pattern
FGD3: FYVE, RhoGEF and PH domain-containing protein 3
ICD: Immunogenic cell death
LDH: Lactate dehydrogenase
TCGA: The Cancer Genome Atlas
2D, 3D: 2-dimensional, 3-dimensional
PDO: Patient-derived organoid
OE: Overexpression
KO: Knockout
a-UPR: Anticipatory unfolded protein response
NK cell: Natural killer cell
AUC: Area under curve
RFS: Relapse-free survival
