# Supplementary material for: FGD3 mediates lytic cell death, enhancing efficacy and immunogenicity of chemotherapy agents in breast cancer
Source: J Exp Clin Cancer Res. 2025 Nov 13;44:299. doi: 10.1186/s13046-025-03559-5 (PMC12613929; doi:10.1186/s13046-025-03559-5)
Supplement: Supplementary file 5 — Supplementary Material 5. [file 13046_2025_3559_MOESM5_ESM.docx]

**Supplementary Information for**

**FGD3 Mediates Lytic Cell Death, Enhancing Efficacy and Immunogenicity of Chemotherapy Agents in Breast Cancer**

Junyao Zhu^1^, Xinyi Dai^1^, Santanu Ghosh^1^, Elaine Wei^1^, Chengjian Mao^1^, Qianjin Jiang^1^, Abigail J. Spaulding^1^, Michael P. Mulligan^1,2^, Roger Romero^4^, Yoo Jane Han^4^, Matthew W. Boudreau^1,2,3^, Olufunmilayo Olopade^4^, Paul J. Hergenrother^1,2,3^, David J. Shapiro^1,3,*^

^1^Departments of Biochemistry and Chemistry University of Illinois at Urbana-Champaign, Urbana, IL 61801, USA

^2^Carl R. Woese Institute for Genomic Biology University of Illinois at Urbana-Champaign, Urbana, IL 61801, USA

^3^Cancer Center at Illinois, University of Illinois at Urbana-Champaign, Urbana, IL 61801, USA

^4^Department of Medicine, The University of Chicago, Chicago, IL, 60637, USA

*Correspondence: David J. Shapiro Email: [djshapir@illinois.edu](mailto:djshapir@illinois.edu)

**Supplementary Materials:**

Supplementary Materials and Methods

Supplementary Figures S1-S9

Caption for Supplementary Movies S1-S3

**Supplementary Materials and Methods**

**Cell culture**

IGROV-1 cells were obtained from Dr. D. Kranz. PEO4 cells were obtained from Dr. S. Kaufmann. SK-OV-3 and ES-2 cells were obtained from ATCC. Before use in these studies, cell lines were verified by genotyping at the University of Arizona facility and were negative for Mycoplasma using a PCR assay. All cell lines were cultured at 37^°^C with 5% CO_2_ in phenol red-free medium [medium: IGROV-1, SK-OV-3 (RPMI 1640, 10% FBS), ES-2 (DMEM/F12, 10% FBS), PEO4 (DMEM, 10% FBS, 10 μg/mL insulin, 1:250 diluted Dulbecco NEAA, 1 mmol/L glutamine)].

**Supplementary Figures**

**
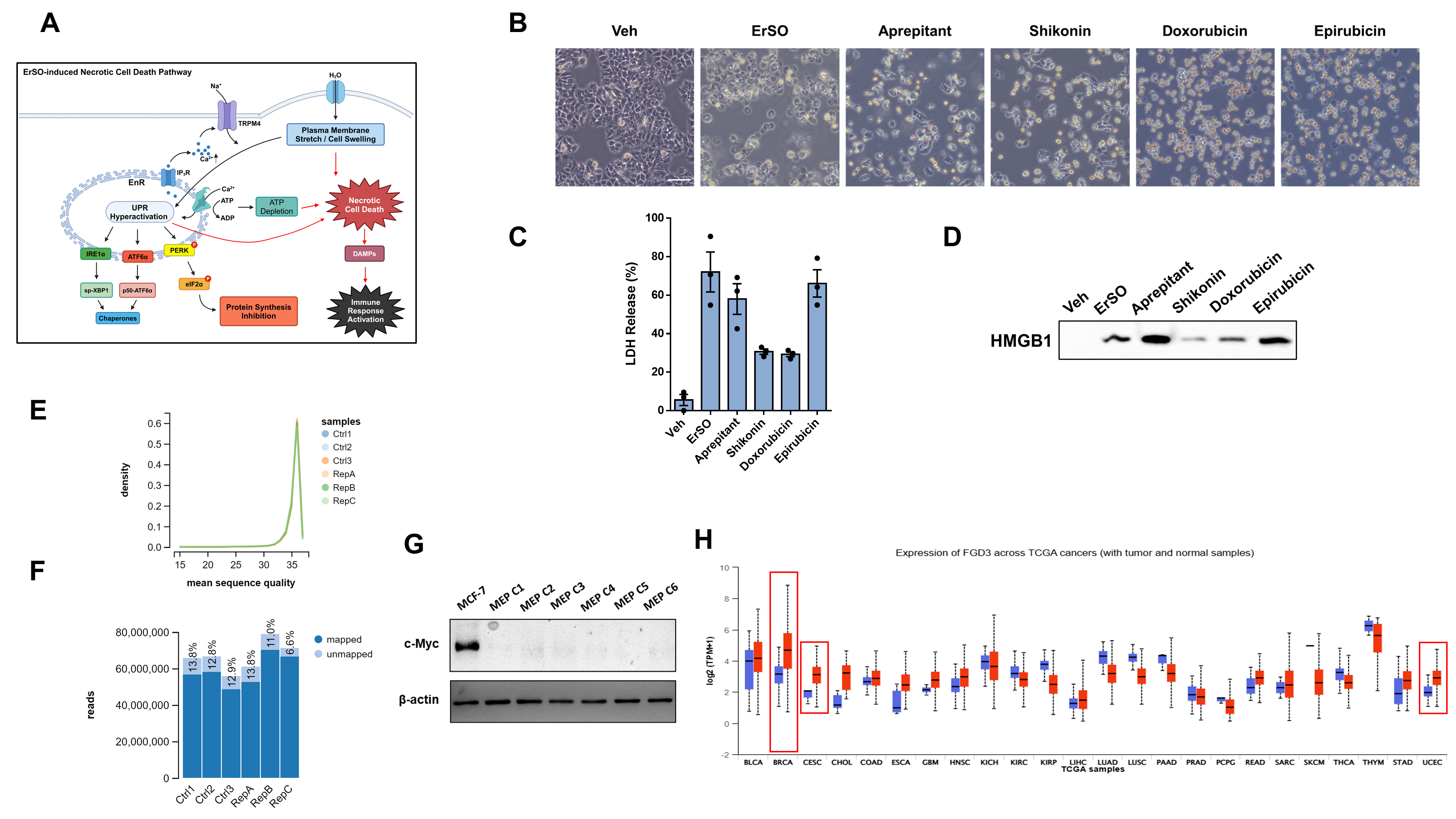
**

**Fig. S1** A CRISPR-Cas9 screen against ErSO identifies FGD3 as important in the necrosis pathway. **A,** Schematic model of ErSO action in breast cancer cells. ErSO induces efflux into the cell body of Ca^2+^ stored in the lumen of the endoplasmic reticulum, inducing a-UPR activation, protein synthesis inhibition, ATP depletion, cell swelling and eventually necrotic cell death followed by release of DAMPs. **B,C,D** MCF-7 cells were treated with Veh, 100 nM ErSO, 35 μM Aprepitant, 1.5 μM Shikonin, 50 μM Doxorubicin or 40 μM Epirubicin for 24 hours. **B,** Bright field images of the cells treated (scale bar 100 μm). **C,** The LDH release into the medium (*n = 3*). **D,** Western blot analysis of the HMGB1 released into the medium. **E,F** Details of the CRISPR screen showing it was of good quality. **E,** distribution of mean sequence quality, **F,** total number of reads and percentage of mapped reads. **G,** Western blot analysis of c-Myc level in MCF-7 cells and in the ErSO-persistent MCF-7 clones. **H,** Pan-cancer FGD3 expression profile compared with normal tissues according to the TCGA database. All data are mean ± s.e.m.

**
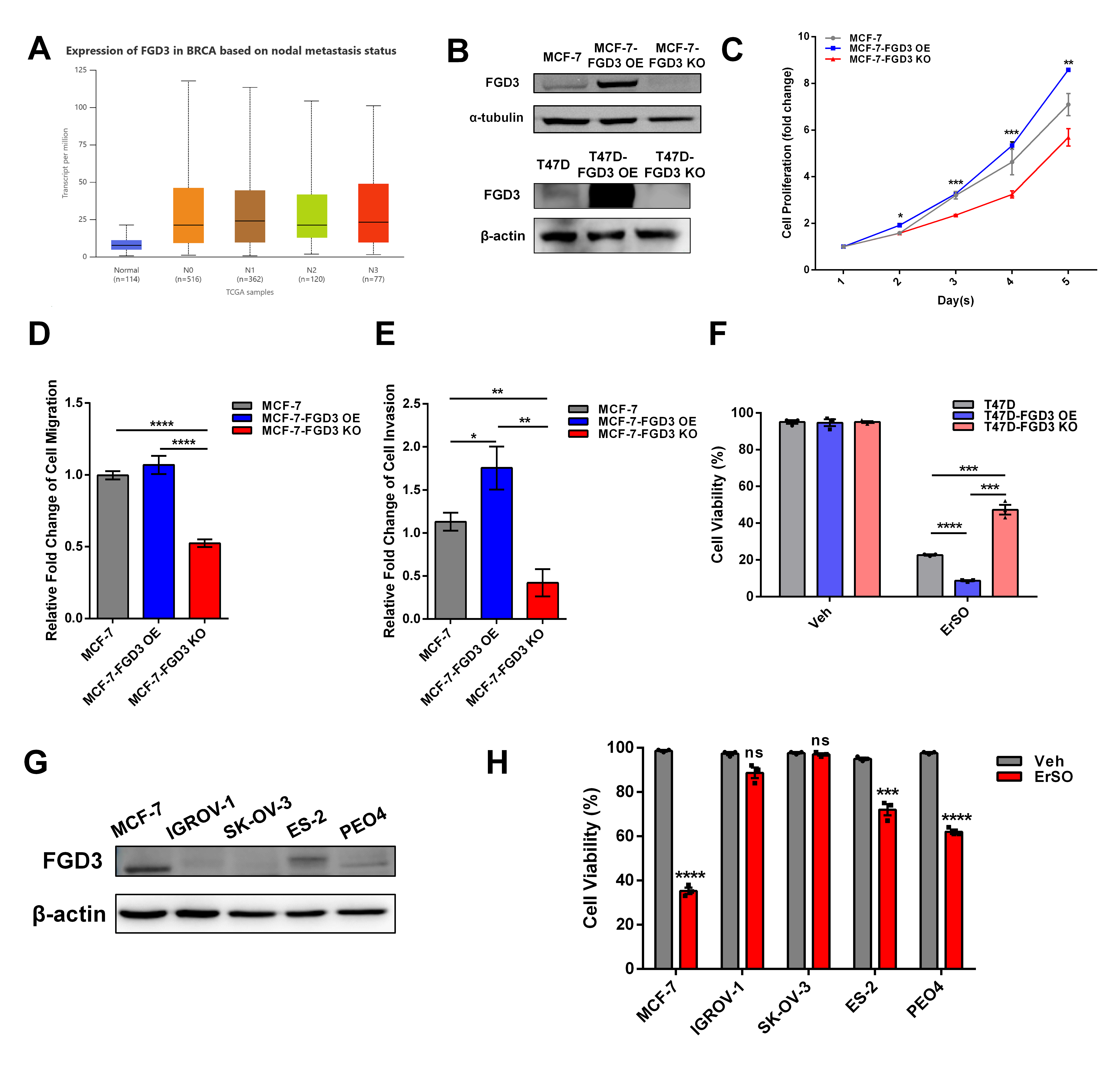
**

**Fig. S2** FGD3 level impacts cancer cell sensitivity to ErSO-induced cell death, cell migration and invasion with minimal effects on cell proliferation. **A,** FGD3 expression in normal tissues and tumors with different nodal metastasis status (N0-N3) according to the TCGA database. **B,** Western blot analysis of FGD3 level in **B, Top:** MCF-7, MCF-7-FGD3 OE and MCF-7-FGD3 KO cells; and **B, Bottom:** T47D, T47D-FGD3 OE and T47D-FGD3 KO cells. **C,** 5-day alamarBlue assay indicating a minimal change in cell proliferation between MCF-7, MCF-7-FGD3 OE and MCF-7-FGD3 KO cells (for each cell line day 0 set to 1; *n* = 3). **D,** 24-hour transwell cell migration assay comparing the relative fold change in migration of MCF-7, MCF-7-FGD3 OE and MCF-7-FGD3 KO cells through an uncoated membrane (*n* = 6). **E,** 24-hour transwell cell invasion assay comparing the relative fold change in cell invasion of MCF-7, MCF-7-FGD3 OE and MCF-7-FGD3 KO cells through a Matrigel-coated membrane (*n* = 6). **F,** Automated trypan blue exclusion assay comparing the viability of T47D, T47D-FGD3 OE and T47D-FGD3 KO cells treated with vehicle or 50 nM ErSO for 24 hours (*n* = 3). **G,** Western blot analysis of FGD3 level in MCF-7, IGROV-1, SK-OV-3, ES-2 and PEO4 cells. **H,** Automated trypan blue exclusion assay comparing the viability of MCF-7, IGROV-1, SK-OV-3, ES-2 and PEO4 cells treated with 100 nM ErSO for 24 hours (*n* = 3). All data are mean ± s.e.m. *p<0.05, **p<0.01, ***p<0.001, ****p<0.0001, ns = not significant by Student’s t-test.

**
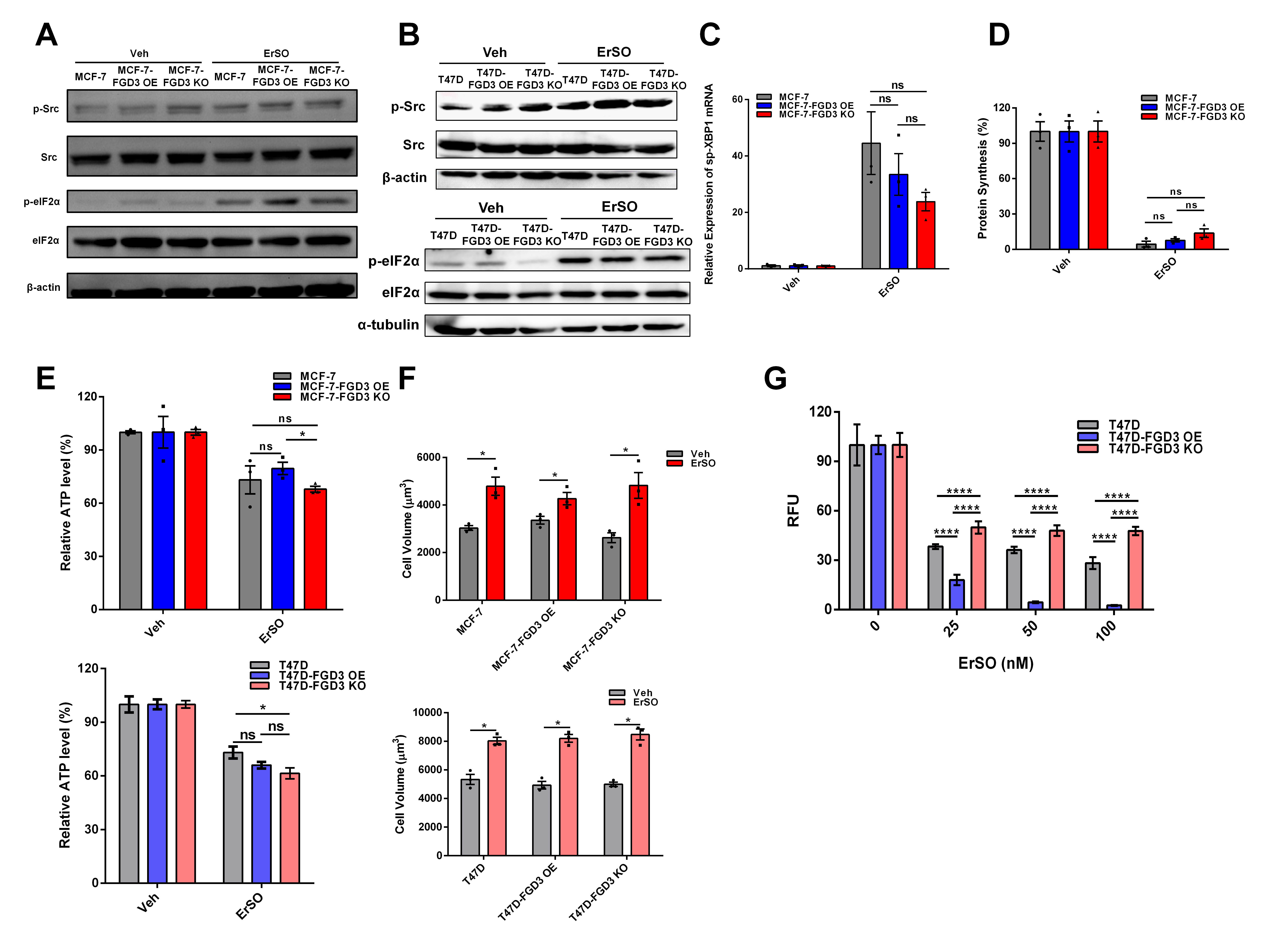
**

**Fig. S3** FGD3 does not regulate early steps in the ErSO-induced cell death pathway. (for most pathway details, see the condensed model in **Fig. S1A**) **A,B,** Western blot analysis of p-Src and p-eIF2α in **A,** MCF-7, MCF-7-FGD3 OE and MCF-7-FGD3 KO cells or **B,** T47D, T47D-FGD3 OE and T47D-FGD3 KO cells treated with vehicle or 1 μM ErSO for 30 min. **C,** qRT-PCR analysis of relative mRNA level of spliced XBP1 in MCF-7, MCF-7-FGD3 OE and MCF-7-FGD3 KO cells treated with vehicle or 200 nM ErSO for 2 hours (*n* = 3). **D,** Analysis of incorporation of ^35^S-methionine into protein; the relative percentage of protein synthesis in MCF-7, MCF-7-FGD3 OE and MCF-7-FGD3 KO cells treated with vehicle or 200 nM ErSO for 2 hours (for each cell line vehicle was set to 100%, *n* = 3). **E,** Relative ATP level in **E, Top:** MCF-7, MCF-7-FGD3 OE and MCF-7-FGD3 KO cells (*n* = 3); and **E, Bottom:** T47D, T47D-FGD3 OE and T47D-FGD3 KO cells treated with vehicle or 200 nM ErSO for 4 hours (*n* = 6). **F,** Cell volume of **F, Top:** MCF-7, MCF-7-FGD3 OE and MCF-7-FGD3 KO cells treated with vehicle or 1 μM ErSO for 1 hour; and **F, Bottom:** T47D, T47D-FGD3 OE and T47D-FGD3 KO cells treated with vehicle or 1 μM ErSO for 30 mins (*n* = 3). **G,** 4-day alamarBlue assay comparing the proliferation of T47D, T47D-FGD3 OE and T47D-FGD3 KO cells treated with the indicated concentrations of ErSO (*n* = 6). All data are mean ± s.e.m. *p<0.05, **p<0.01, ns = not significant by Student’s t-test.

**
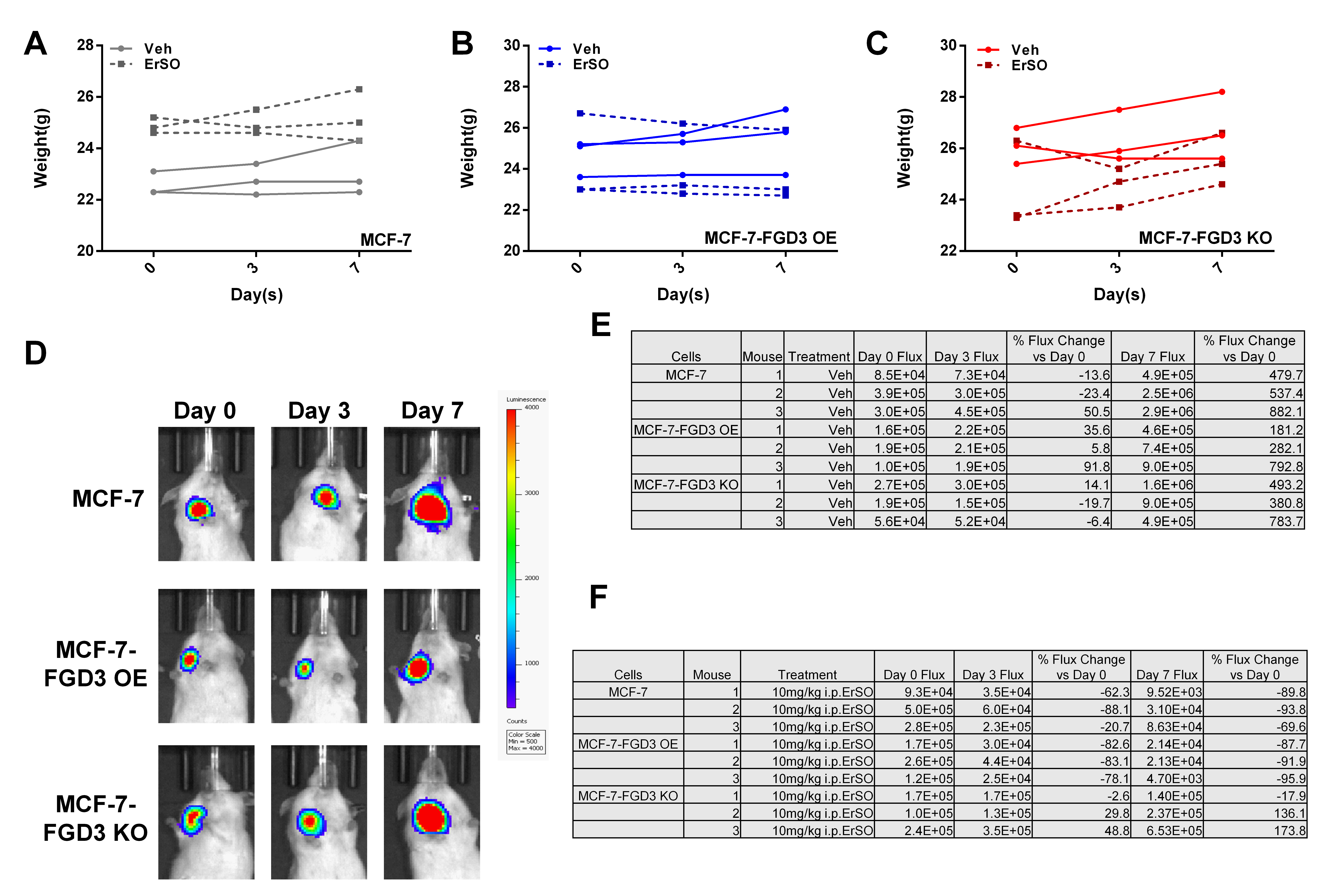
**

**Fig. S4** Orthotopic MCF-7, MCF-7-FGD3 OE and MCF-7-FGD3 KO tumors grow normally in mice. **A-C,** The weight of vehicle or ErSO-treated mice with: **A,** MCF-7 tumors, **B,** MCF-7-FGD3 OE tumor and **C,** MCF-7-FGD3 KO tumors. **D,** Representative BLI images showing the growth of MCF-7, MCF-7-FGD3 OE and MCF-7-FGD3 KO tumors in mice treated with vehicle. **E,F,** Tables showing the flux reading (photons/sec) and % flux change relative to Day 0 reading of mice: **E,** treated with vehicle or **F,** treated with 10 mg/kg ErSO i.p.

**
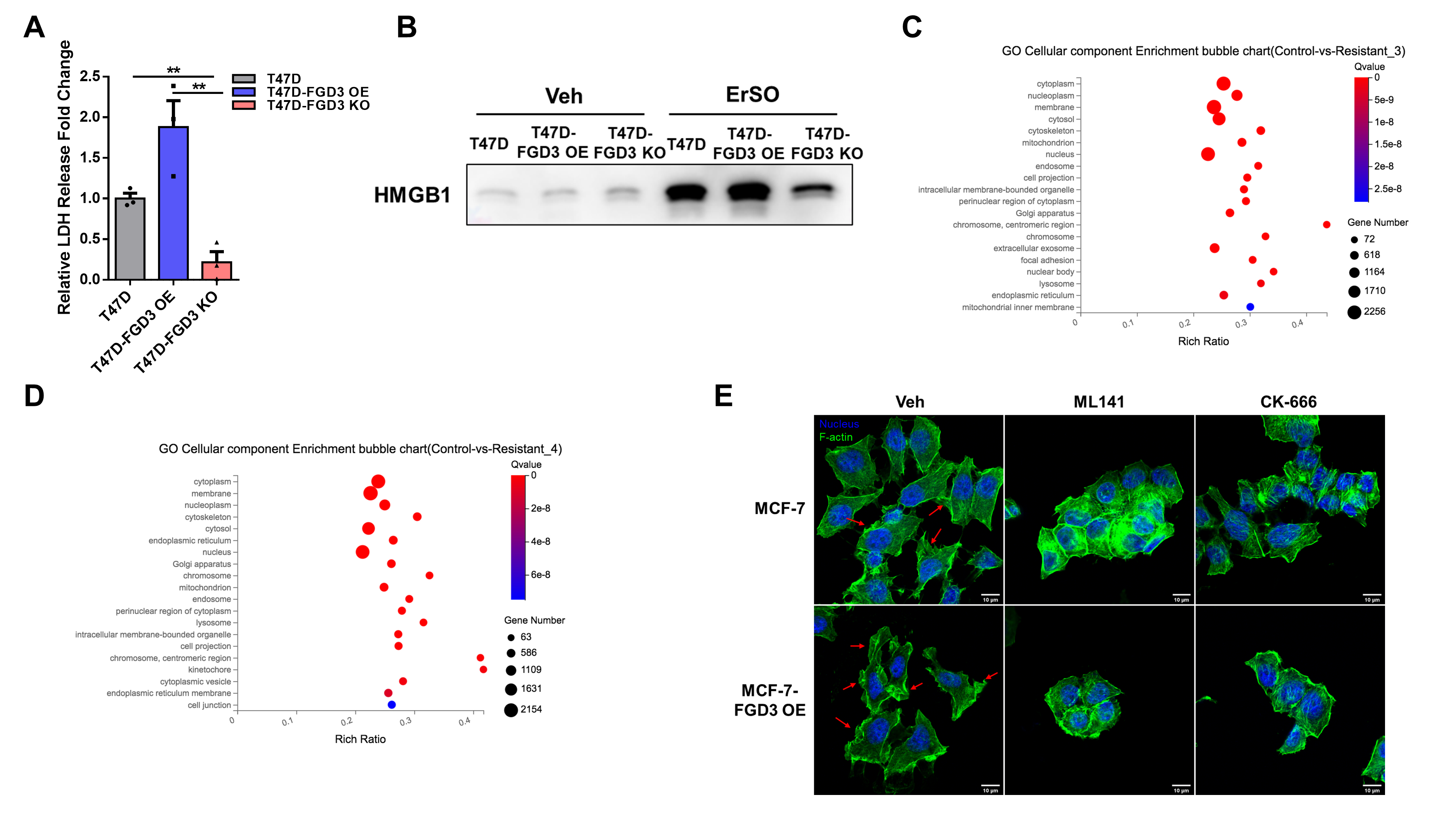
**

**Fig. S5** FGD3 plays a role in PMR and reorganization of F-actin through Cdc42 and ARP2/3. **A,** Relative fold change of LDH released into the medium from T47D, T47D-FGD3 OE and T47D-FGD3 KO cells treated with 50 nM ErSO for 24 hours (T47D cell LDH release set to 1, *n* = 3). **B,** Western blot analysis of HMGB1 released into the medium from T47D, T47D-FGD3 OE and T47D-FGD3 KO cells treated with 50 nM ErSO for 24 hours. **C,** GO cellular component enrichment analysis from RNA-seq data from ErSO-resistant MCF-7 clone 3. **D,** GO cellular component enrichment analysis from RNA-seq data from ErSO-resistant MCF-7 clone 4. **E,** Immunofluorescence images of MCF-7 or MCF-7-FGD3 OE cells treated with vehicle, 10 μM ML141 or 100 μM CK-666 for 24 hours. Green, F-actin; Blue, nucleus; Red arrows, lamellipodium (scale bar 10μm). All data are mean ± s.e.m. *p<0.05, **p<0.01, by Student’s t-test.

**
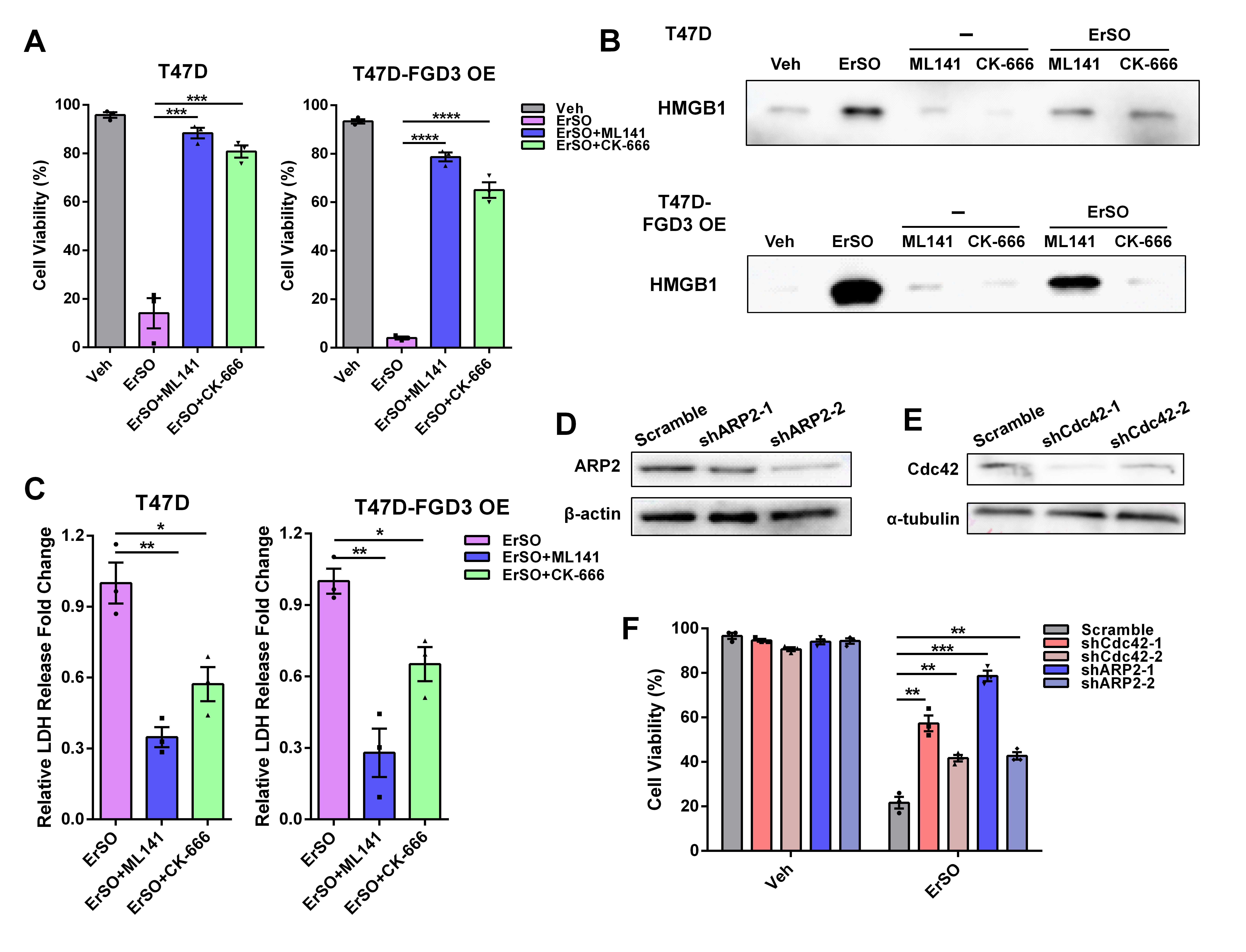
**

**Fig. S6** In T47D cells, the FGD3-Cdc42-ARP2/3 axis mediates ErSO-induced PMR and necrosis. **A-C,** T47D and T47D-FGD3 OE cells treated with vehicle, ErSO, ErSO + ML141 or ErSO + CK-666 for 24 hours (ErSO, 50 nM; ML141, 10 μM; CK-666, 100 μM). **A,** Automated trypan blue exclusion assay comparing viability (*n* = 3). **B,** Western blot analysis of HMGB1 released into the medium. **C,** Relative fold change of LDH released from the cells into the medium (ErSO set to 1, *n* = 3). **D,E,** Western blot analysis of **D,** ARP2 level. **E,** Cdc42 level after shRNA knockdown. **F,** Trypan blue exclusion assay comparing viability of scramble control, Cdc42 knockdown, or ARP2 knockdown T47D cells treated with 50 nM ErSO for 24 hours (*n* = 3). All data are mean ± s.e.m. *p<0.05, **p<0.01, ***p<0.001, ****p<0.0001, by Student’s t-test.

**
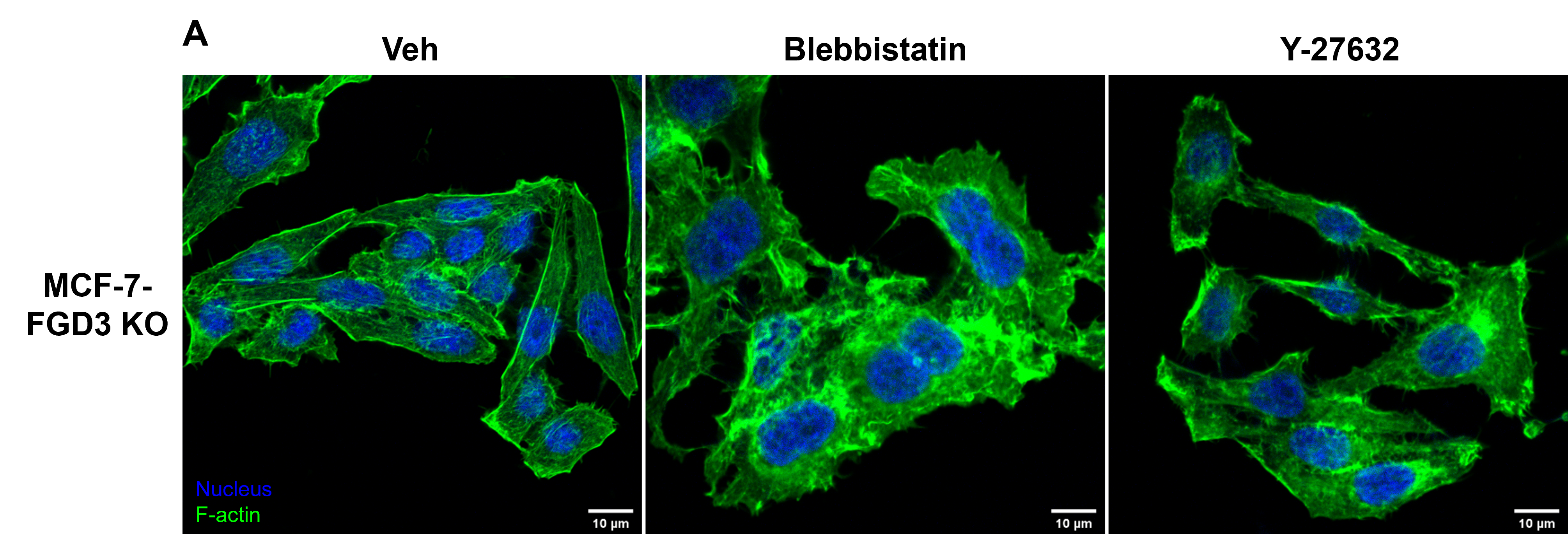
**

**Fig. S7** A Myosin II inhibitor or a ROCK inhibitor disrupt stress fibers in FGD3 knockout cells. **A,** Immunofluorescence image of MCF-7-FGD3 KO cells treated with vehicle, 10 μM blebbistatin or 10 μM Y-27632 for 24 hours. Green, F-actin; Blue, nucleus (scale bar 10μm).

**
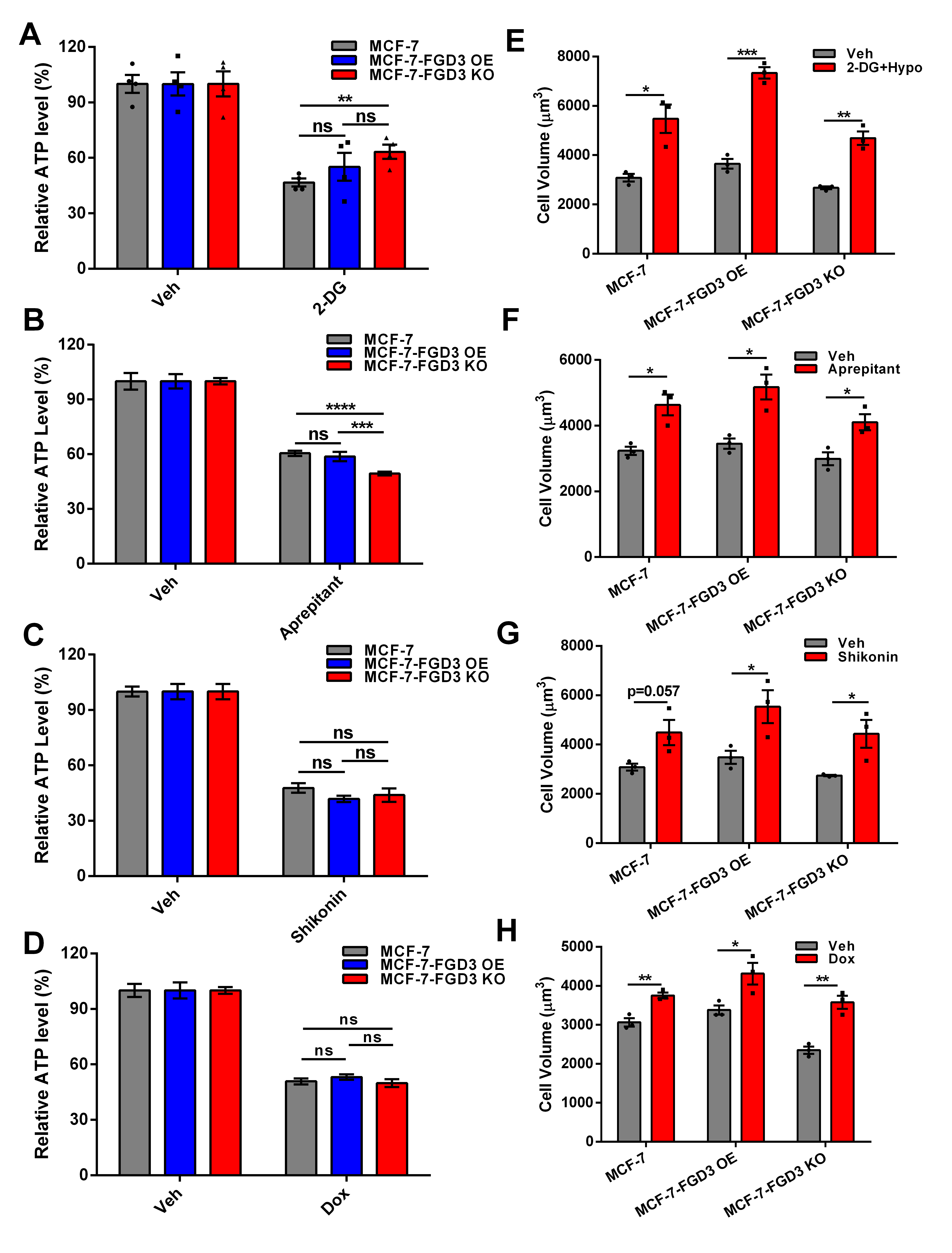
**

**Fig. S8** FGD3 does not regulate ATP depletion or cell swelling caused by diverse inducers of lytic cell death. **A-D,** Relative ATP level in MCF-7, MCF-7-FGD3 OE and MCF-7-FGD3 KO cells treated with vehicle or **A,** 10 mM 2-DG (2-deoxyglucose) for 2 hours (*n* = 3); **B,** 35 μM Aprepitant for 4 hours (*n* = 6); **C,** 1.5 μM Shikonin for 4 hours (*n* = 6); **D,** 50 μM Doxorubicin for 12 hours (*n* = 6). **E-H,** Cell volume of MCF-7, MCF-7-FGD3 OE and MCF-7-FGD3 KO cells treated with vehicle or **E,** 10 mM 2-DG + hypotonic medium for 1 hour; **F,** 35 μM Aprepitant for 2 hours; **G,** 1.5 μM Shikonin for 2 hours; **H,** 50 μM Doxorubicin for 4 hours (*n* = 3). All data are mean ± s.e.m. *p<0.05, **p<0.01, ***p<0.001, ****p<0.0001, ns = not significant by Student’s t-test.

**
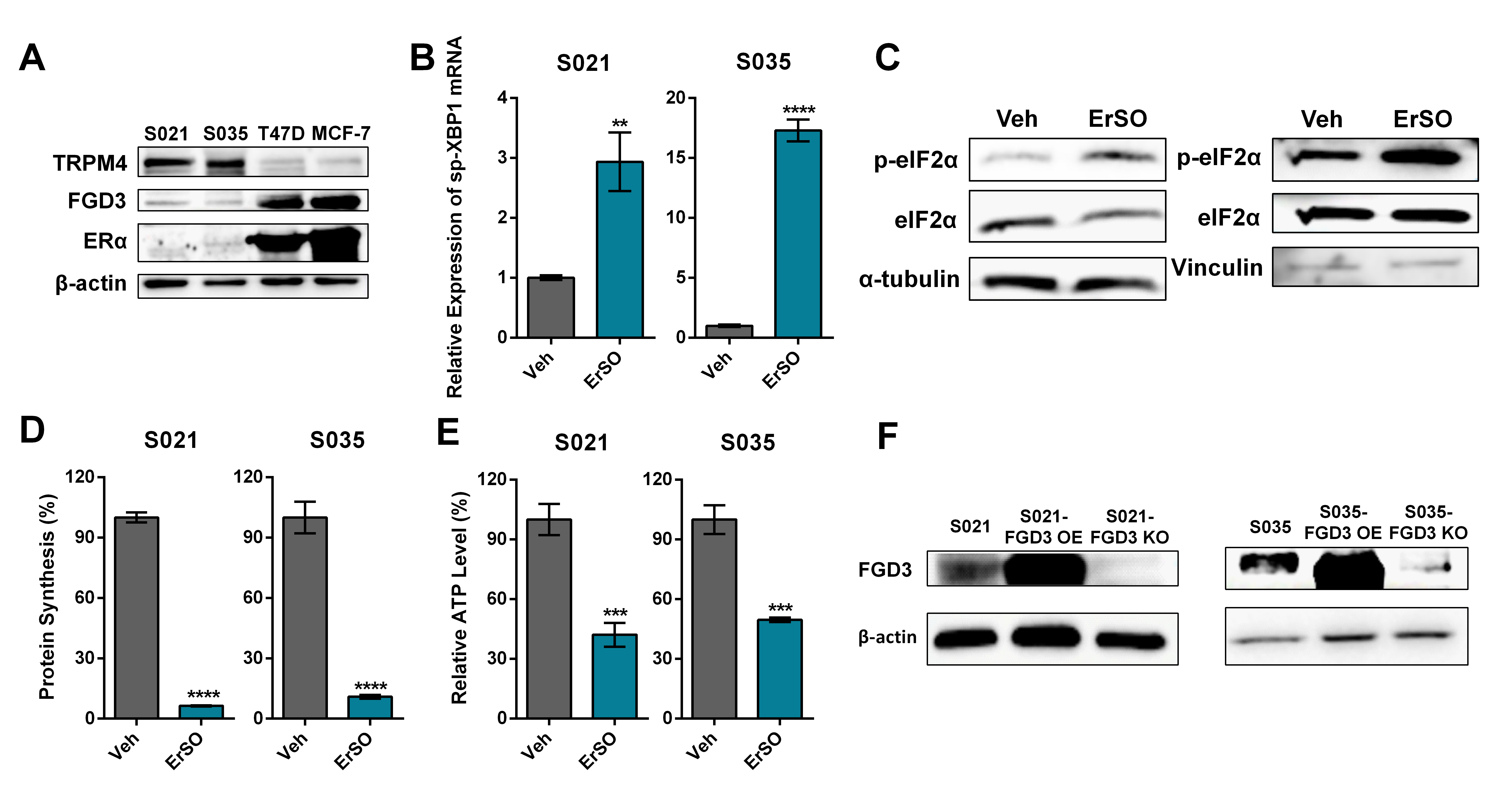
**

**Fig. S9** ErSO rapidly induces a-UPR in breast cancer PDOs. **A,** Western blot analysis of FGD3, ERα and TRPM4 level in 3D PDO models, and in standard culture T47D and MCF-7 cells. **B,** qRT-PCR analysis of relative mRNA level of spliced XBP1 in 3D PDO models treated with vehicle or 200 nM ErSO for 4 hours (*n* = 6). **C,** Western blot analysis of p-eIF2α in 3D PDO models treated with vehicle or 1 μM ErSO for 30 mins. **D,** Analysis of incorporation of ^35^S-methionine into protein indicating the relative percentage of protein synthesis in 3D PDO models treated with vehicle or 200 nM ErSO for 4 hours (vehicle set to 100%, *n* = 5). **E,** Relative ATP level in 3D PDO models treated with vehicle or 200 nM ErSO for 4 hours (vehicle set to 100%, *n* = 5). **F,** Western blot verification of FGD3 overexpression and knockout in 3D PDO models. All data are mean ± s.e.m. **p<0.01, ***p<0.001, ****p<0.0001 by Student’s t-test.

**Caption for Supplementary Movies S1-S3**

ErSO induces a rapid increase in intracellular calcium in MCF-7, MCF-7-FGD3 OE and MCF-7-FGD3 KO cells. MCF-7 (**S1**), MCF-7-FGD3 OE (**S2**) and MCF-7-FGD3 KO (**S3**) were loaded with 2.5 μM Fluo-4, AM for 1 hour. The cells were then treated with 10 μM ErSO and immediately placed in a micro cell incubator for live cell imaging.
