# Supplementary material for: FGD3 mediates lytic cell death, enhancing efficacy and immunogenicity of chemotherapy agents in breast cancer
Source: J Exp Clin Cancer Res. 2025 Nov 13;44:299. doi: 10.1186/s13046-025-03559-5 (PMC12613929; doi:10.1186/s13046-025-03559-5)
Supplement: Supplementary file 6 — Supplementary Material 6. [file 13046_2025_3559_MOESM6_ESM.docx]

**Uncropped Blots**


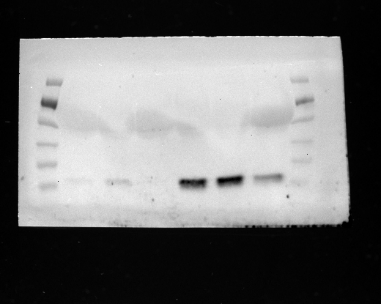


Fig. 3E

Fig. 4B


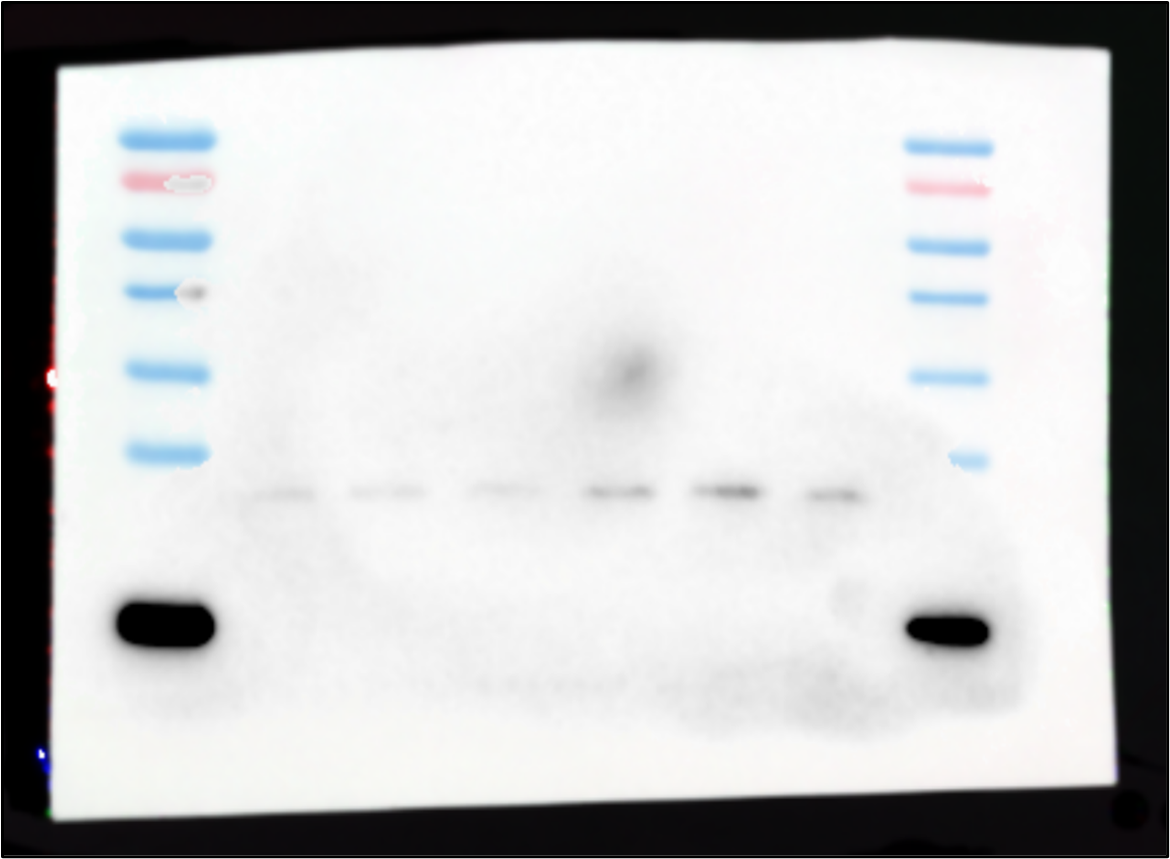

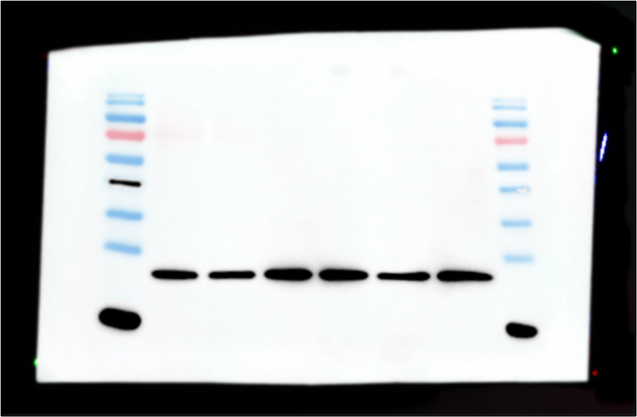


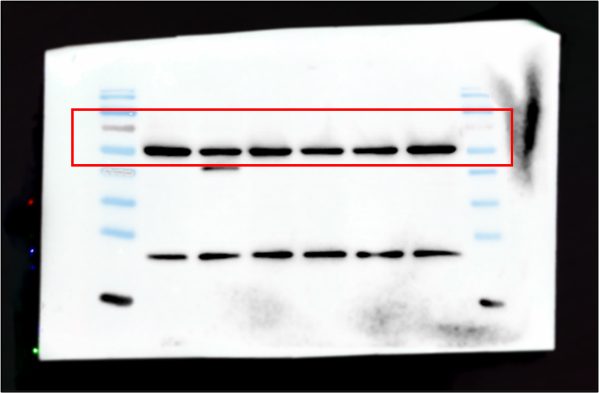


Fig. 5B


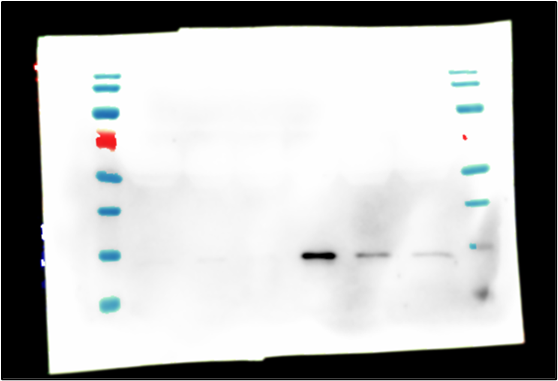

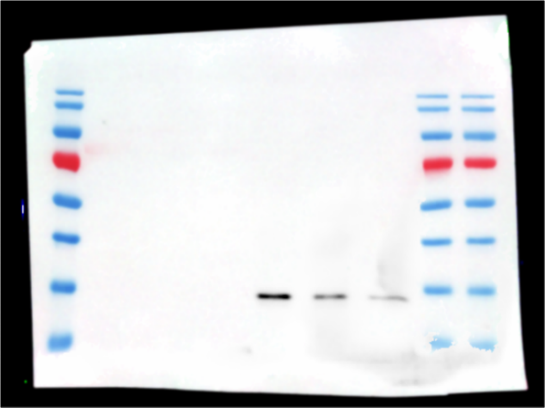


Fig. 5D


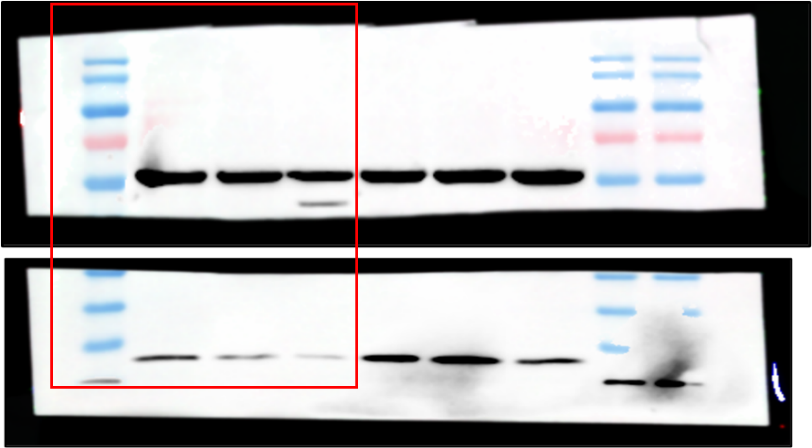


Fig. 5E


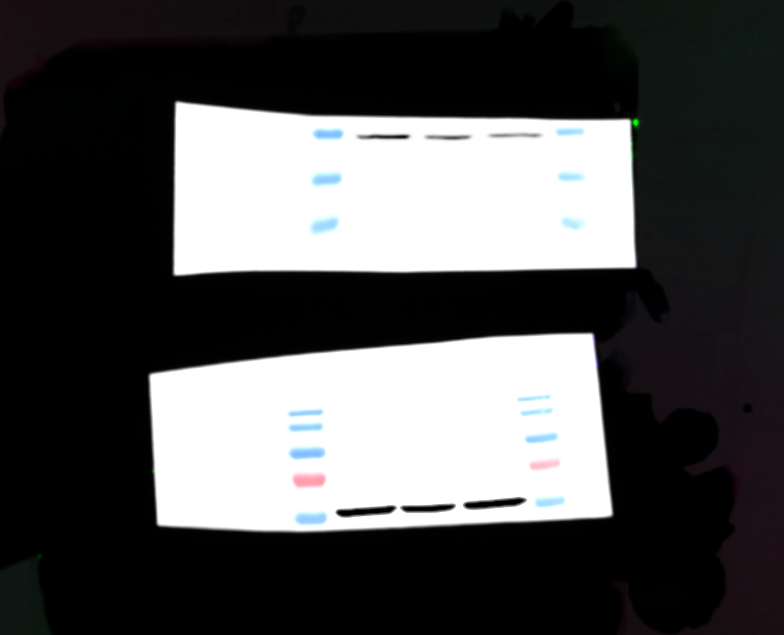


Fig. 6D

2DG + hypo

aprepitant


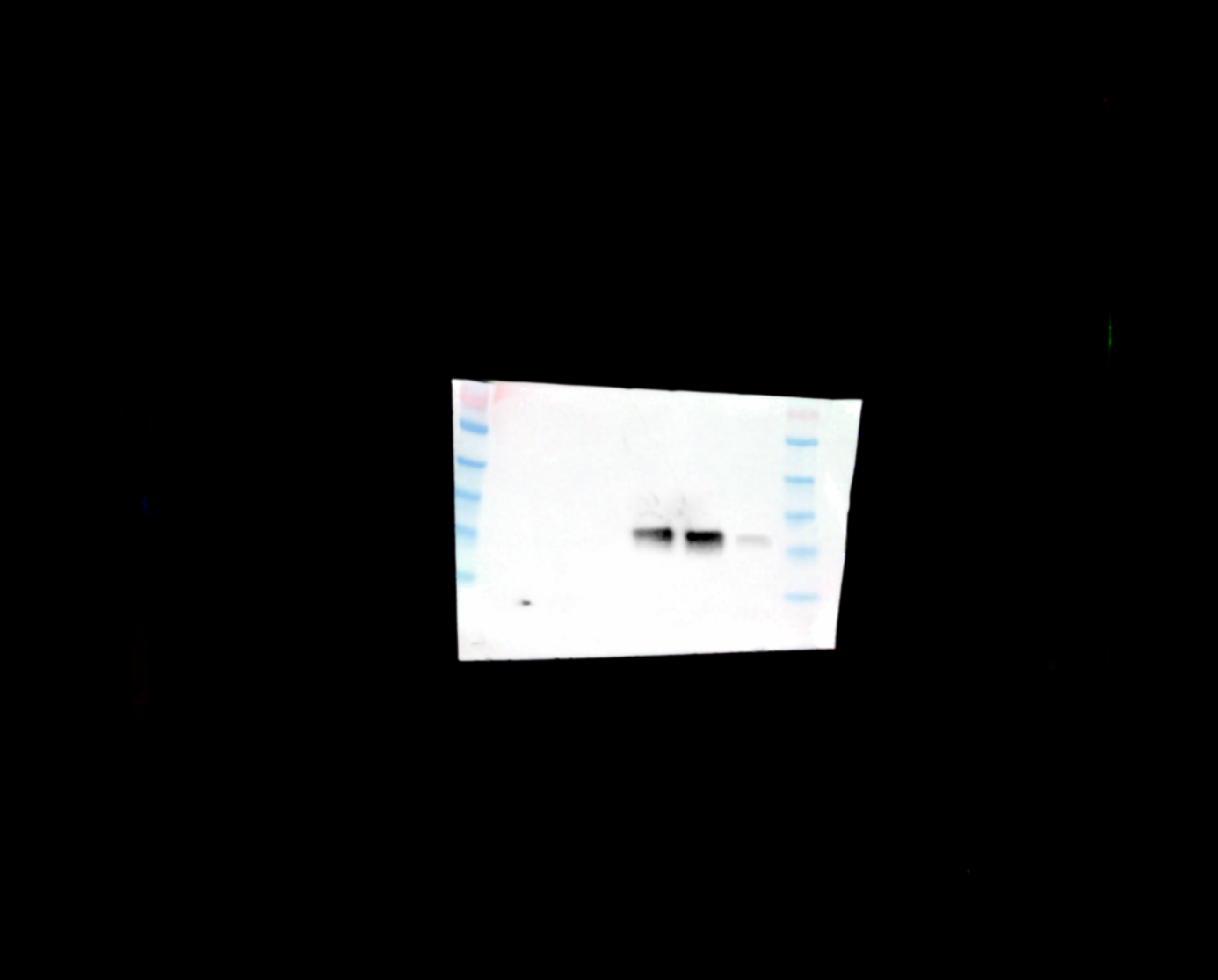

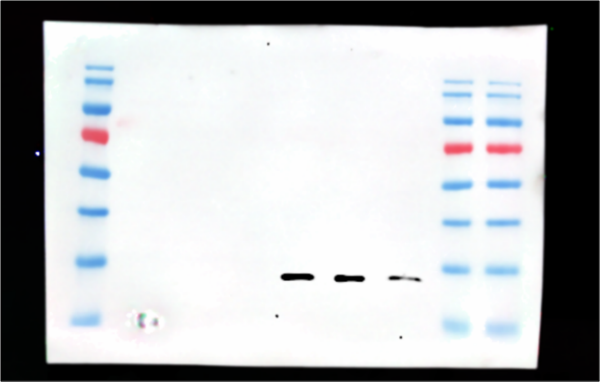


doxorubicin

shikonin


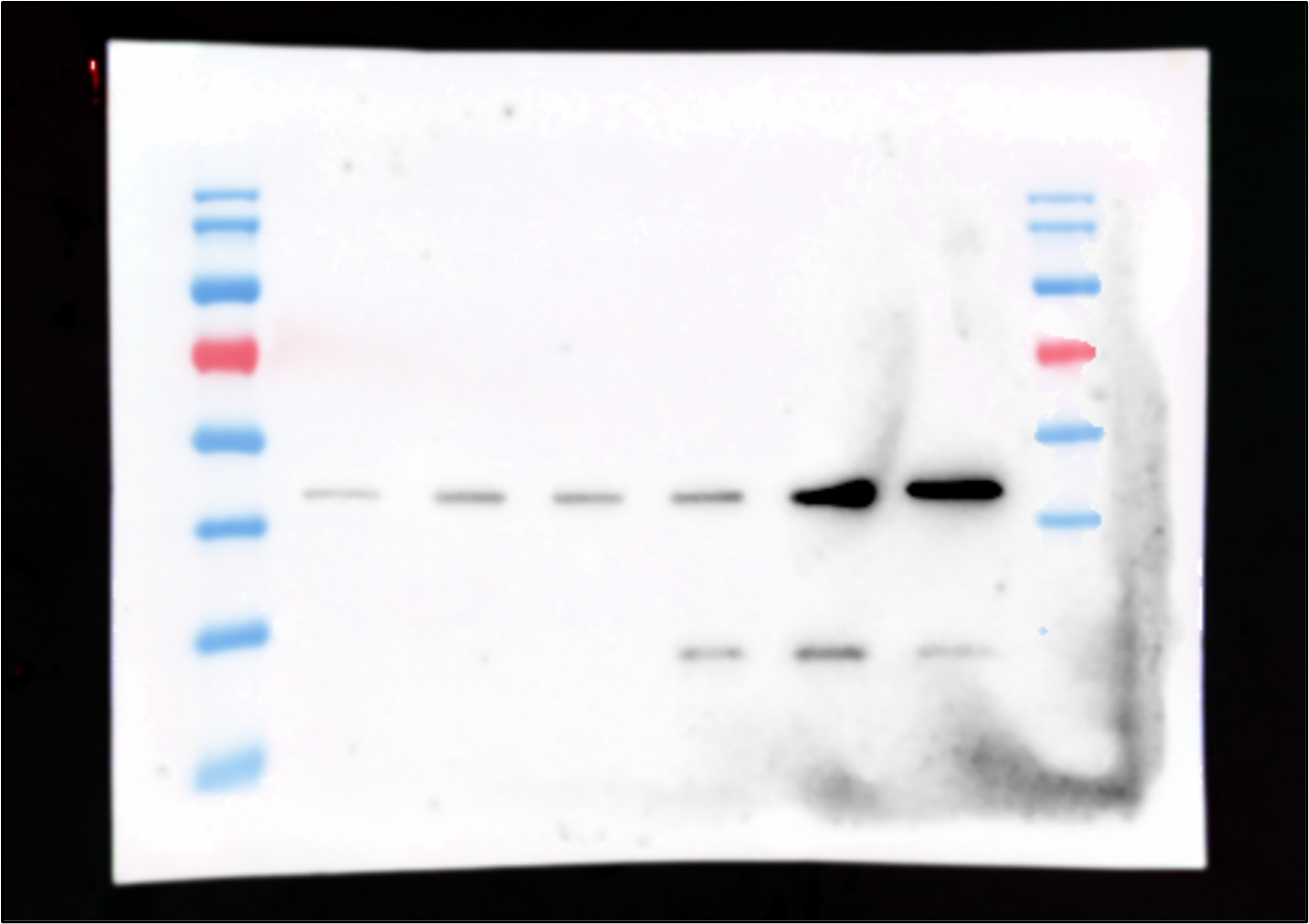

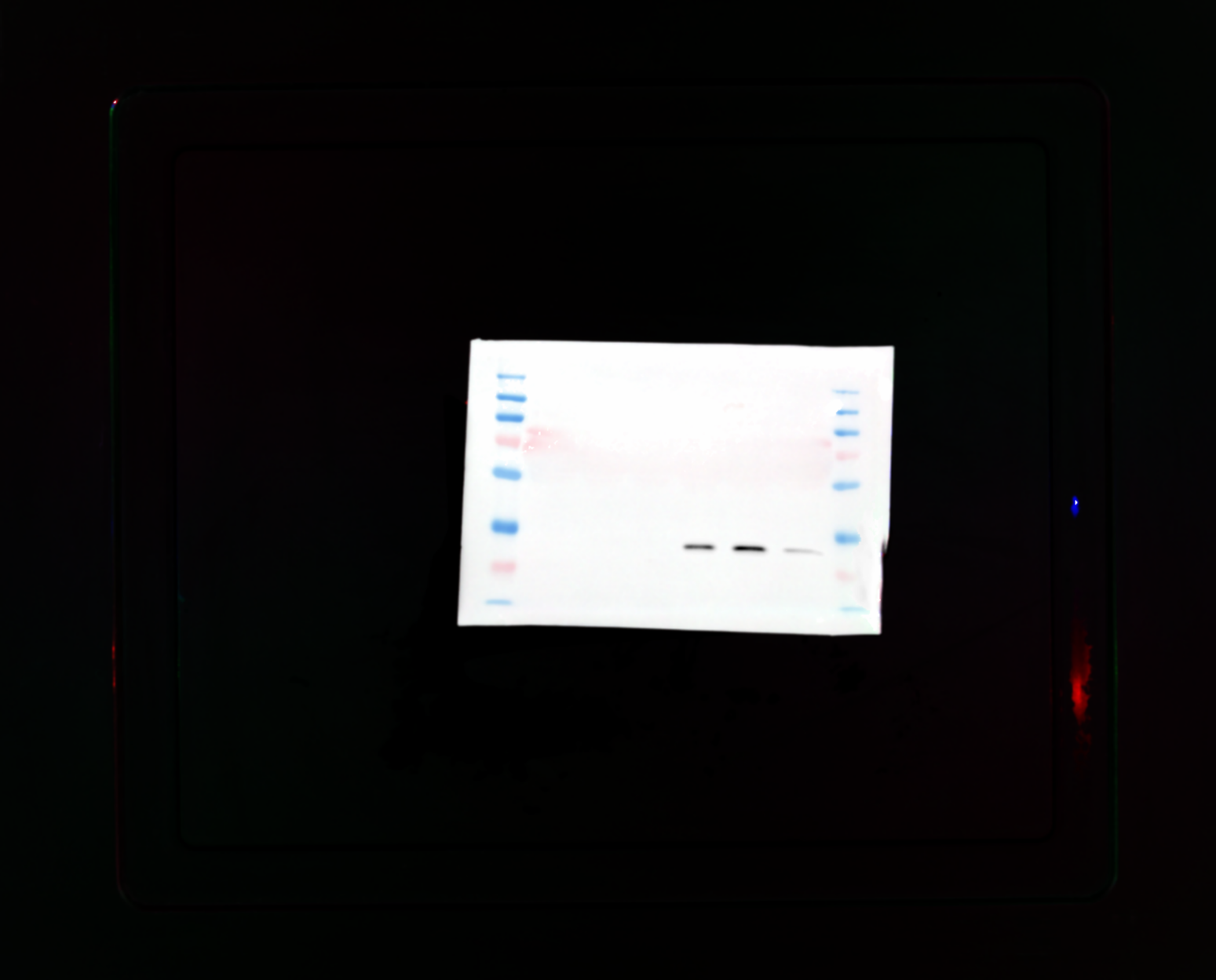


Fig. S1D


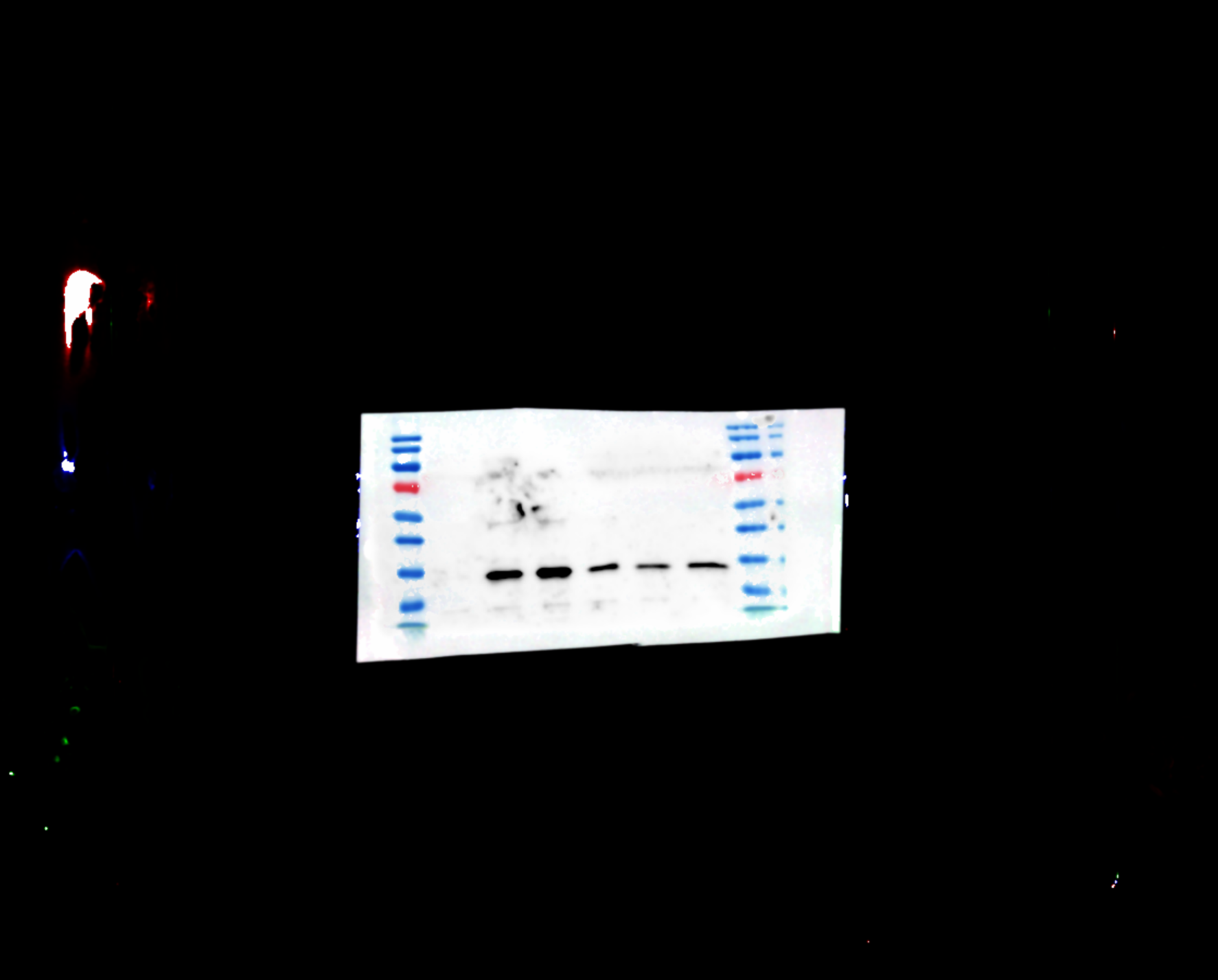


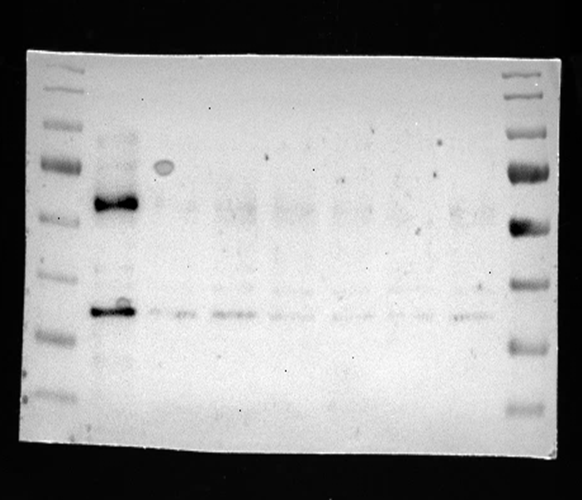

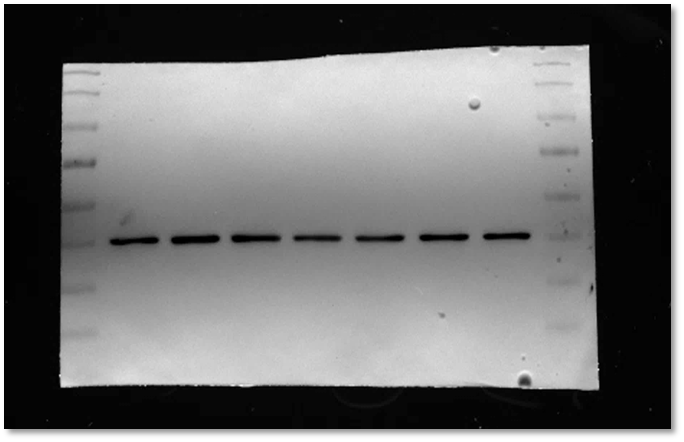


Fig. S1G

Fig. S2A


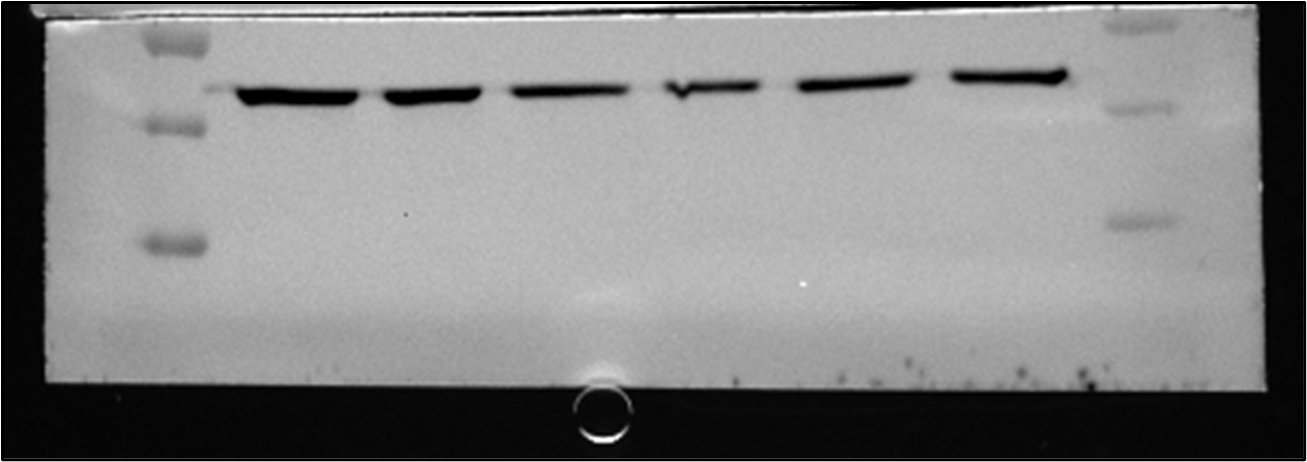

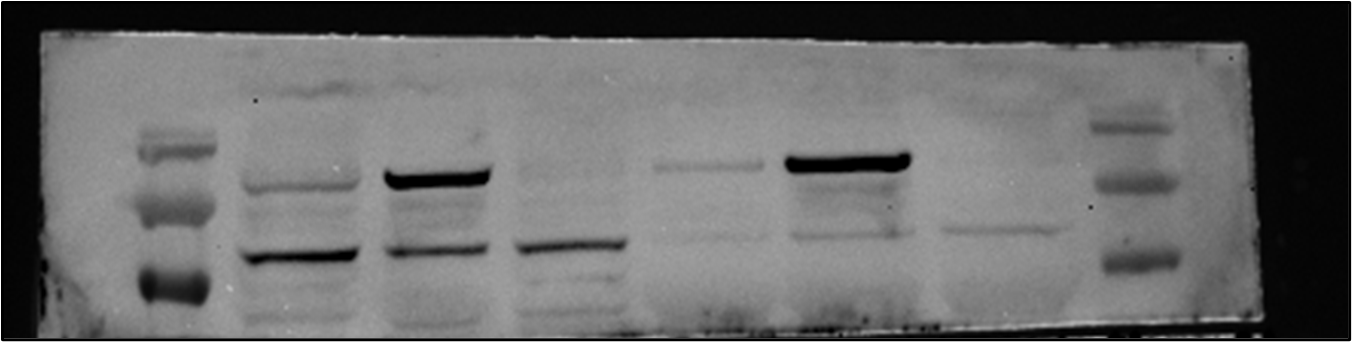


Fig. S2G

Fig. S2B


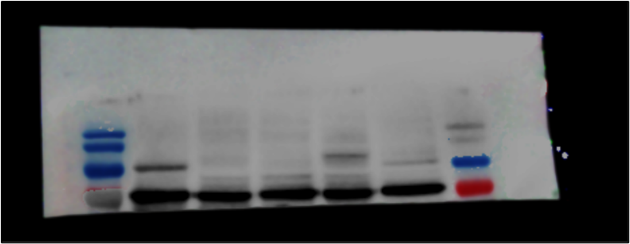

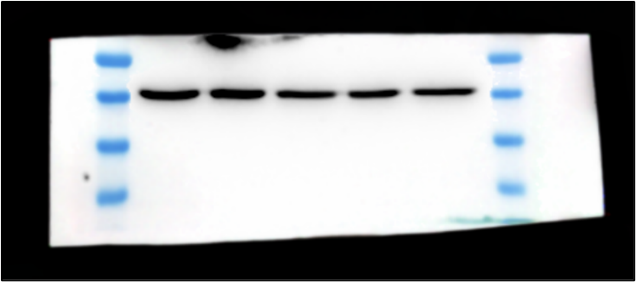


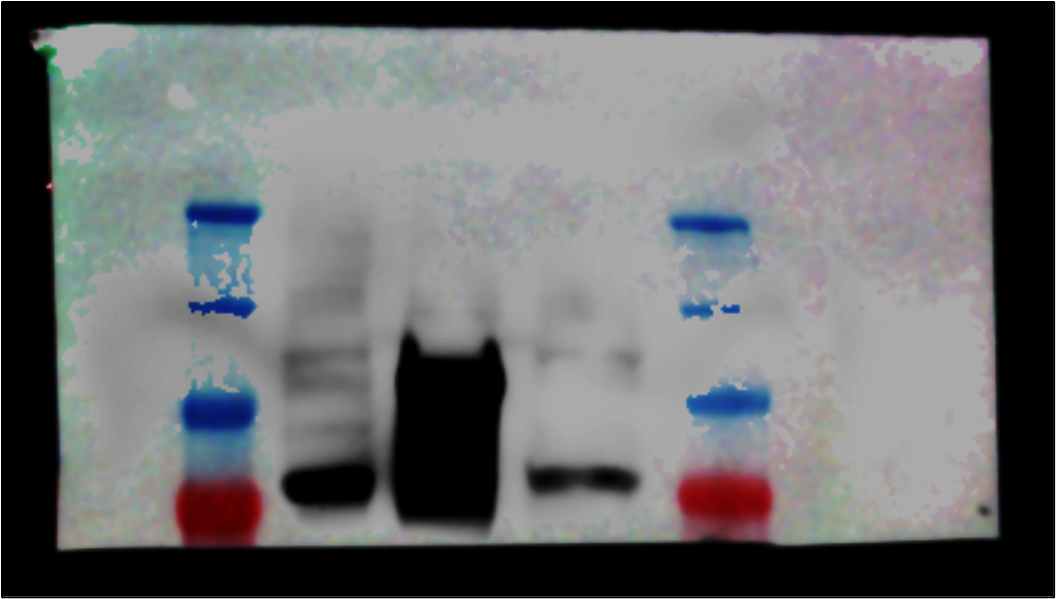

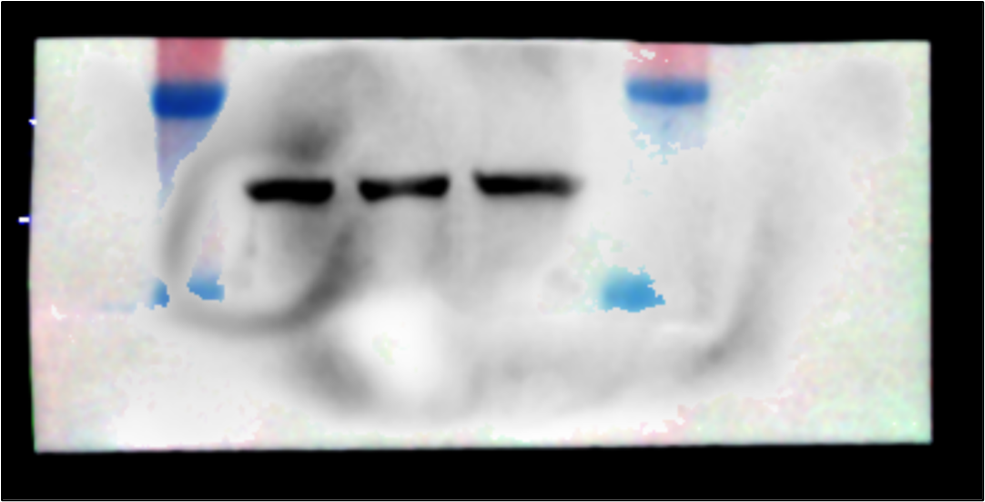

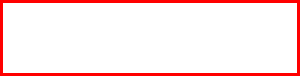


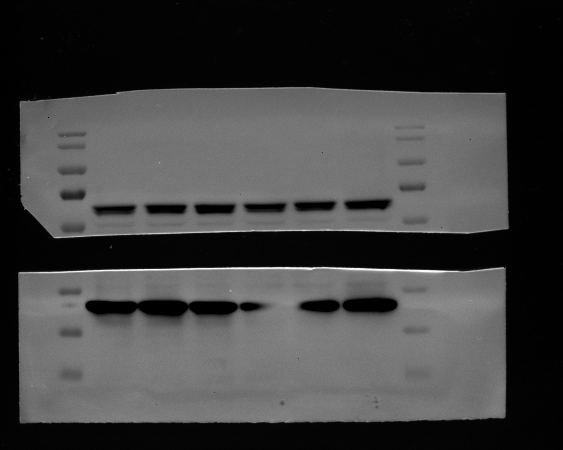

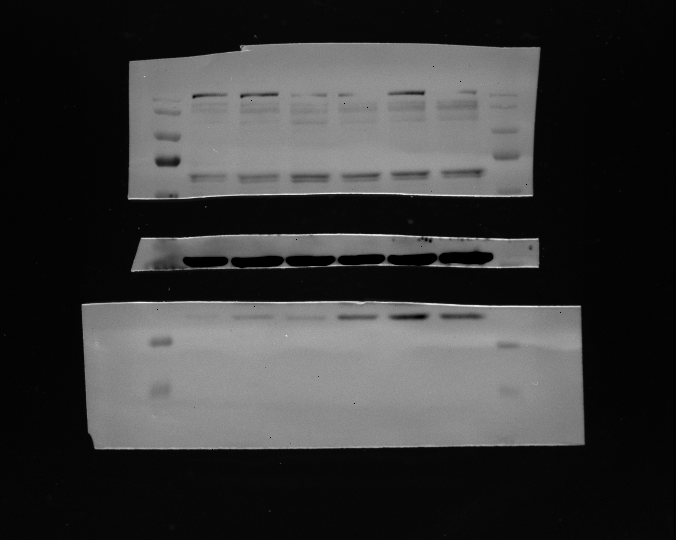


Fig. S3A


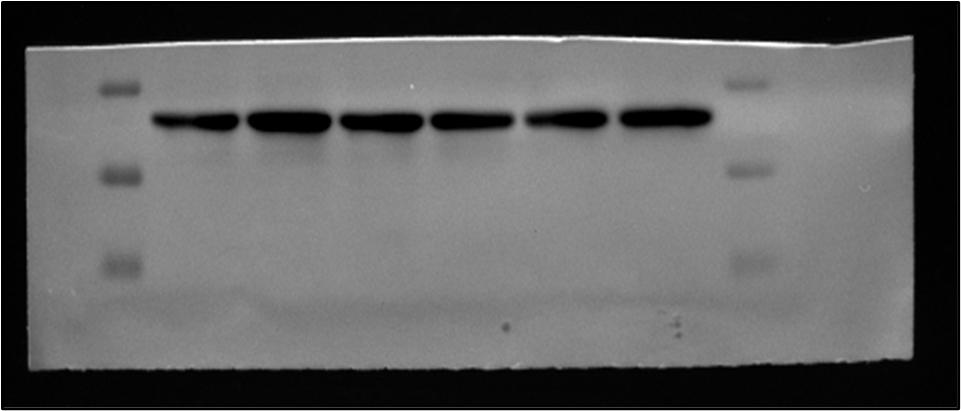


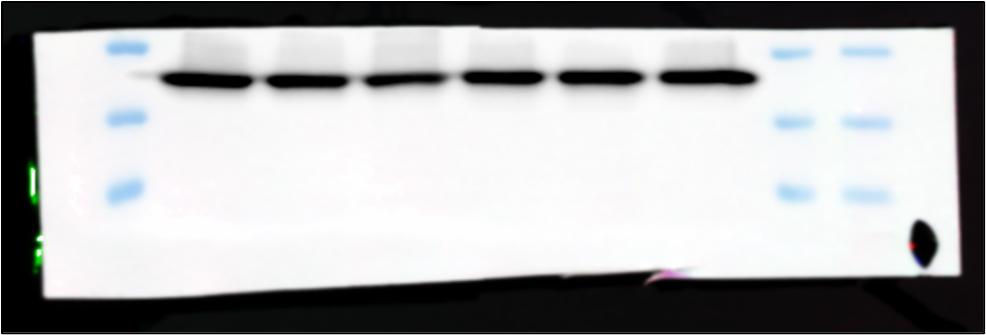

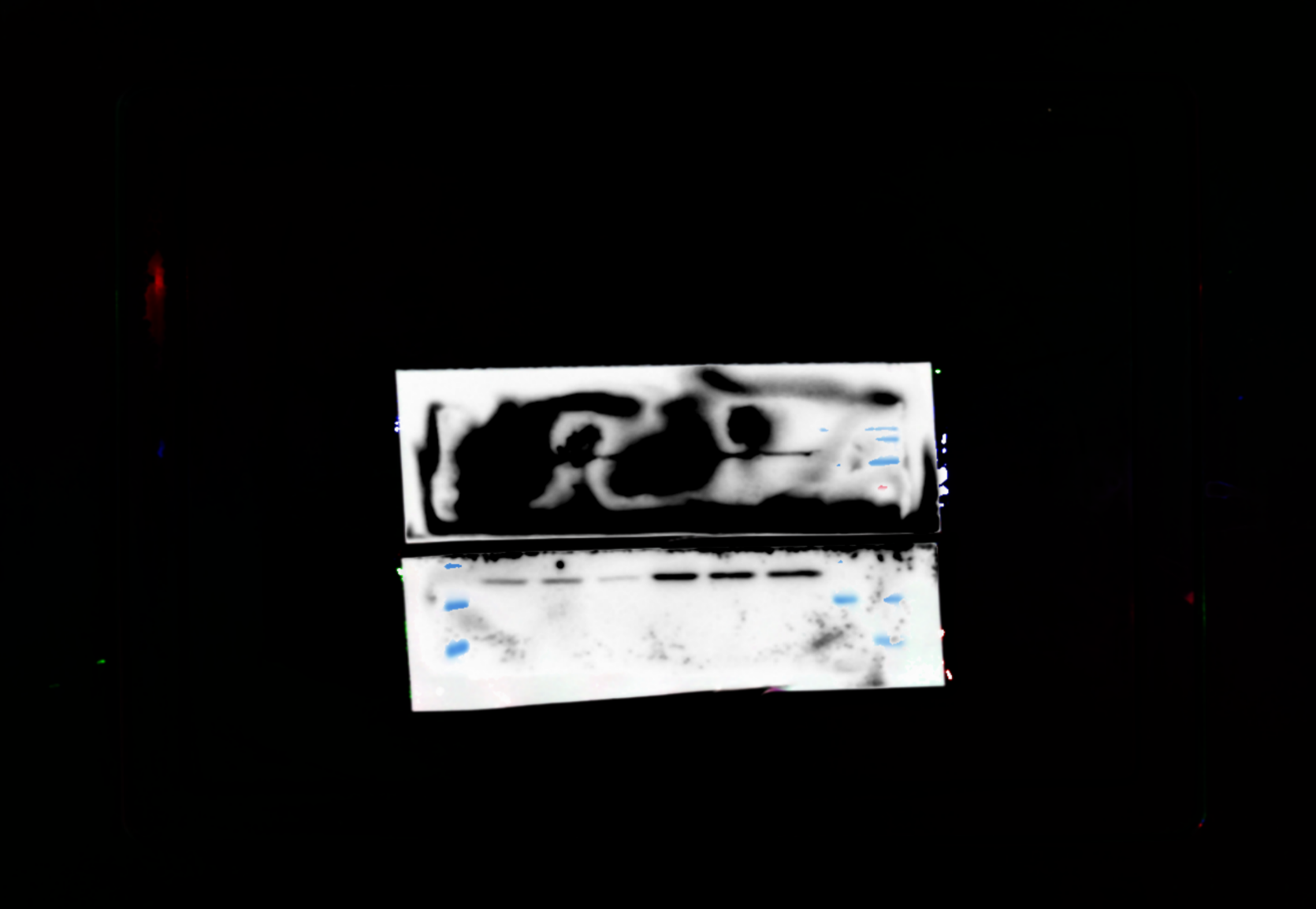


Fig. S3B


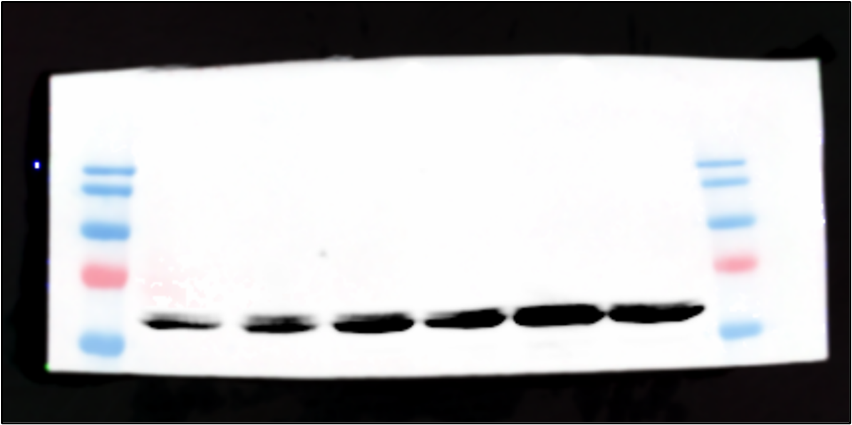

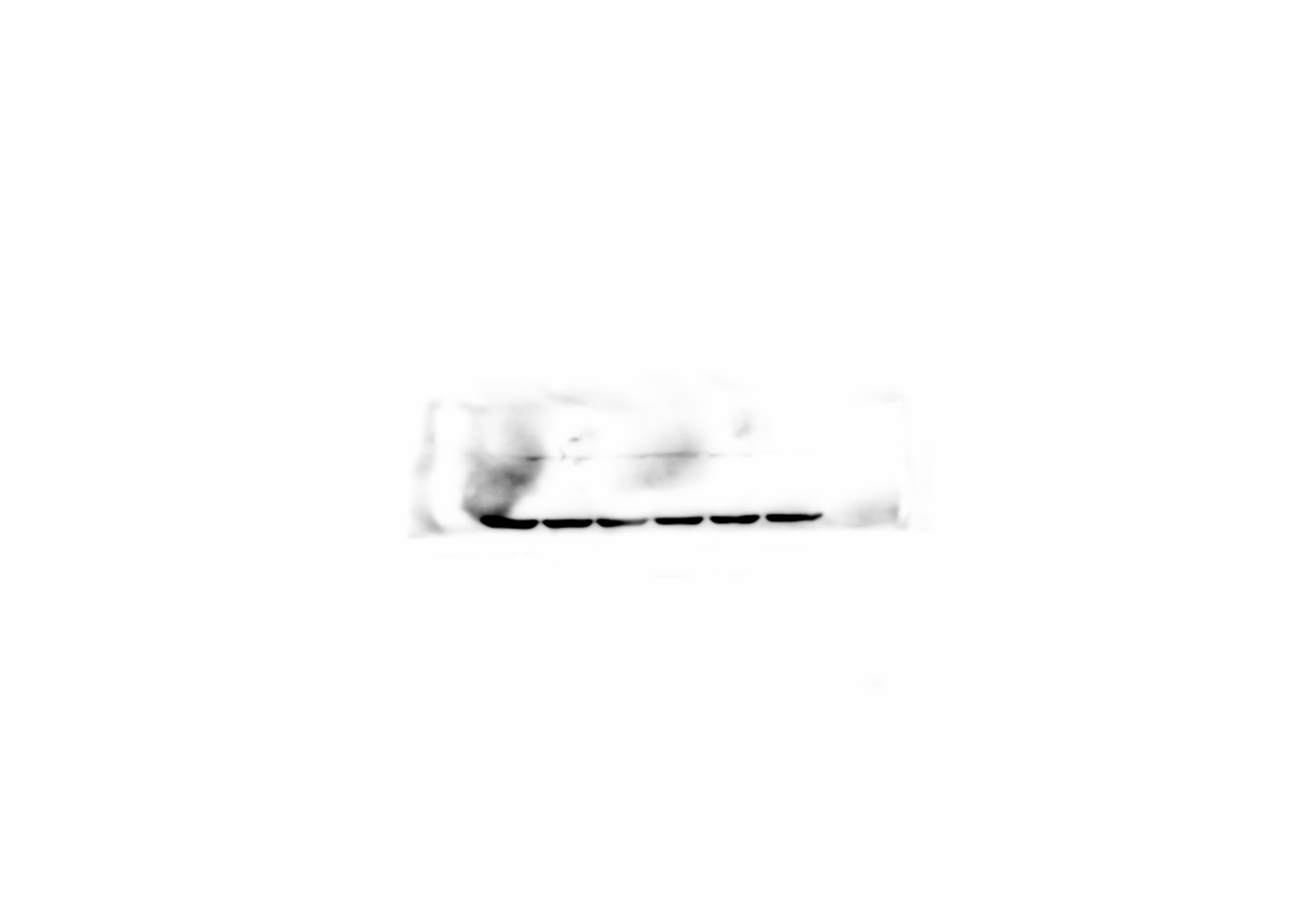


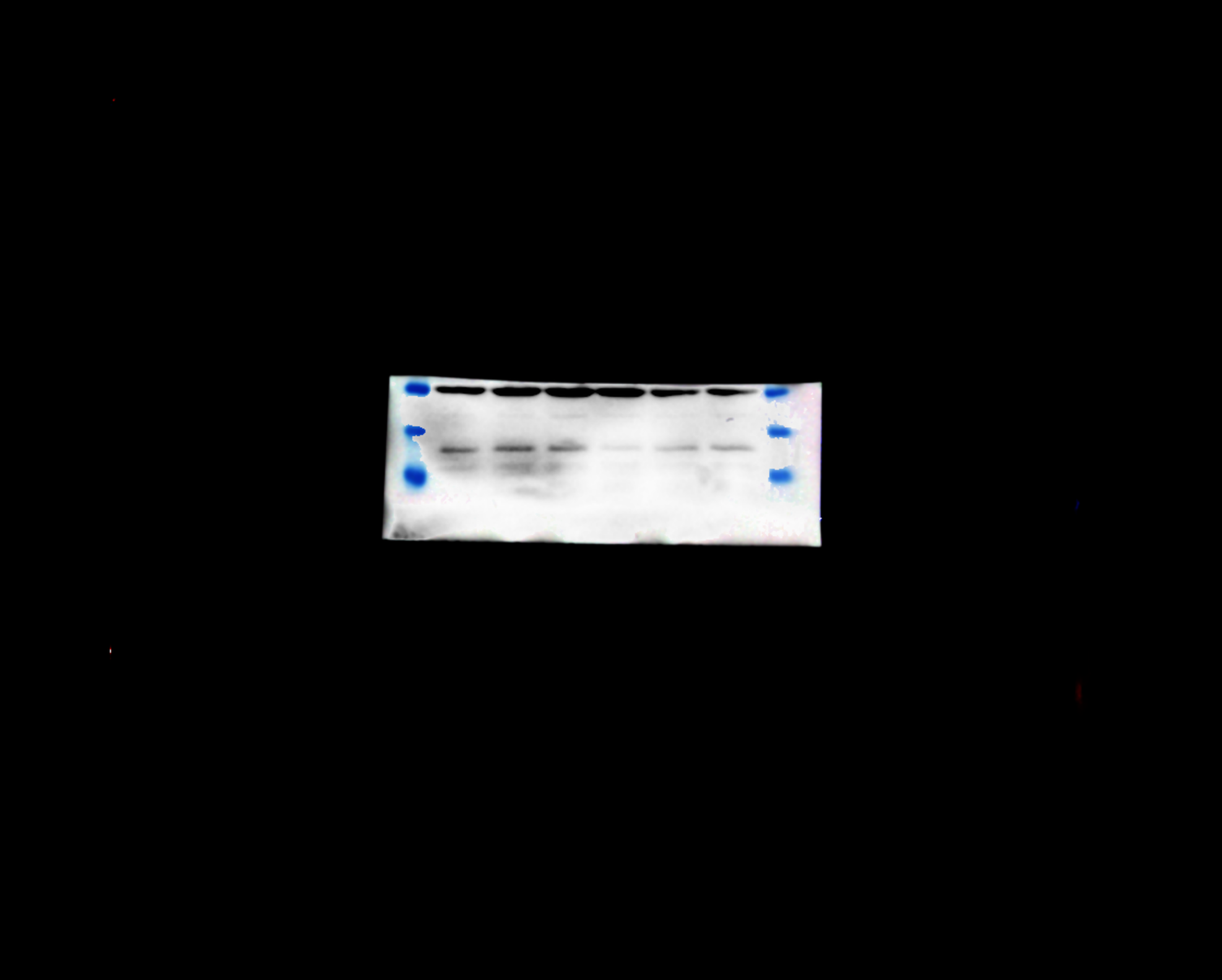

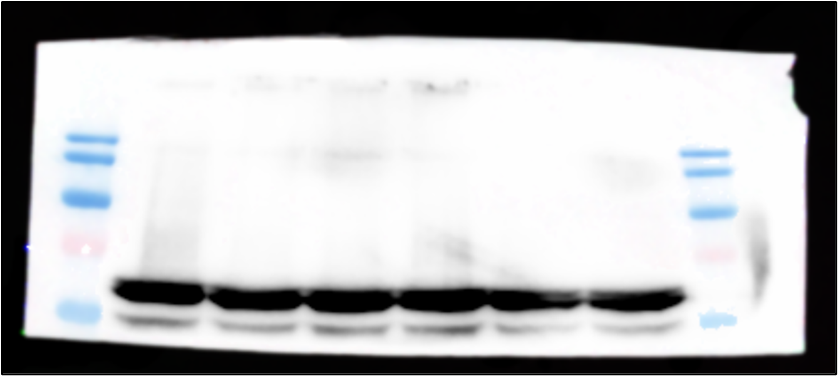


Fig. S5 B


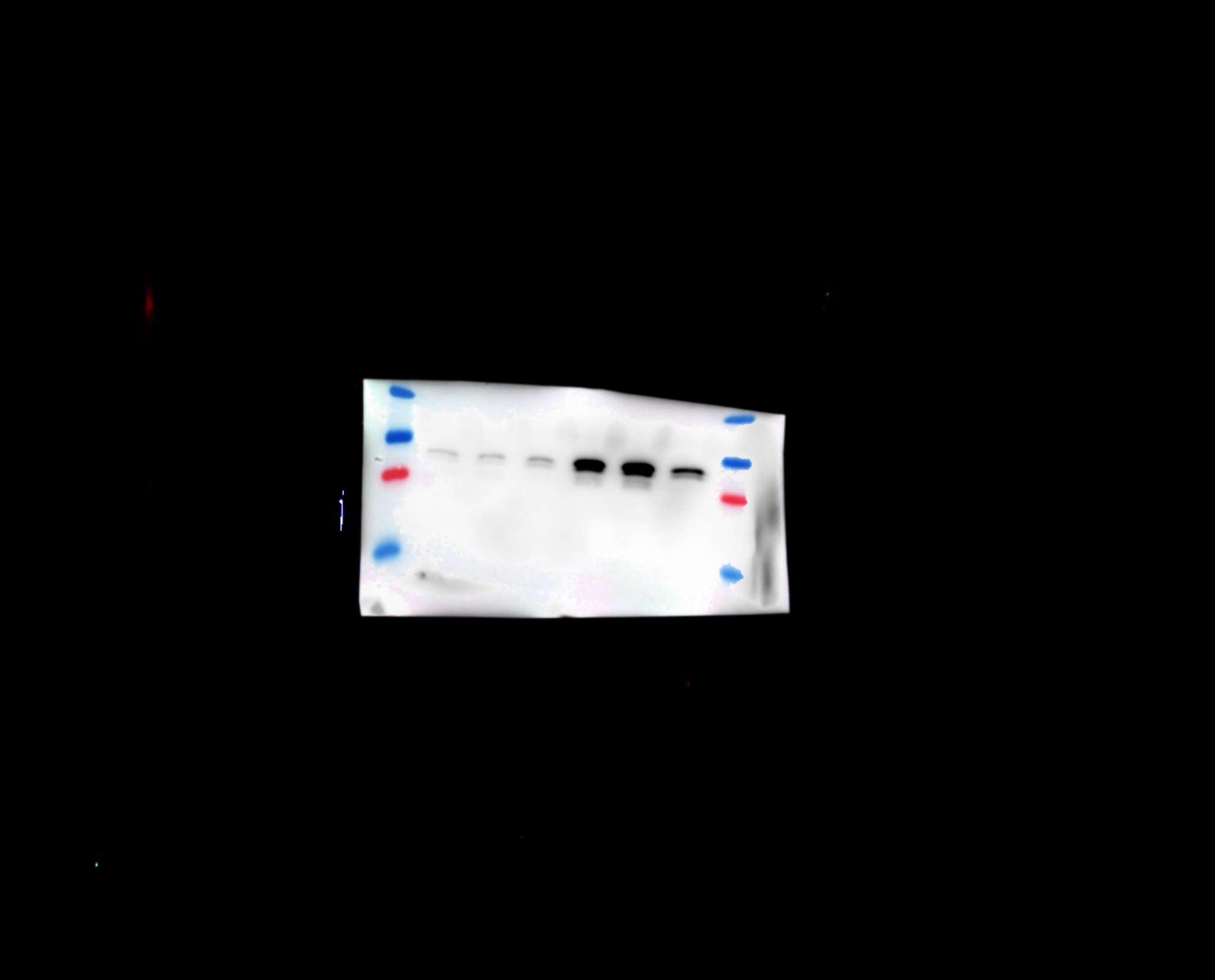


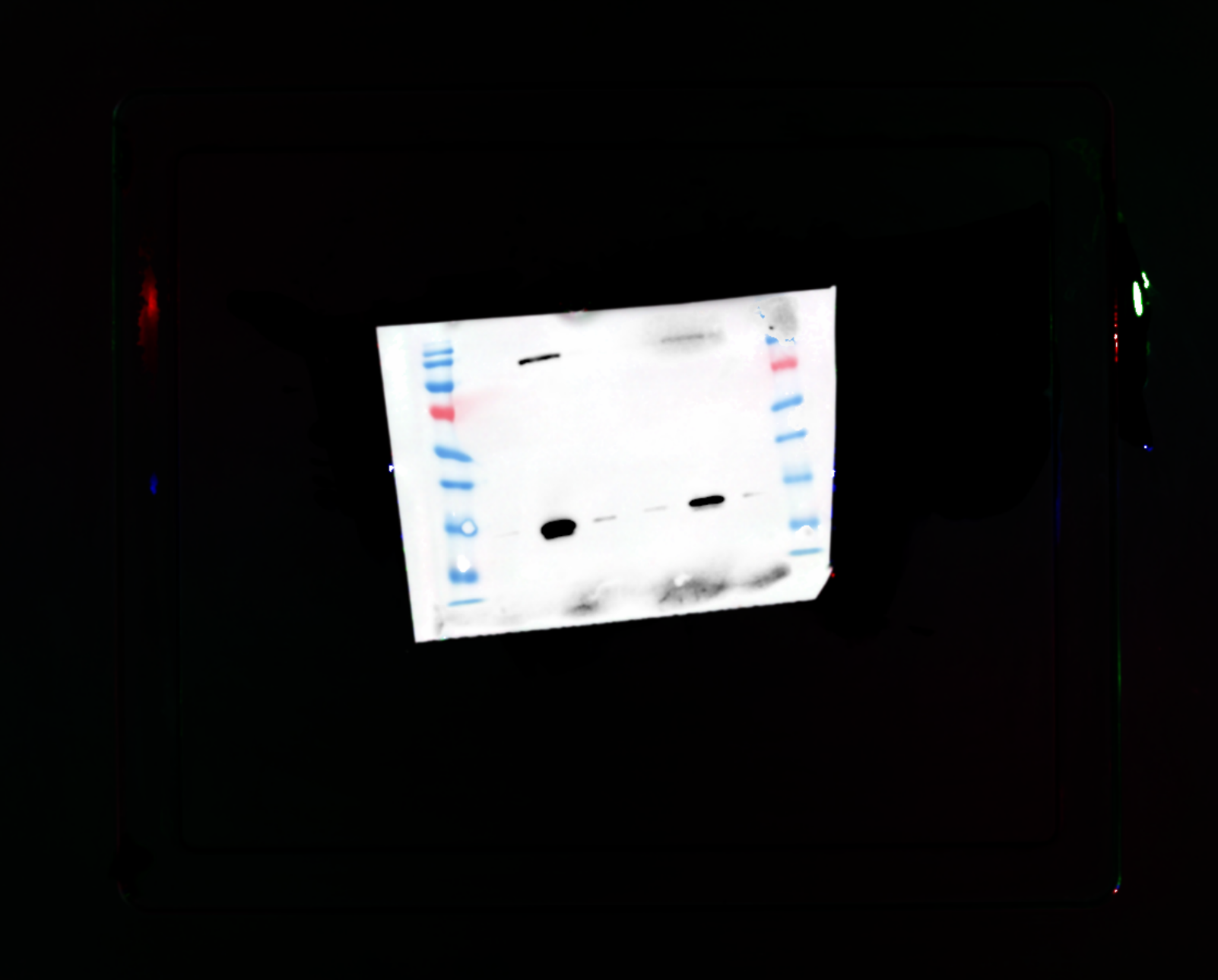

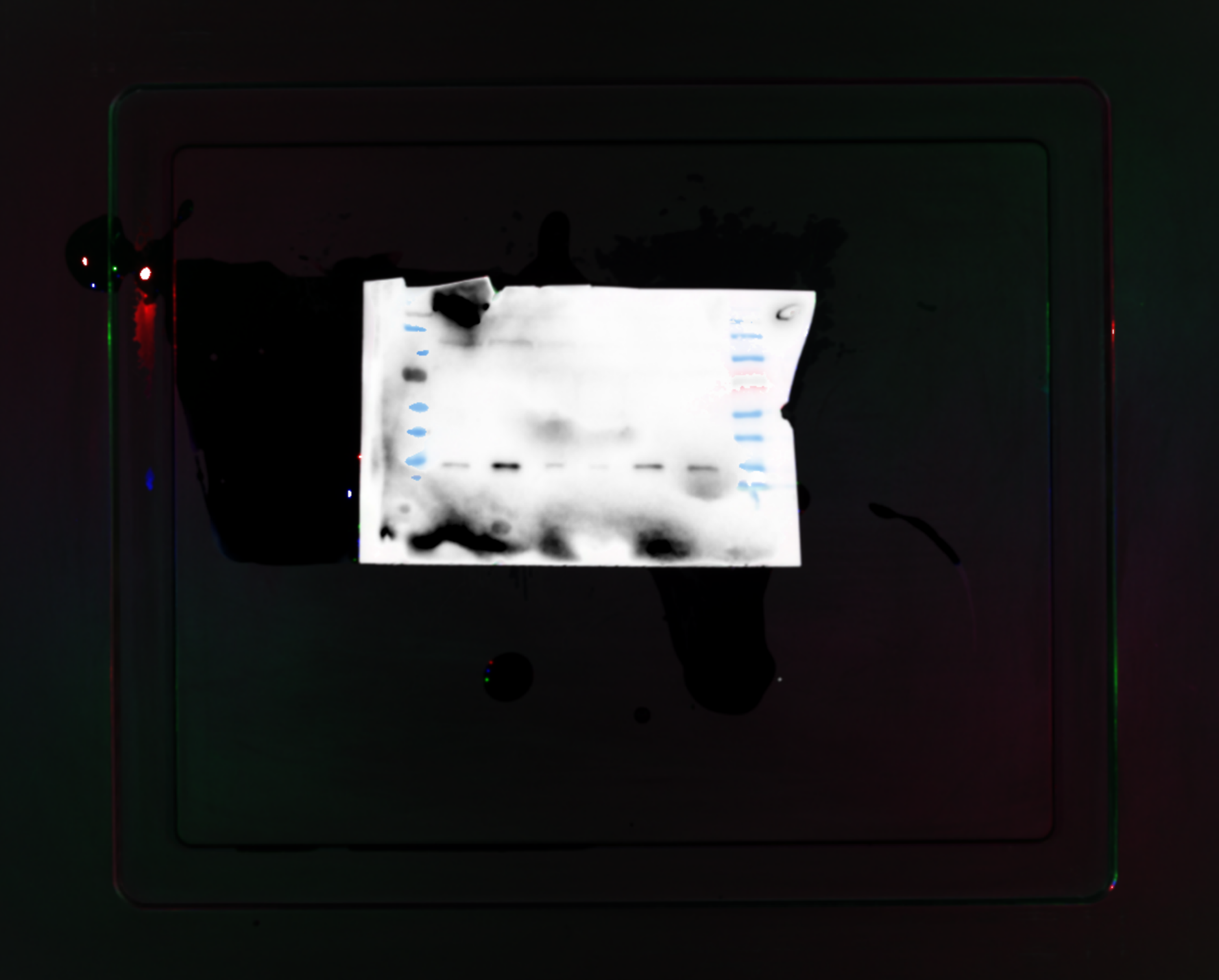


Fig. S6B


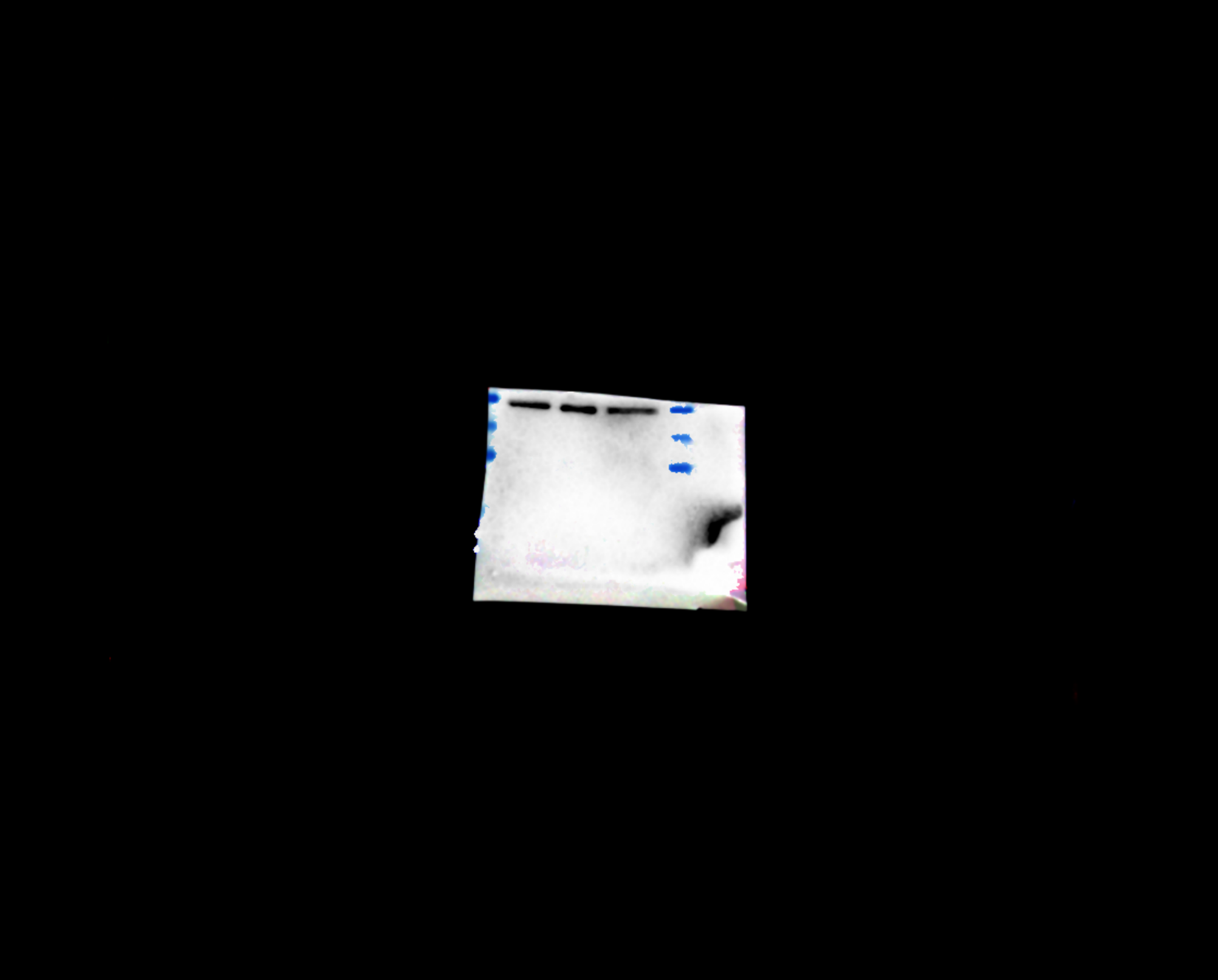


Fig. S6D


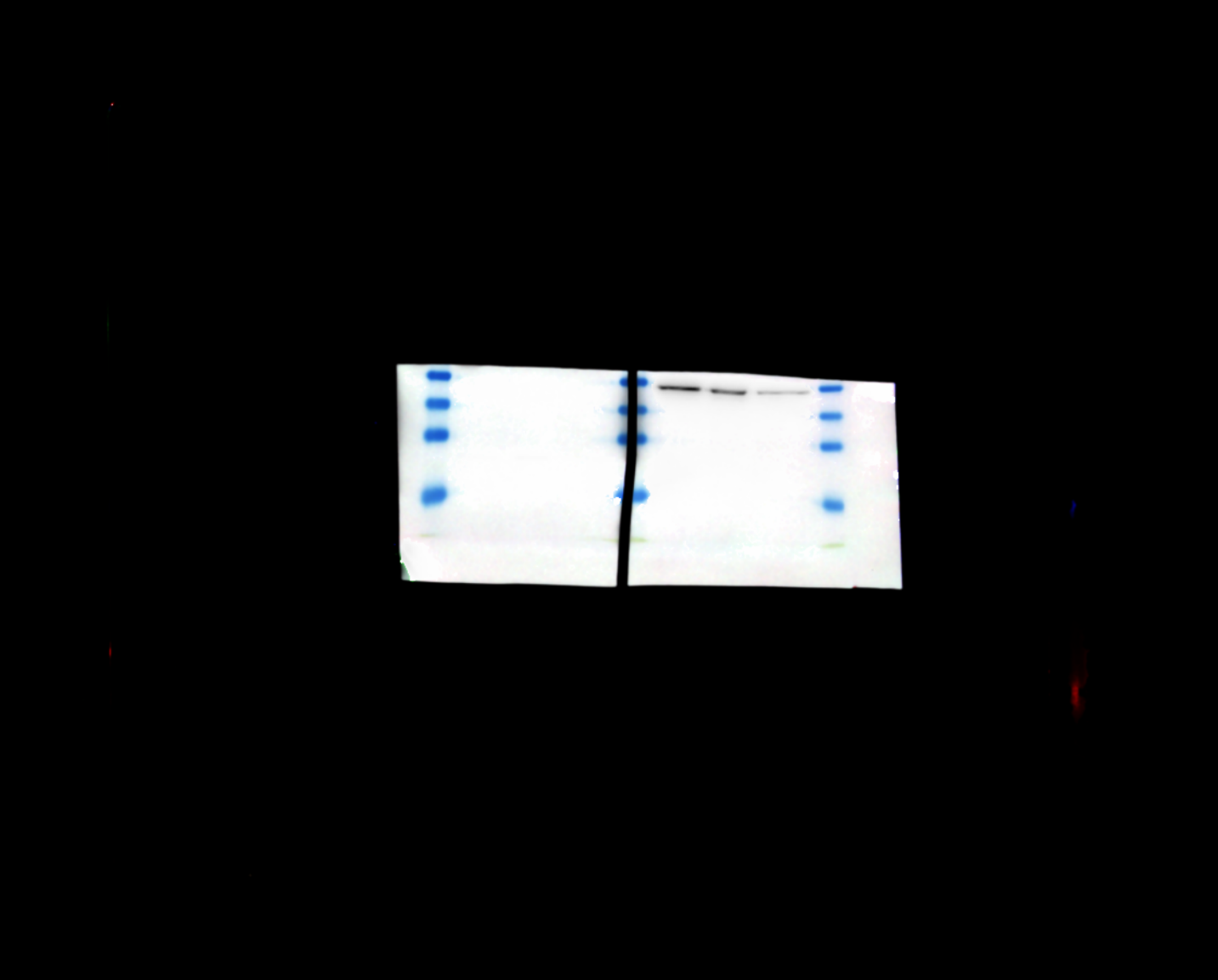


Fig. S6E


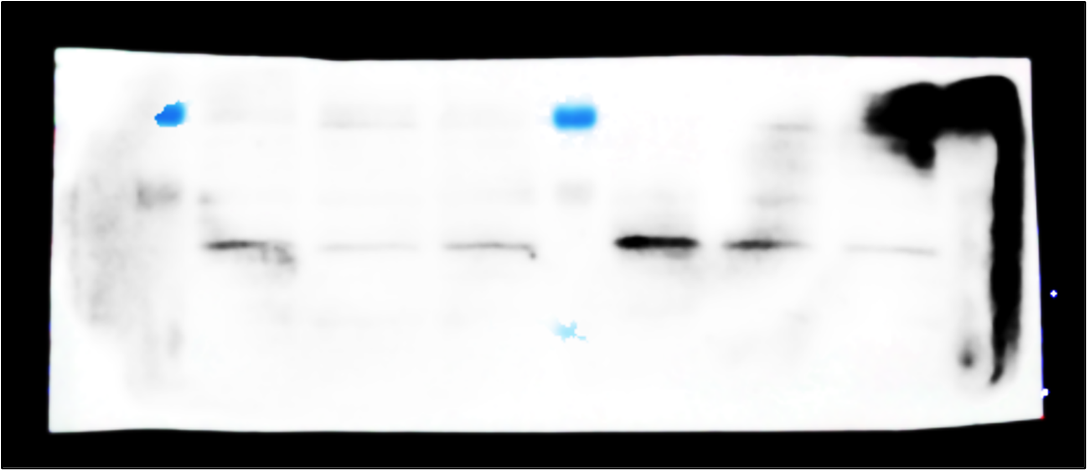


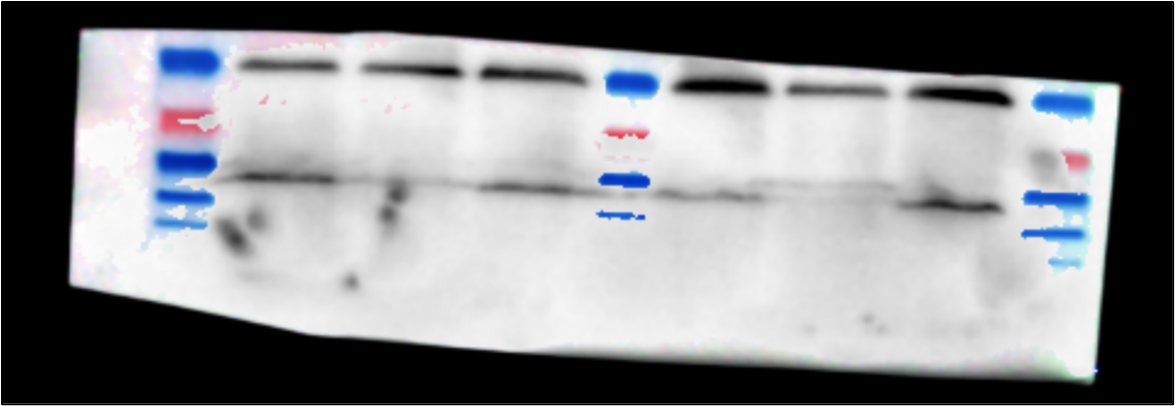


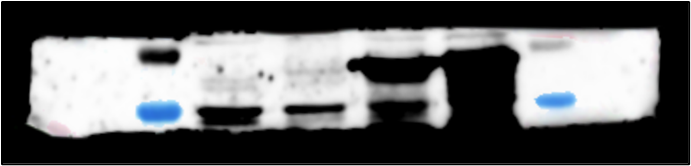

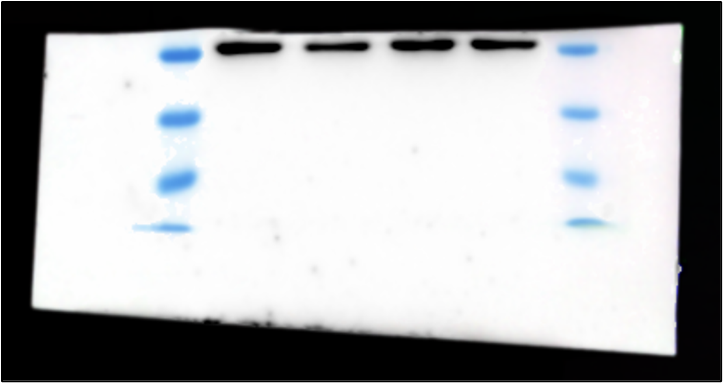

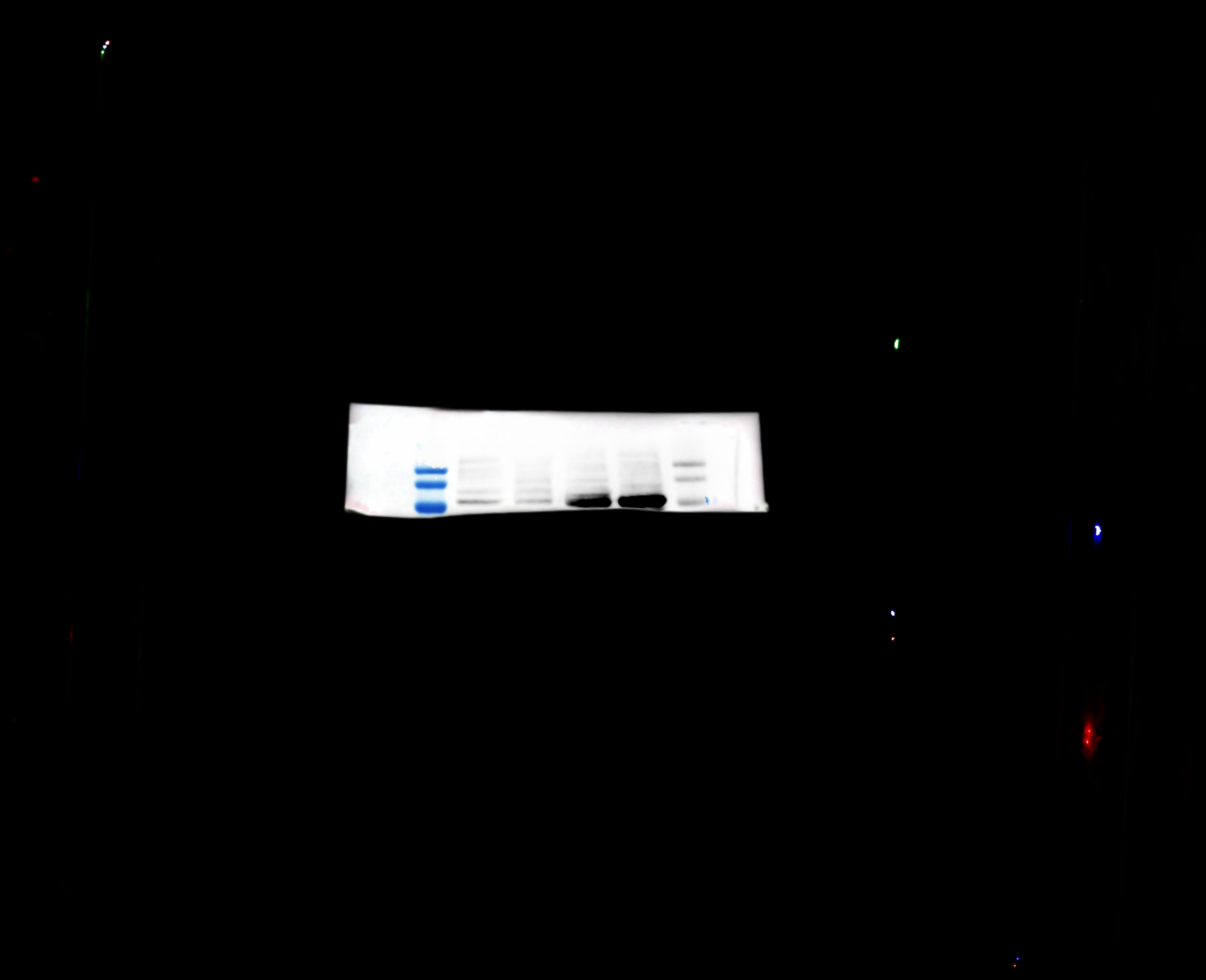

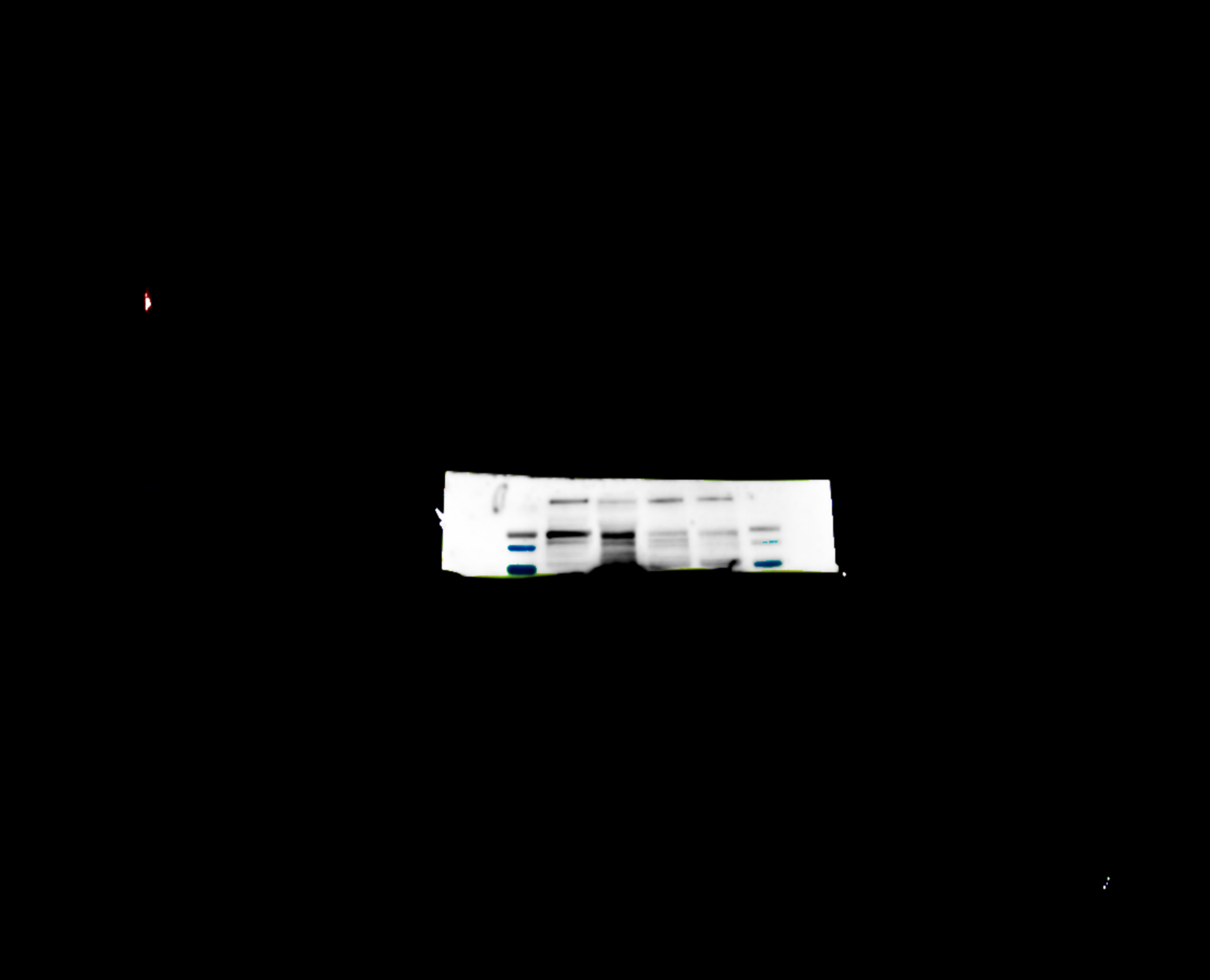


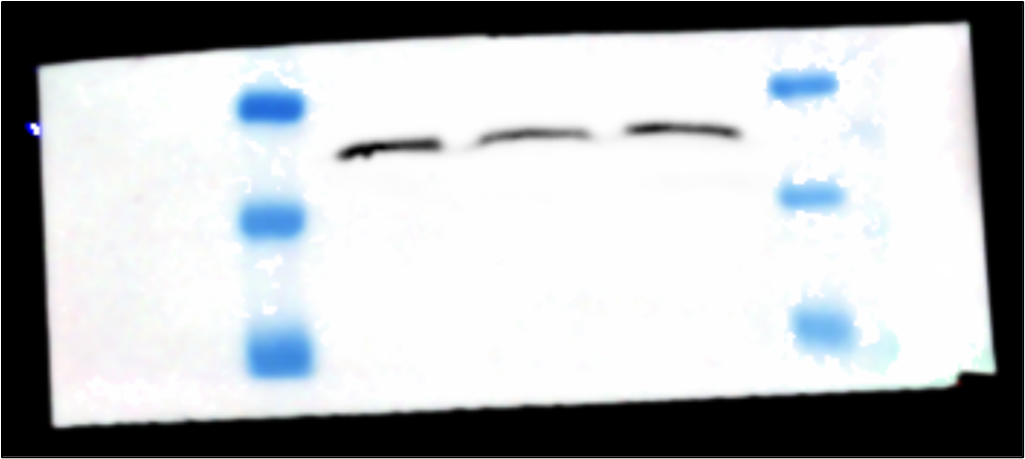

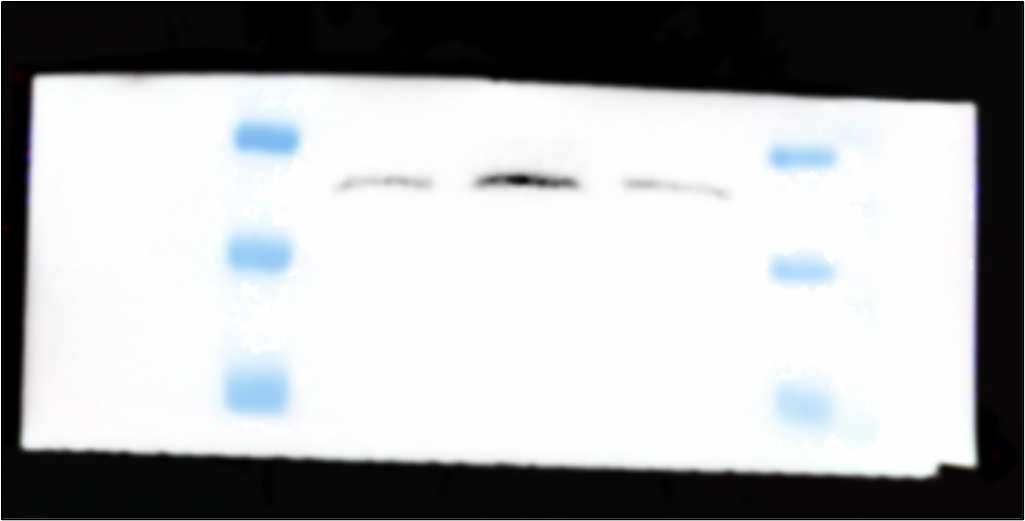

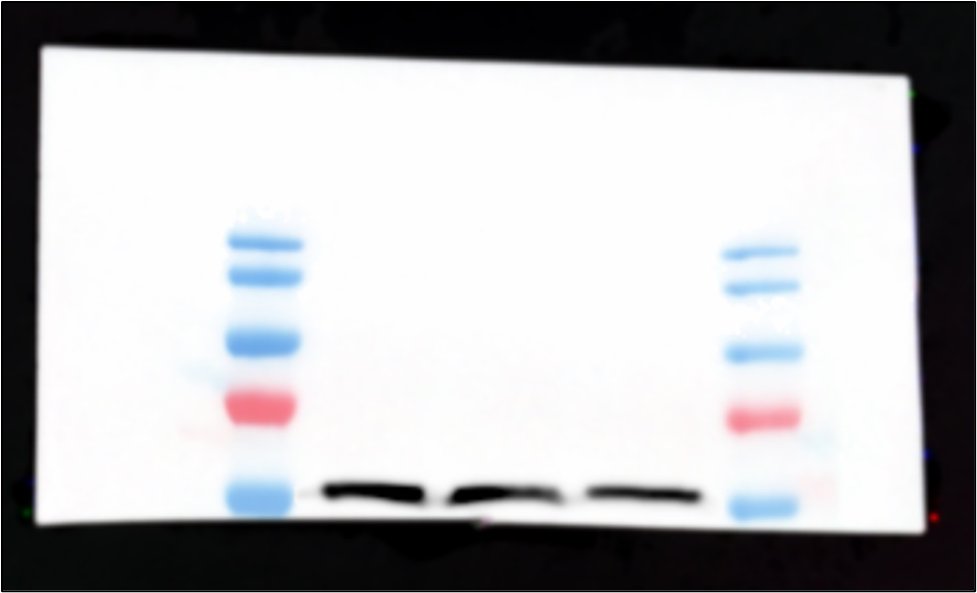


Fig. S9C

Fig. S9A


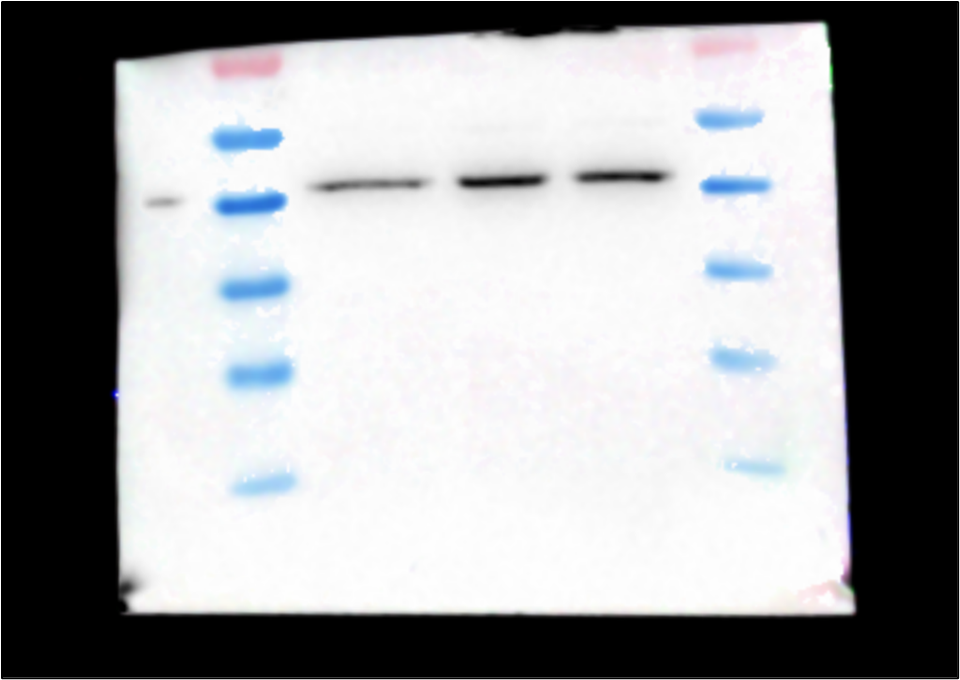

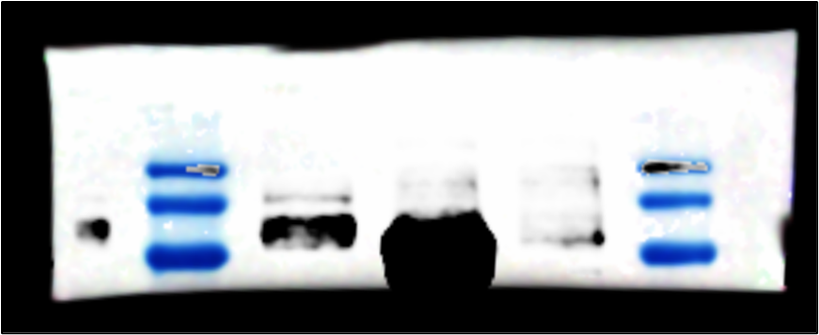

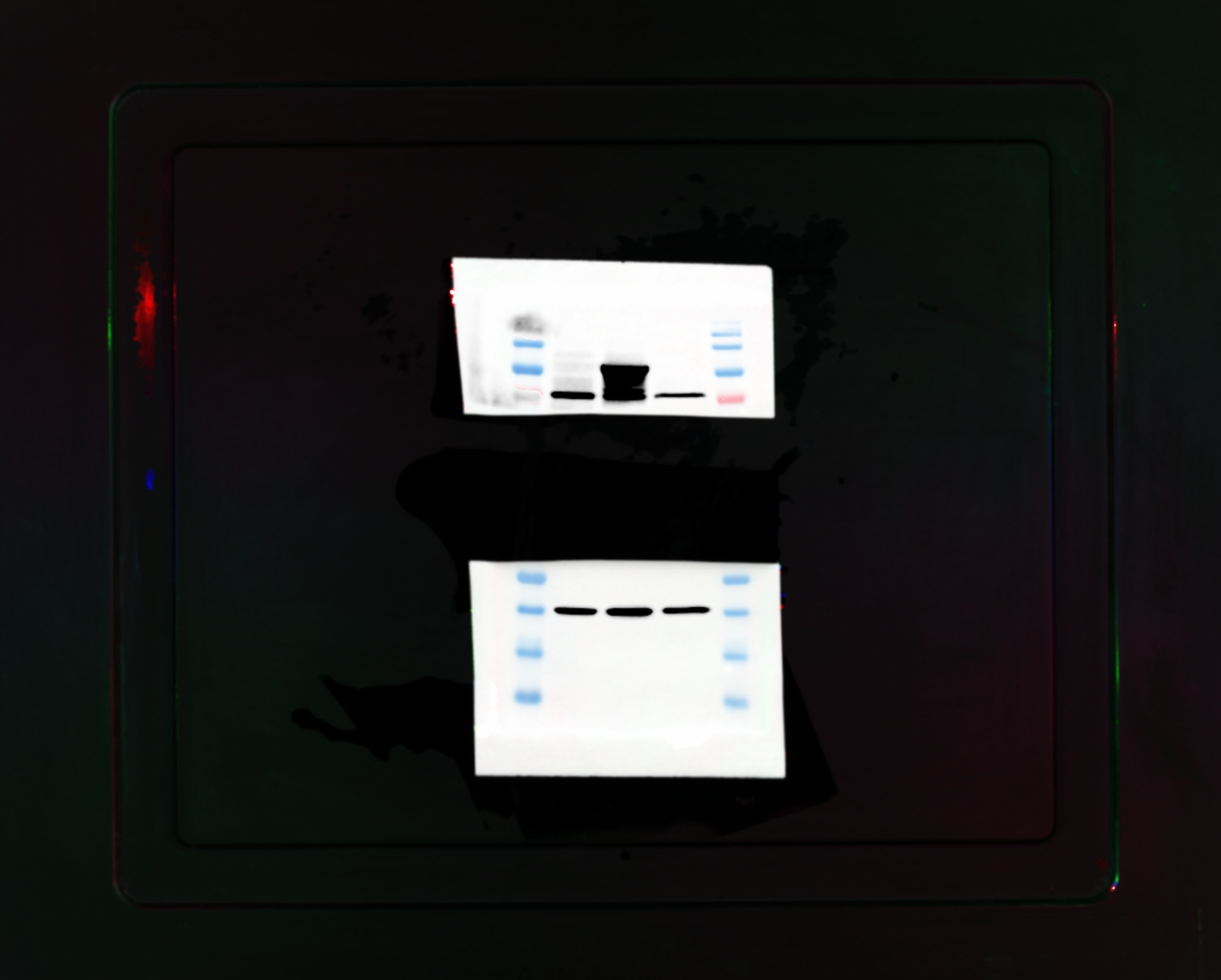

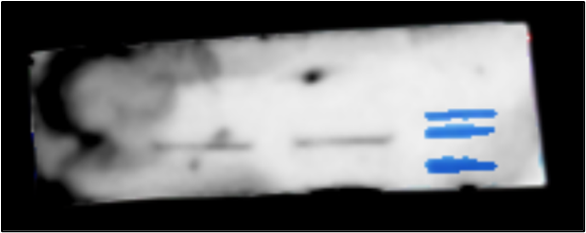

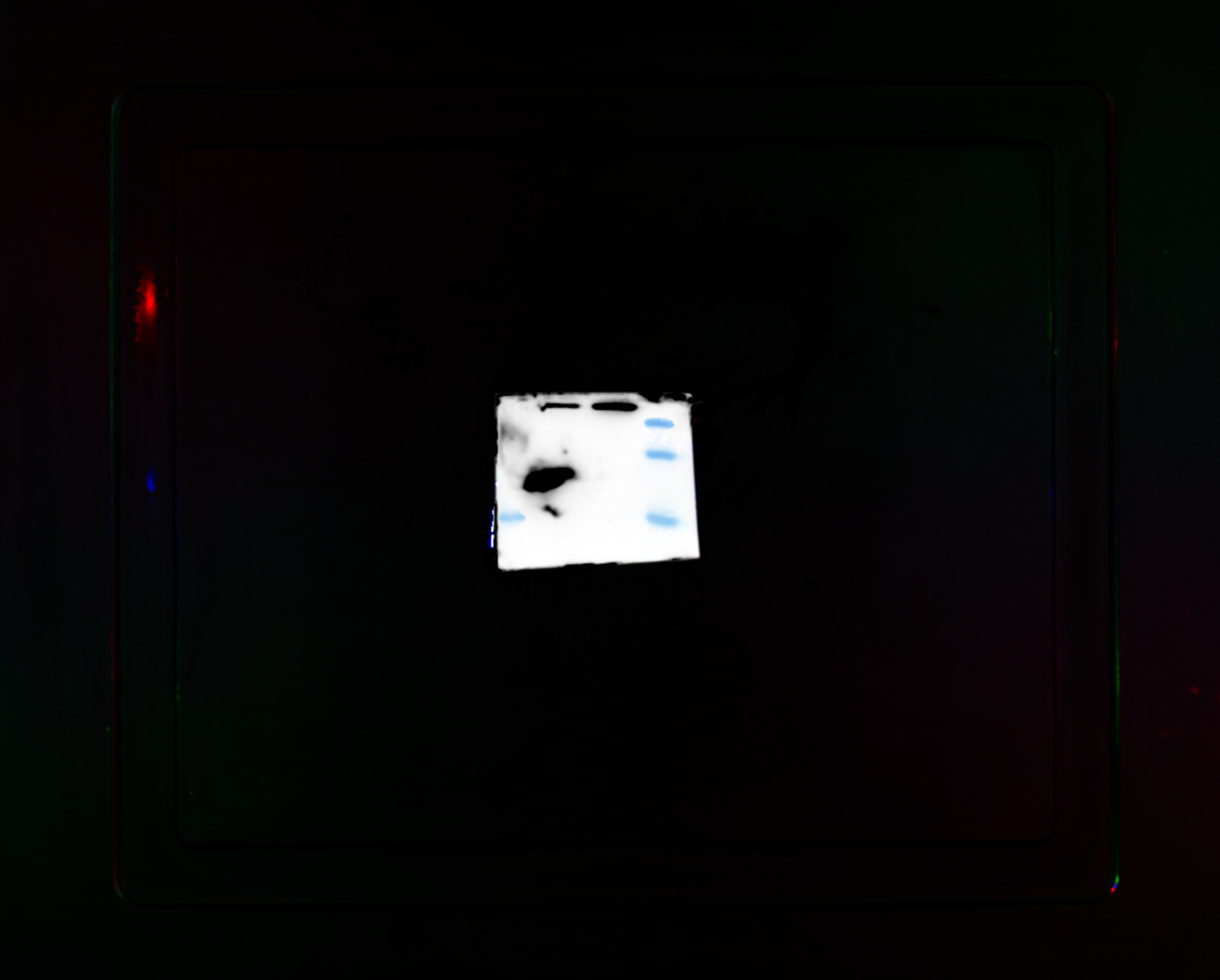

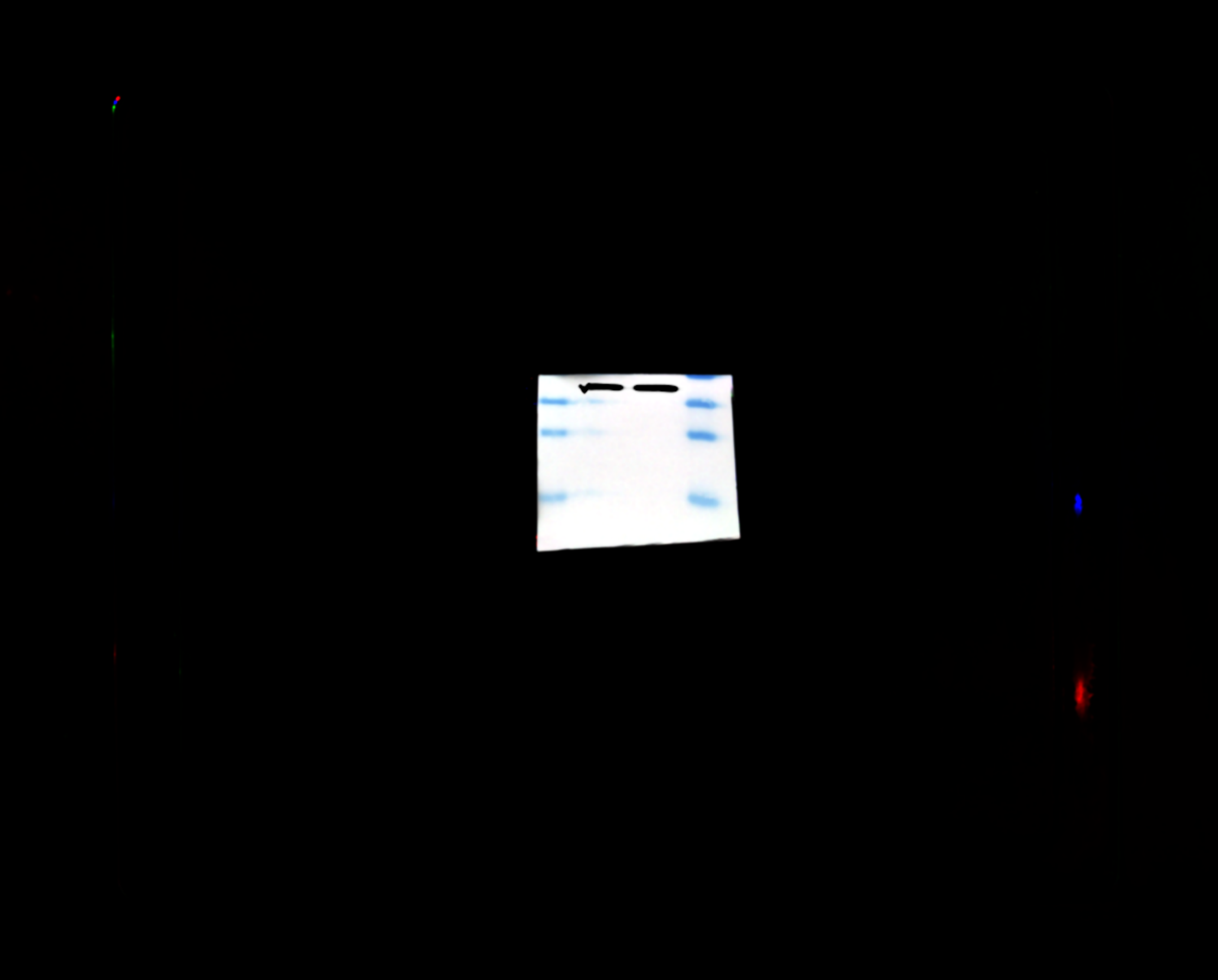


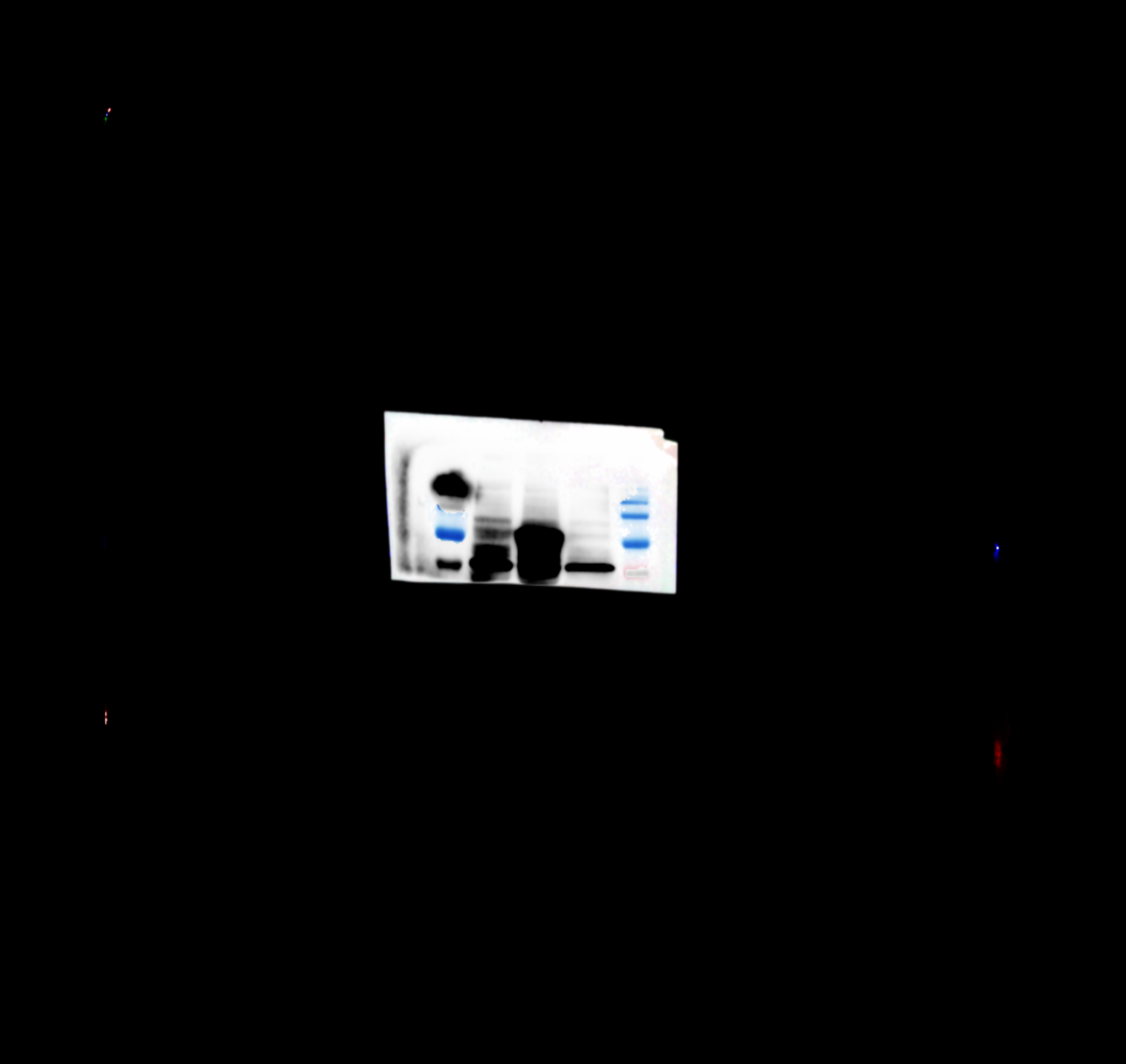

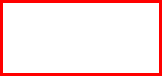


Fig. S9F

Fig. S9C
